# The Hedonics of Debt

**DOI:** 10.3389/fpsyg.2020.537606

**Published:** 2020-11-17

**Authors:** Faith Shin, Dov Cohen, Robert M. Lawless, Jesse L. Preston

**Affiliations:** ^1^Department of Psychology, University of Illinois at Urbana-Champaign, Champaign, IL, United States; ^2^College of Law, University of Illinois at Urbana-Champaign, Champaign, IL, United States; ^3^Department of Psychology, University of Warwick, Coventry, United Kingdom

**Keywords:** debt, financial decision-making, duration neglect, credit, pain of paying, hedonics, peak-end, loan

## Abstract

Psychologists and economists often discuss the “pain” of paying for our purchases. Four experiments examine how people evaluate prospective debt payments, analyzing how different features of a loan (down payment, final payment, duration, monthly payments) affect willingness to accept the loan. Akin to previous findings on physical pain, participants exhibited duration neglect and overweighted final moments. However, participants also focused heavily on the monthly or average payment (unlike in retrospective studies of physical pain where only peak-end moments seem to count). In Experiment 2, participants’ willingness to accept the loan was not significantly diminished by making it more expensive through keeping the same monthly payment but extending the length of the loan by 40% (evincing duration neglect). Further, in Experiments 3 and 4, we show that participants *increased* their willingness to buy if loans were made longer and more expensive by adding smaller, less “painful” payments to the end.

## Introduction

Collectively, Americans owe $14.2 trillion in household debt—or about 76% of the country’s yearly productive output as measured by GDP (Federal Reserve, 2020). Taking on debt is neither good nor bad *per se*. However, judging from the approximately one million persons who file for bankruptcy every year in the United States (Foohey et al., 2016), it is likely that many people have gotten in over their heads and have debt loads that are extremely burdensome.

Social and personality psychologists have mostly studied why people become heavily indebted by focusing on individual differences. In addition to economic circumstances, high levels of debt have been attributed to various individual differences in, for example, considering future consequences (Lea et al., 1995; Joireman et al., 2005), intelligence (Yang and Lester, 2016; Ganzach and Amar, 2017), impulsivity (Ottaviani and Vandone, 2011), all of the Big 5 traits, though not consistently (Nyhus and Webley, 2001; Kuhnen et al., 2013; Brown and Taylor, 2014; Harrison and Agnew, 2016), and to attitudes (about money, greed, materialism, risk, or debt itself) (Webley and Nyhus, 2001; Watson, 2003; Norvilitis et al., 2006; Garðarsdóttir and Dittmar, 2012; Liao and Liu, 2012; Seuntjens et al., 2016). In this paper, however, we take a different tack, drawing on work on how more general biases in perception may make debt—a major source of stress in American life (American Psychological Association [APA], 2019; Tay et al., 2017)—seem prospectively more palatable.

Previous research on heuristics and biases has demonstrated some reasons why people make financial trade-offs that do not maximize their long-run economic interest. For example, such research investigated why people choose to repay debts with the smallest balance rather than those with the highest interest rate (Amar et al., 2011; Besharat et al., 2014; Besharat et al., 2015), anchor on minimum payments or extended payment plans for their credit cards (Navarro-Martinez et al., 2011; Hershfield and Roese, 2015; Hershfield et al., 2015; McHugh and Ranyard, 2016), or greatly underestimate compound interest for both saving and debt accounts (Stango and Zinman, 2009; Levy and Tasoff, 2017). Consumer lenders exploit and capitalize on these and other biases (Ru and Schoar, 2016), which may be one reason why people take on too much debt. The present paper adds to this literature by examining how people evaluate extended loan sequences and how the hedonics of debt—i.e., the prospective pain associated with debt—can lead people to evaluate credit arrangements in ways that are economically disadvantageous.

In four experiments, we investigate how people prospectively evaluate debt offers and which features of loans (down payment, final payment, duration, monthly payments) affect their evaluations. We find that people tend to overweight final payments and ignore the duration of loan sequences, thus choosing debt plans where they end up paying more rather than less. Additionally, people strongly focus on the monthly payment, often to the exclusion of other costs, and together these heuristics may lead Americans to prospectively evaluate costly debt arrangements in favorable ways.

## Hedonics of Debt Payment

The hedonics of most consumer purchases are such that there is pleasure from consumption but psychological “pain” in parting with cash (Prelec and Loewenstein, 1998). Thus, purchasing a good, such as a car, induces feelings of enjoyment, satisfaction, and status, but these feelings are also coupled with the pain of having to pay for it. However, this pain of paying has been largely transformed for Americans, beginning with the credit revolution of the 1920s that changed how individuals paid for consumer goods (Murphy, 1995; Calder, 2001; Hyman, 2011). Credit cards and other debt plans can increase willingness to pay (Feinberg, 1986; Prelec and Simester, 2001), in part because the pleasure of consumption is disassociated from the pain of paying. However, debt also means that consumers no longer experience an immediate one-time payment but rather a sequence of payments as debt is gradually paid off. Such sequences are often hard for people to evaluate in a way that maximizes their wealth.

The present research examines how people make prospective evaluations of loan sequences and which features of a loan are most attended to in their assessments. We begin with Kahneman and colleagues’ peak-end rule as a starting point for our hypotheses. According to the peak-end rule, people evaluate an experience not by additively summing up pleasures or pains over time but by instead averaging the hedonics of the experience at its peak moment and at its end. This means that when people evaluate experiences, they are relatively insensitive to the hedonics at average or non-peak moments and to the duration of the experience (called “duration neglect”). This can lead people to actually prefer experiences where they cumulatively suffer more pain over those where they cumulatively suffer less (Fredrickson and Kahneman, 1993; Kahneman et al., 1993; Redelmeier and Kahneman, 1996; Fredrickson, 2000).

In the famous colonoscopy experiment, patients preferred longer colonoscopies with somewhat less painful endings to shorter colonoscopies with somewhat more painful endings (Redelmeier and Kahneman, 1996), evincing both duration neglect and an “overemphasis” on final moments. As Kahneman has remarked, “For survival you really don’t need to put a lot of weight…on the duration of experiences. It’s how bad they are and whether they end well…That is really the information you need for an organism” (NPR, 2018). What is true for physical pain may also be true for the prospective pain of paying, as people may evaluate how painful making each payment will be and pay less attention to how long the payment plan will last^1^.

Taking peak-end as a starting point for hypotheses about how people evaluate payment plans, we predict that people will exhibit an “overemphasis” on final payments (preferring hefty down payments at the beginning to hefty payments at the end) and will be insensitive to the length of the loan (evincing duration neglect). However, we also expected some important differences from the peak-end rule as well. In contrast to the peak-end rule, which postulates that people are relatively insensitive to average, non-peak moments, we predicted that people are strongly influenced by these moments in evaluating credit plans, focusing a great deal on the regular monthly payment (likely also because of cash-flow concerns) (Juster and Shay, 1964; Walker and Sauter, 1974; Seaton and Vogel, 1980; Hoelzl et al., 2011; Stango and Zinman, 2011)^2^. As Bettman et al. (1998) note, it is crucial to understand a decision maker’s focus of attention, and monthly payments (in addition to final payments) seem to grab a huge share of this attention, causing people to sometimes completely miss other important features of the problem^3^.

We tested these hypotheses in four experiments. In Experiments 1 and 2, we examined whether people prefer loans of varying durations, monthly payments, final payments, or down payments. We found that people are less likely to take into account the duration of the loan but are more sensitive to final payments (end of the loan) and monthly payments. We extended these findings and, in Experiments 3 and 4, tested whether participants prefer more expensive loans, if smaller, less “painful” payments are added to the end of the contract (a la Kahneman et al., 1993). We predicted that because people ignore duration and weigh the ending so heavily, a loan sequence which is lengthened by *adding* a somewhat less expensive payment at the end should be preferred to a shorter sequence without this additional payment. According to the peak-end rule, even though it involves more “objective” pain, adding a somewhat less expensive monthly payment at the end should make the sequence more desirable (Kahneman et al., 1993; Redelmeier and Kahneman, 1996; Diener et al., 2001). To our knowledge, Experiments 3 and 4 are the first demonstrations of this principle applied to lending, in which consumers become (slightly) more willing to take on a loan if additional payments are added to the end. To our knowledge, this paper is also the first demonstration of duration neglect with respect to lending, such that with a given monthly payment, the length of the loan can be stretched out with little to no effect on consumers’ willingness to assume the debt burden.

### Between- and Within-Subject Designs

It should be noted that there have been various explanations for why peak-end and duration-insensitivity phenomena might be observed, ranging from, for example, conversational norms to memory biases to the meaningfulness of an experience’s end or resolution (Rozin and Stellar, 2009; Kahneman, 2011; Xu et al., 2011; O’Brien and Ellsworth, 2012; Blanchard et al., 2014; Schafer et al., 2014; Tully and Meyvis, 2016; Rozin and Rozin, 2018; cf. Müller et al., 2019). In the present paper, we expect effects are driven primarily by attention. That is, when two payment plans are put side-by-side and they have the same monthly payment amount, participants will choose the shorter (and thus less expensive) payment plan over the longer (and more expensive) payment plan. Such a juxtaposition makes duration easily evaluable and we would expect participants to make rational judgments (Morewedge et al., 2009; Hsee and Zhang, 2010). In between-subject designs, however, we would expect duration to be ignored or at least underweighted (Experiments 1 and 2).

Additionally, all else equal, in the absence of a side-by-side comparison, people should prefer that their loans end on a less burdensome note rather than on a more burdensome one. Thus, like participants evaluating physical pain in Kahneman et al. (1993) and Redelmeier and Kahneman (1996), our participants should more positively evaluate a sequence of loan payments, if smaller, “less painful” payments are tacked onto the end of it (Experiments 3 and 4). If payment plans are put side-by-side, the loan without the extra payments might be preferred, but judged in isolation, the longer, more expensive loan with the extra payments tacked on should be preferred.

As will be seen, duration neglect and peak-end phenomena are much more evident for participants’ first (between-subjects) judgments than for their subsequent judgments, for which they have some standard of comparison. Given that many people actually do *not* shop around for their loans (even for large loans like those for cars and houses), the between-subjects aspect of our designs is true to many (though not all) people’s lived experience (Morton et al., 2011; Woodward and Hall, 2012; Consumer Financial Protection Bureau [CFPB], 2015, 2016; Moraga-Gonzalez et al., 2015).

### Biases Impervious to Experience?

Finally, we expect that the relative inattention to duration and the desire for good endings are part of people’s basic cognitive machinery and as such are not greatly modified by experience or education (but see Strough et al., 2019). Nevertheless, there is research showing (a) that marketers are likely to target behavioral biases, particularly of people who are poor and have less education, and (b) that individuals learn from experience with various financial products. An obvious explanation for (a) is that marketers are much more hesitant to alienate the middle class with practices seen as exploitative. However, growing research has also highlighted the problems of scarcity that can cause poor people to make less “rational” decisions (Mullainathan and Shafir, 2013; but see Shah et al., 2015).

For example, there is evidence from economics and finance that institutions tailor their products in ways that take advantage of either lower-income or less-educated borrowers. Thus, lenders are more likely to advertise credit cards with heavy “backloaded” fees [late fees, over-the-limit fees, default annual percentage rates (APRs)] to less-educated borrowers (Ru and Schoar, 2016). Savings banks that target low- to middle-income households are more likely to offer complex financial products with higher markups, as compared with banks that target other clientele (Célérier and Vallée, 2017). Lenders in low-income locations were more likely to “steer” borrowers into potentially disadvantageous mortgages (Agarwal et al., 2016a) (see also Lusardi and Tufano, 2015 on debt literacy and high-cost borrowing).

There is also evidence that people learn to make better financial decisions through experience. For example, people learn how to make better mortgage rate and refinancing decisions (Agarwal et al., 2014, 2016b) as well as avoid costly credit card fees and contracts (Agarwal et al., 2008, 2015).

It is thus possible that duration neglect and the overweighting of endings could be modified with experience and education, but we do not think it likely that these countervailing effects would be large. We examined whether various factors—car buying and student loan experience, family income, household financial duties, or various individual difference variables (described in the Supplementary Materials)—would alter participants’ foci of attention in beneficial ways (e.g., Morewedge et al., 2009), but they rarely did.

## Experiment 1

### Materials and Methods

#### Overview

University students were presented with five student loan scenarios designed to test whether people are (1) sensitive to the duration of loans and (2) prefer larger payments at the beginning of the loan, end of the loan, or spread throughout the loan. To test whether people are sensitive to the duration of loans, participants were asked the maximum monthly payment they would be willing to make toward a 5, 10, or 15 years student loan (randomly assigned, between-subjects). We predicted that students would be insensitive to the duration of the loan and report similar monthly payments, regardless of its length. We also asked this question within-subjects to test whether this effect may be due to attention, rather than financial illiteracy (if so, drawing attention to loan length through within-subject questions should attenuate or remove duration neglect). Further, three questions tested whether people prefer payments weighted toward the beginning of the loan, end of the loan, or spread throughout the loan.

#### Participants

One hundred and ten participants were recruited from public places at the University of Illinois Urbana-Champaign. Two people were excluded because they indicated they were not students, leaving 108 participants (45 male, 63 female, *M*_*age*_ = 20.2 years, 45% have taken out loans).

#### Design and Materials

Participants were told that we were conducting a study about people’s opinions on student loans and some of the financial decisions that they make. Students were told that for their reference, an average student begins to make payments toward their student loans 6 months after they graduate, and the average starting salary of an undergraduate is $60,000. Participants were given five different loan scenarios with two scenarios asking them their preferred monthly payment and three scenarios asking them to choose between different student loan offers.

The study employed a mix of between-subject and within-subject designs. Scenario 1 was designed as a randomly assigned between-subjects item, asking students the maximum loan payment they would be willing to pay after they graduated from college for a 5 years student loan, 10 years student loan, or 15 years student loan. The slider ranged from $200 to $700. We predicted that students would exhibit duration neglect and report monthly payments that were not significantly different from one another, even though the loan durations could differ by factors of 2 or 3.

The next question, scenario 2, was designed as within-subjects. On the same page, all students were asked two questions about the maximum loan payment they were willing to pay for a 10 years student loan *and* a 15 years student loan. The slider ranged from $200 to $700. We predicted that when students were presented with both questions, they would correctly report a lower student loan payment for the 15 years student loan compared with the 10 years student loan.

Students were then told to assume that they have taken a new job that pays $60,000 and offers a $12,000 signing bonus and will begin to make payments on their student loans in 6 months. Students were provided this instruction to ensure that assumptions about starting salary and assets were similar. They were then asked to indicate their preference between different loans the school is offering on a 0–100 scale with each offer on opposite sides of the scale. The following three questions were presented in random order.

Scenario 3 was designed to test if students prefer a student loan with monthly payments and a final payment ($215 monthly payment for 3 years and final payment of $3,000) or a student loan with higher monthly payments and no final payment ($300 monthly payment for 3 years). We predicted that students will “overweight” the final payment and want to avoid the heavy final payment (even if it allowed one more access to cash during the 3 years loan period).

Scenario 4 was designed to test if students prefer a student loan with monthly payments and a down payment (down payment of $3,000 and $215 monthly payment for 3 years) or a student loan with higher monthly payments and no down payment ($300 monthly payment for 3 years). We did not have strong predictions about whether students would prefer a loan with a down payment or higher monthly payments.

Scenario 5 was designed to test if students prefer a student loan with a down payment (down payment of $3,000 and $215 monthly payment for 3 years) or a student loan with a final payment ($215 monthly payment with final payment of $3,000). We predicted that students will prefer having a down payment compared with a final payment (again, even if it meant less access to cash during the 3 years loan period).

Participants then answered demographic questions (gender, age, year in school, major, whether they have previously taken out student loans, how much they have taken out in student loans per year) and were debriefed.

#### Power

For within-subject questions comparing how much people would pay on a 10 vs. 15 years loan, we expected a sizeable effect (*d*_*z*_ = 0.5) and would have 99% power to detect it with at least 75 participants. For the between-subjects question where we ask people how much they would pay if the length was 5 years vs. double or triple that length, we conducted a power analysis for a linear contrast (rather than the overall *f* in an ANOVA). For a medium-sized effect (*r* = 0.3), we would have 90% power (Faul et al., 2007).

### Results

**FIGURE 1:**
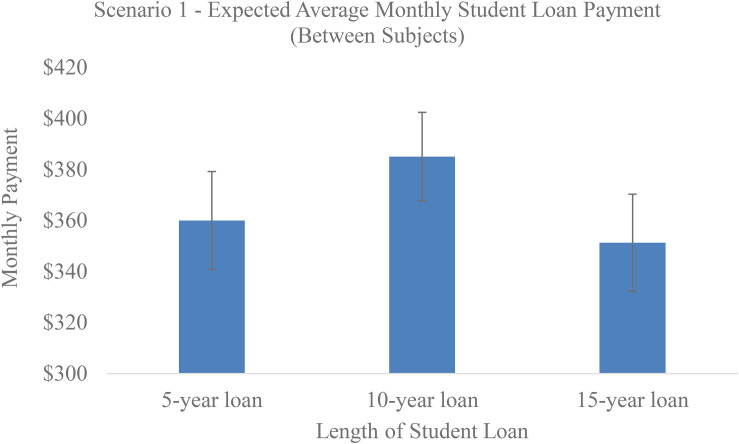
Average monthly payment students were willing to make on loans of different lengths (between-subjects, Experiment 1).

As seen in Figure 1, we conducted a between-subjects ANOVA on the maximum monthly loan payments that students were willing to make for 5, 10, or 15 years loans. There was no significant overall effect of duration [*F*(2, 103) = 0.91, *p* = 0.41] and no significant linear trend (*r* = -0.03)^4^. Participants were willing to pay similar maximum monthly payments for a 5 years loan (*M* = 360.06, SD = 113.48), 10 years loan (*M* = 385.08, SD = 105.56), and 15 years loan (*M* = 351.32, SD = 111.02), indicating duration neglect.

**FIGURE 2:**
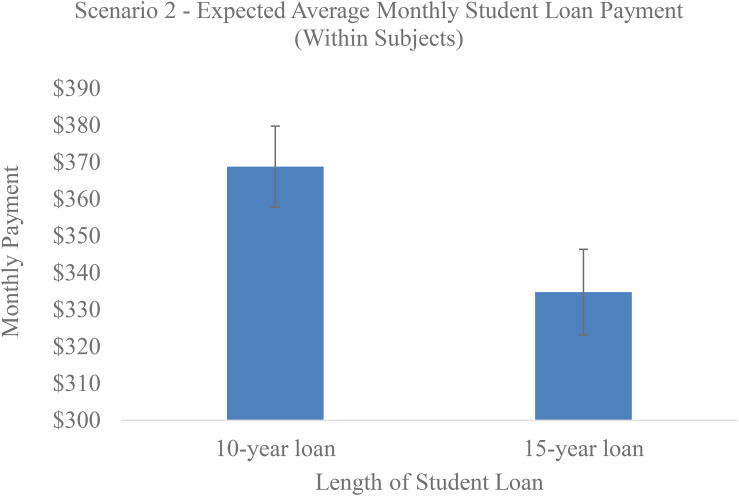
Average monthly payment students were willing to make on loans of 10 and 15 years (within-subjects, Experiment 1).

However, scenario 2 was within-subjects and drew participants’ attention to duration. As seen in Figure 2, when students were asked the maximum monthly loan payment they were willing to pay for a 10 or 15 years loan, they correctly indicated a willingness to pay less per month for a 15 years loan (*M* = 334.76, *SD* = 117.13) compared with a 10 years loan [*M* = 368.78, SD = 110.90; *t*(101) = 4.68, *p* < 0.001], suggesting that our participants were not financially illiterate or innumerate.

On scenario 3, using a slider from 0 to 100, when asked to choose between a student loan with a final payment (0) or a student loan with higher monthly payments (100), participants preferred the loan with higher monthly payments [*M* = 74.31, 95% CI [68.96, 79.65], SD = 28.01, *t*(107) = 27.57, *p* < 0.001, as seen in Table 1]. Only 17.6% of the sample reported responses below 50 (favoring the loan with the final payment), while the majority (80.6%) favored the loan with higher monthly payments.

**TABLE 1:** Students’ choices between loan features (Experiment 1).

Participants choose between loans with:	Mean [with 95% confidence interval]	Participants prefer:
Final payment with lower monthly payment (0) vs. no final payment with higher monthly payment (100)	74.31 [68.96, 79.65]	No final payment
Down payment with lower monthly payment (0) vs. no down payment with higher monthly payment (100)	60.79 [53.99, 67.59]	No down payment
Loan with a large final payment (0) vs. loan with large down payment (100)	62.57 [56.08, 69.07]	No final payment

Similarly, on scenario 4, using a slider from 0 to 100, when asked to choose between a student loan with a down payment (0) or a student loan with higher monthly payments (100), participants preferred the loan with higher monthly payments [*M* = 60.79 [53.99, 67.59], SD = 35.48, *t*(106) = 17.72, *p* < 0.001, as seen in Table 1]. A larger percentage but still a minority (37.4%) chose the loan with the down payment, while the majority (59.8%) chose the loan with higher monthly payments.

When given the option between a student loan with a final payment (0) or down payment (100), people preferred a loan with a down payment [*M* = 62.57 [56.08, 69.07], SD = 34.07, *t*(107) = 19.09, *p* < 0.001, as seen in Table 1]. Only 30.6% chose the loan with the final payment, while the majority (65.7%) chose the loan with the down payment, ignoring the time value of money.

#### Demographics

We examined whether participants’ age, sex, year in school, or whether they had previously taken out student loans moderated the effects above. They did not (all *p*s > 0.23).

#### Summary

Results of Experiment 1 generally pointed to three phenomena: (1) an aversion to final payments, (2) duration neglect, and (3) relatedly, a focus on monthly payments. Regarding the last two points, subjects chose the same payment regardless of whether the loan was for 5 years or for double or triple the length, demonstrating duration neglect. However, if participants’ attention was drawn to duration by having them answer a question about how much they would pay on a 10 vs. 15 years loan (within-subjects), participants did adjust for the length of the loan, confirming that the between-subject effect was due to a failure to attend to duration rather than obvious innumeracy.

Scenarios 3–5 provided preliminary evidence that participants prefer paying at the beginning to paying at the end, possibly due to debt aversion (Prelec and Loewenstein, 1998). Participants also preferred higher monthly payments compared with a payment at the beginning or at the end, suggesting some desire to avoid large lump sum payments.

## Experiment 2

### Materials and Methods

#### Overview

Results from Experiment 1 suggest that in between-subject designs, people are insensitive to the duration of loans and are willing to make similar monthly payments regardless of whether those payments will go on for 5, 10, or 15 years. Participants also seemed to prefer a down payment compared with a final payment, and higher monthly payments to either a final or down payment. Experiment 2 extends the previous results by measuring the extent to which people consider different factors of loans (down payment, final payment, monthly payment, or loan duration). We tested our hypotheses in a between-subjects design by incrementally raising the price of a loan using four different factors to examine how it affects participants’ willingness to accept the loan. Each factor was increased independently until the price of the car was 40% more than retail price. We hypothesized that participants would be much less willing to purchase the car when final payments and monthly payments increased the price. However, we expected participants would be little affected when the price was raised by increasing loan duration. We did not have strong predictions about how sensitive people would be to increasing prices through raising down payments^5^.

#### Participants

Five hundred and twenty-five participants were recruited from Amazon Mechanical Turk for a small fee (*M*_*age*_ = 35, 229 males, 292 females, 4 no response). We conducted a power analysis for a linear contrast with 1 *df* (rather than the overall *f* in an ANOVA). For each factor, we would need 128 participants to discern an effect of size *r* = 0.3 with 90% power.

#### Design and Materials

Participants were told to imagine that they had graduated from college a few years ago and have taken a job across the United States, making approximately $50,000 per year. They are interested in purchasing a new car and are currently at a dealership. On the next screen, participants could browse the dealership’s “inventory” and choose one car among nine car portfolios (which included a picture of the car and features of the car). The nine cars were different makes and models but all had a retail price of around $17,000–$18,000, according to Kelley Blue Book.

On the next screen, participants were offered one of 16 possible loans from the dealership. Car loans were randomly assigned to participants. Car loans started at the base price ($17,000–$18,000) but were incrementally increased by 13% until the price was 40% more than the retail price (four price levels). The total price was increased using four different factors of the loan offer—down payment, monthly payment, final payment, and length of payment—while holding the other factors constant. Using a slider, participants indicated their willingness to buy the car (*0* = *definitely not buy the car, 100* = *definitely buy the car*). We predicted that participants would be highly sensitive to price increases driven by larger final payments and larger monthly payments. In contrast, we expected that participants would be relatively insensitive to price increases driven by longer payment plans.

Participants then responded to demographic information, including their sex, age, religious affiliation, household income, whether they had previously purchased a car, whether they manage the finances in their household, and education. With these intervening questions, we hoped this would be enough time to “reset” participants, so that we could attempt to get a second measurement, telling participants that the dealership would like to make a second and final offer. If the participant’s first answer was less than 100 (as all but 18 responses were), they were told, “Your answer of ___ is lower than the dealership likes to see to close a deal. The dealership would like to make a second and FINAL offer (there will be no third offer).” The participant then received a car loan offer that was the same total amount as the original loan, but whose costs were structured differently. For instance, if the first loan was increased through monthly payments, they could receive an offer that was increased through duration, down payments, or final payments. However, if the first offer contained a down payment or final payment, the second offer would *not* include a final payment or down payment, respectively, because participants would likely notice that the lump sum had just moved from the beginning to the end, or vice versa.

At the end of the experiment, participants were asked: “Please try to recall the terms of the FIRST (LAST) loan offer that they gave you…If the offer did not contain one of these features, please leave it blank or put 0.” There were four entries possible for down payment, final payment, monthly payment, and duration. We predicted that participants would have been less likely to encode duration of the loan offer and so would have trouble accurately recalling it. We also attempted to see whether an incentive (triple the payment) would improve participants’ accuracy. Half of the participants were told “As a reward for accuracy, if you get within 10% of the correct answer for all loan terms, we will triple the payment to you.”

### Results

#### First Offer

To test for differences in participants’ willingness to purchase the cars based on how the price was increased, we ran an ANOVA using condition (down payment, final payment, duration, monthly payment) and level (price 1, price 2, price 3, price 4) as factors. We ran planned linear trend contrasts with equal spacing (−3 −1 1 3) for each condition.

Means for each factor may be seen in Table 2. As predicted, when running the linear contrast, participants were significantly less willing to purchase the car when the price was increased through higher monthly payments [*t*(1, 125) = −2.25, effect size *r* = −0.19, *p* = 0.03] and bigger final payments [*t*(1, 129) = −2.60, *r* = −0.23, *p* = 0.01]. Participants were not significantly affected by increasing down payments [*t*(1, 126) = −1.38, *r* = −0.12, *p* = 0.17] or duration [*t*(1, 129) = −1.26, *r* = −0.12, *p* = 0.19].

**TABLE 2:** Willingness to purchase a car as a function of price increases due to larger down payment, final payment, monthly payment, or duration of payment (Experiment 2, loan offer 1).

Monthly payment	Mean	SD	Final payment	Mean	SD
510	*M* = 40.10	(31.65)	$2,600	*M* = 44.35	(34.63)
580	*M* = 40.50	(30.75)	$5,000	*M* = 37.25	(30.00)
650	*M* = 35.36	(37.35)	$7,400	*M* = 29.12	(24.51)
710	*M* = 23.67	(30.92)	$9,800	*M* = 24.88	(32.05)

**Down payment**	**Mean**	**SD**	**Length of payment**	**Mean**	**SD**

2,600	*M* = 48.84	(33.10)	42 months	*M* = 43.29	(31.52)
5,000	*M* = 31.52	(31.38)	47 months	*M* = 41.52	(28.05)
7,400	*M* = 44.07	(32.56)	53 months	*M* = 45.57	(29.69)
9,800	*M* = 33.10	(28.14)	58 months	*M* = 32.26	(28.84)

#### Second Offer

Responses to the second offer reflected preferences exhibited for the first offer. At a given price level, participants would rather pay for a longer time than face a higher final payment [average difference between loans made more expensive by increasing duration vs. increasing final payment = 13.56, 95% CI [7.85, 19.28], *t*(104) = 4.71, *p* = 0.001]. They would rather pay for a longer time than have a higher monthly payment [average difference = 11.74, 95% CI [6.16, 17.32], *t*(83) = 4.18, *p* = 0.001]. However, in contrast to Experiment 1’s findings, they would rather have a higher down payment than a higher monthly payment [average difference = 5.85, 95% CI [1.08, 10.62], *t*(85) = 2.44, *p* = 0.017]. In trade-offs between monthly and final payments or durations and down payments, participants were either indifferent or showed only slight preferences [average difference = 2.37, 95% CI [-3.14, 7.88], *t*(110) = 0.85, *p* = 0.40; average difference = 3.58, 95% CI [−0.69, 7.85], *t*(120) = 1.66, *p* = 0.100, respectively].

#### Memory for Four Factors

**FIGURE 3:**
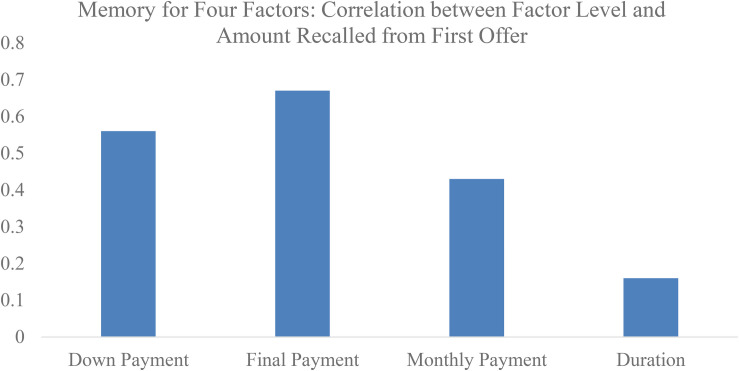
Memory for features of the first loan offer (Experiment 2).

Participants were asked to recall the terms of the loans that they were offered. We predicted that participants would be more accurate in remembering monthly payment and final payment terms, and so, the correlation between level (price 1, price 2, price 3, price 4) and the recalled amounts would be significant. However, we predicted that people would be less attentive to duration, and so there would be no significant correlation between level and participants’ estimates for plan length. We report correlations for the first and second offer. As seen in Figure 3, in the down payment condition, level was positively correlated with the amount recalled for down payments (*r*_*first*_ = 0.56, *p* < 0.001; *r*_*second*_ = 0.52, *p* < 0.001). In the final payment condition, level was positively correlated with the amount recalled for final payments (*r*_*first*_ = 0.67, *p* < 0.001; *r*_*second*_ = 0.63, *p* < 0.001). Similarly, in the monthly payment condition, level was positively correlated with amount recalled for monthly payments (*r*_*first*_ = 0.43, *p* < 0.001; *r*_*second*_ = 0.18, *p* = 0.03). However, in the duration condition, level was not significantly correlated with the duration recalled (*r*_*first*_ = 0.16, *p* = 0.101) for the first offer. Level was correlated with the duration recalled for the second offer (*r*_*second*_ = 0.25, *p* = 0.001), which was likely given that the offer had just been presented on the previous screen. The relatively low accuracy for duration is consistent with participants not paying much attention to this factor when considering the loans. The relatively high accuracy for down payments is consistent with participants paying attention to this factor, but not caring much about it. Accuracy did not differ between the incentive and no-incentive conditions (all *p*s > 0.12)^6^.

#### Experience

Whether participants had previously bought a car, had an auto loan, or managed the finances in their households had little effect on any of the variables, with a few exceptions. Those who had experience with a car payment plan (compared with those who had no experience with a car payment plan) were more likely to want low monthly payments and were willing to trade-off higher down payments and longer durations to accomplish this (in comparison between the first and second loan offer, *p*s ≤ 0.026). Those who did not manage the household finances had better memories for the final payment on the second car (*p* = 0.001). In terms of education, this did moderate the duration neglect phenomenon for the first offer (*b* = −9.12, *t* = −3.72, *p* = 0.001, partial *r* = −0.32). If respondents had less than a bachelor’s degree, increasing loan amounts by increasing duration actually made the plans marginally more desirable (partial *r* = 0.17, *p* = 0.060), while doing so made it less desirable for those with at least a bachelor’s degree (partial *r* = −0.29, *p* = 0.001). It is possible that duration neglect is more common among those without a 4 years degree. However, we hesitate to make much of this because effects were found among college students in Experiment 1, and education did not moderate effects in Experiment 3 (education was not measured in Experiment 4).

#### Four-Factor Summary

In sum, we increased the price of cars through four factors (down payment, final payment, monthly payment, or loan duration) until the car was 40% higher than its retail price. As predicted, we found that participants were much less willing to purchase cars when the cost of loans increased due to larger final payments or monthly payments. The effects of down payment and duration were not significant. Memory data suggested that down payment offers were attended to but not given much weight in the decision, whereas duration was not well attended to nor given much weight in the choice.

## Experiment 3

### Materials and Methods

#### Overview

Experiment 2 demonstrated that, similar to pain, people tend to neglect duration and overweight the end of the loans. Experiment 3 extends the previous results and tests another counterintuitive prediction of the peak-end rule. That is, because people weigh the ending so heavily and ignore duration, a pain sequence which is lengthened by *adding* a somewhat less painful experience at the end is sometimes preferred to a shorter sequence without this additional pain. Even though it involves taking more “objective” pain, adding a somewhat less painful ending makes the sequence seem better (Kahneman et al., 1993; Redelmeier and Kahneman, 1996; Diener et al., 2001).

In Experiment 3, we examine whether a parallel effect holds for payment sequences. The hypothesis is that participants’ preference for a loan will actually *increase*, if less “painful,” less expensive monthly payments are added on to the end of the payment plan. Though it is a longer and more expensive loan, participants should prefer it because of its “less painful” ending. In addition, we measured participants’ intuitions about the aversiveness of unpleasant sequences of physical pain (conceptually replicating Varey and Kahneman, 1992).

We ran three preliminary experiments investigating participants’ preference for longer, more expensive loans with less “painful” endings to estimate an effect size (*d* = 0.08) and appropriate sample size. We have provided a detailed description of the three experiments and results in Supplementary Materials.

Note also that whereas the peak-end rule predicts people will prefer more expensive loans with less “painful” endings tacked on to them, other alternative explanations do not readily do so. For example, it is possible that people in Experiments 1 and 2 wanted to pay up front and avoid heavy final payments either as a commitment device or to avoid the possibility that they will not have the money for the final payment. Though the consumer finance industry is built on the opposite premise (that people are overoptimistic about the future) and though much psychology research also suggests people are overly optimistic, this cannot be ruled out as an explanation for findings in Experiments 1 and 2 that people dislike expensive end payments. However, neither of these alternative explanations can readily make the prediction for Experiments 3 and 4 that people will prefer longer, more expensive loans because we have tacked “less painful” payments on to the end of them.

#### Participants

Two thousand six hundred and twenty-eight participants were recruited on Amazon Mechanical Turk for a small payment. After excluding duplicate IP addresses and IP addresses not from the United States, 2,384 participants remained. We conducted a meta-analysis of the three experiments we previously ran and found that the average Cohen’s *d* of our last three experiments was *d* = 0.08. We calculated that with a sample of 2,500 and an expected effect size of *d* = 0.08, for an independent *t* test, we would have approximately 65% power.

#### Design and Materials

##### Car loan set 1

Participants were told to imagine that they had just graduated from college, had a $60,000 income, and had $10,000 in the bank. They were told that they would be evaluating a series of cars and payment plans. There were two conditions in the experiment—participants in the “base” condition received six loan offers (for example, $500 per month for 44 months) and participants in the “base + 12 month” condition received the same six loan offers but an additional 12 months of smaller payments were added at the end (for example, $500 for 44 months followed by 12 additional monthly payments of $150 per month). (As a cover story for the smaller amount added for the last 12 months, participants were told that the financing would come from a variety of sources—some from the manufacturer, some from a bank, and so on. Because the sources were loaning different amounts for different lengths of time, the monthly payment amount might change over the course of the loan, though the loans would all be bundled together into one convenient payment). Base payment plans ranged from 32 to 60 months. We predicted that participants in the “base + 12 month” condition would indicate a higher willingness to take the loan, compared with participants in the (shorter and less expensive) “base” condition^7^.

#### Individual Difference/Experience Variables

Participants were then told that the experimenters were interested in people’s intuitions about uncomfortable experiences. The instructions were taken from Varey and Kahneman (1992), and participants were told that people were paid to participate in a series of uncomfortable experiences. Every 5 min, they made a rating (from 0 to 10) of the discomfort they were feeling at that moment. Participants were told that their task was to provide an overall evaluation of the discomfort for each pain sequence using a scale from 0 to 100 (0 = not bad at all, 100 = extremely bad) (see Varey and Kahneman, 1992 for complete instructions^8^).

For between-subject questions, participants were assigned to randomly see five “base” (e.g., 2 5 4 4 7 9 6 6) or “base + less painful end” pain scenarios (e.g., 2 5 4 4 7 9 6 6 2). Participants also rated 18 pain scenarios (within-subject) where sequences contained “base” (e.g., 4 7 8 9 8 7 6), “base + less painful end” (e.g., 4 7 8 9 8 7 6 1), or “base + more painful end” numbers (4 7 8 9 8 7 6 9).

We also tested whether our predicted effects would be moderated by individual difference variables related to the pain preferences above and to participants’ “sensitivity to spending.” We created a “sensitivity to spending” index that assessed whether people find it painful to spend money (e.g., “I find it painful to spend money”; “The thought of spending makes me anxious”), and also measured whether people are tightwads or spendthrifts (from Rick et al., 2007). In addition, we explored whether education, having previously purchased a car, or having taken out a car loan moderated our main effects.

After filling out demographic questions, participants were asked to rate another set of cars and payment plans.

##### Car loan set 2

Finally, participants rated the set of payment plans that they had *not* previously received but for more expensive cars; for example, if they rated the six “base” payment plans in the beginning of the survey, they rated six “base + 12 month” payment plans later in the survey (or vice versa). By including demographic questions, the sensitivity to spending scale, and pain questions between the two differing sets of payment plans, we attempted to “reset” people so that they would look at the new payment plans, unaffected by their responses to the first set of payment plans. We hoped that people would *not* be influenced by the first set of questions or make any implicit comparisons between the first and second set of payment plans, so that we could get two independent responses from participants^9^.

### Results

#### Car Loan Scenarios

**FIGURE 4:**
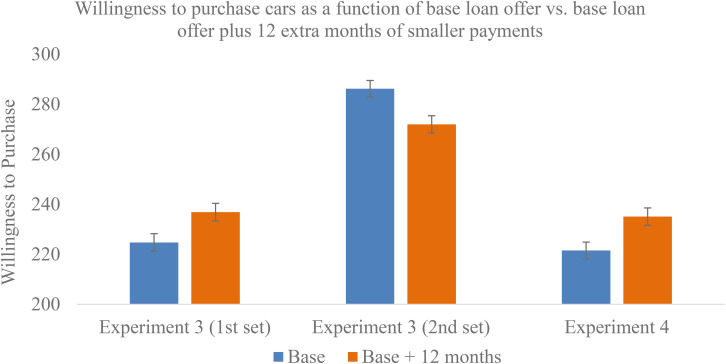
Willingness to take a loan, depending on whether it had 12 extra months of “less painful” payments added on to the end (Experiments 3 and 4).

As predicted and as seen in Figure 4, for the first set of payment plan questions, participants preferred the more expensive “base + 12 month” plans (*M* = 236.87, *SD* = 121.20) to “base” plans [*M* = 224.74, SD = 122.74; *t*(2,382) = 2.43, Cohen’s *d* = 0.10 [0.02, 0.18], *p* = 0.02]. However, the reverse effect occurred on the second set of questions, where people preferred the “base” plans (*M* = 286.19, *SD* = 115.15) to the “base + 12 month” plans [*M* = 271.98, SD = 118.21; *t*(2,383) = −2.97, Cohen’s *d* = -0.12 [−0.20, −0.04], *p* = 0.003], suggesting that we did *not* successfully reset people. These questions were acting like the second measure of a within-subjects design rather than a fresh between-subjects measure (see the within- vs. between-subjects distinction in Experiment 1). The reversal on the second set of questions could derive from participants contrasting the payment plans in the second set of questions with those in the first set of questions, creating a within-subjects standard of comparison. Alternatively, it could be that the first group of questions “set” our participants in terms of their responses (willingness to buy) and this “set” carried over into the second group of questions. There was enough ambiguity that we decided to run Experiment 4.

#### Individual Differences/Experience

We tested whether participants’ willingness to buy the car (on the first set of questions) was predicted by the sensitivity to spending scale, pain questions, “base” vs. “base + 12 month” condition, and the interaction of these variables. None of the two- or three-way interactions moderated the effects.

We also tested whether previously purchasing a car, previously having a car loan, or participants’ education moderated the main effect. For between-subject payment plan questions, the interaction between previously purchasing a car (1 = Yes, 0 = No) and “base” vs. “base + 12 month” condition (0 = base, 1 = base + 12 months) was marginally significant in predicting participants’ willingness to buy the car [*b* = 21.01, *t*(2,379) = 1.83, partial *r* = -0.04, *p* = 0.07]. Here, previous experience with buying a car led people to be more likely to fall into the duration-neglect trap. Participants who had previously purchased a car indicated *more* willingness to buy in the “base + 12 month” condition (*M* = 228.48, *SD* = 122.99) compared with the “base” condition (*M* = 210.78, *SD* = 121.20) [*t*(1,807) = 3.08, partial *r* = 0.06, *p* = 0.002], whereas people who had not previously purchased a car indicated similar willingness in both conditions (base *M* = 267.99, base *SD* = 115.48, base + 12 months *M* = 264.44, base + 12 months *SD* = 111.11) [*t*(573) = −0.35, partial *r* = −0.01, *p* = 0.73]. Among those who had purchased a car, the preference for the base + 12 month condition over the base condition was similar in size both among those who bought the car on a payment plan [*t*(1,067) = 2.56, *p* = 0.011, *d* = 0.16] and among those who bought the car with cash [*t*(744) = 1.950, *p* = 0.052, *d* = 0.14]. (Cash vs. payment plan × base vs. base + 12 interaction *F*(1,811) = 0.02, *p* = 0.89). The “base vs. base + 12 month” × education interaction was also not significant (*t* = −0.08, *p* = 0.94).

#### Summary

Results from Experiment 3 suggested that in a between-subject design, people prefer loans that are longer and more expensive if smaller, “less painful” payments are added to the end of a loan. Experience, education, or individual differences did little to modify this effect.

However, in this experiment, we attempted to use two sets of between-subject measures for the loan questions and found a reversed effect for the second set of questions where people preferred the “base” loans to the “base + 12 month” loans, perhaps because participants anchored on their first answer or perhaps because we had unintentionally created a within-subjects standard of comparison. To provide greater clarity on the basic effect and to examine robustness, we ran Experiment 4 to examine whether the effect of preferring “base + 12 month” loans to “base” loans holds under various conditions.

## Experiment 4

### Materials and Methods

#### Overview

We designed Experiment 4 to replicate the effect in Experiment 3 and to also test whether it holds under a range of conditions. Specifically, we varied the income participants were told to imagine having, when they would be purchasing the car (now or in the future), and the price of the car. We predicted that the main effect of preferring longer and costlier car loans (with less expensive payments tacked onto the end) would hold across these different factors.

We randomly assigned participants to the “base” or “base + 12 month” condition (between-subjects). We assigned participants to evaluate one set of payment plans rather than two different sets (as in Experiment 3), thereby avoiding the problem of creating a within-subjects design with a second set.

#### Participants

Two thousand six hundred and ninety-four participants were recruited on Amazon Mechanical Turk for a small payment. After excluding duplicate IP addresses and IP addresses not from the United States, there were 2,621 participants. To determine sample size, we used a similar calculation from Experiment 3. We conducted a meta-analysis of the last four experiments and found that Cohen’s *d* averaged 0.09. We calculated that with a sample of 2,500 and an expected effect size of *d* = 0.09, we would have approximately 70% power to find a one-tailed effect.

#### Design and Materials

Participants were told they would be reading over scenarios and evaluating cars and payment plans. We included four different factors that could vary among participants: condition (participants received six car scenarios that were either framed in terms of “base” or “base + 12 month” payments), income (participants were told to imagine they were making $60,000 per year with $10,000 in the bank or making $90,000 per year), time (purchase the car now or 15 years in the future), and car price (MSRP of $22,000 or $25,000). An example of a loan offer in the “base” condition might be a $458 monthly payment for 48 months; an offer in the “base + 12 month” condition might be a $458 monthly payment for 48 months, followed by 12 months of a $150 monthly payment.

At the end of the survey, participants were asked three questions about car prices and income given in the survey instructions. All responses were on sliders from 0 to 100. For the cars, participants were asked whether the cars shown were “very different from the cars I would actually purchase in real life” to “very similar to the cars I would actually purchase in real life.” They were also asked whether the cars shown were “much less expensive than the ones I would actually purchase” to “much more expensive than the ones I would actually purchase.” For income, participants were asked, “In real life, if I had a $60,000 salary with $10,000 in the bank [had a $90,000 salary], I would feel “much poorer than I actually do in real life” to “much richer than I actually do in real life.” (We only thought to add these questions after data from about 800 participants were collected, so data are missing for them)^10^.

#### Individual Differences/Experience

We tested whether the effect of payment plan on willingness to buy the car would be moderated by participants’ previously purchasing a car or previously having a car loan.

### Results

#### Overall Effect—Between-Subject Debt Scenarios

We tested whether participants preferred the “base + additional 12 months” compared with the “base” condition using an independent samples *t-*test. As seen in Figure 4, the expected difference emerged as participants overall preferred the more expensive “base + additional 12 months” plans to the “base” plans (*M* = 235.09, *SD* = 126.41 vs. *M* = 221.55, *SD* = 120.28) [*t*(2,619) = 2.81, *p* = 0.005, Cohen’s *d* = 0.11 [0.03, 0.19]]. To reiterate, the “base” payments were the same for both plans; the “base + additional 12 months” plans merely had an additional 12 months of smaller payments tacked on to them, and it was these “base + additional 12 months” plans that participants preferred in our between-subjects design. This finding replicates the effect from Experiment 3’s first set of questions.

##### Income ($60,000 vs. $90,000)

We examined whether different levels of imagined income affected participants’ willingness to purchase the cars. Using a 2 × 2 ANOVA, there was a significant main effect of income [*F*(1, 2,617) = 24.86, *p* < 0.0001]; participants were more willing to purchase the cars with an imagined income of $90,000 (*M* = 240.31, *SD* = 123.17) compared with an income of $60,000 (*M* = 216.26, SD = 122.86). Though the difference between “base” and “base + 12 months” was bigger when participants imagined they had the smaller income (difference = 16) rather than the bigger income (difference = 10), the income × condition interaction was not significant [*F*(1, 2,617) = 0.47, *p* = 0.50].

##### Time (now vs. 15 years)

Using a 2 × 2 ANOVA, we tested whether imagining buying the cars now or 15 years in the future affected participants’ willingness to purchase the cars. There was a significant main effect of time [*F*(1, 2,617) = 4.13, *p* = 0.04], with participants preferring to purchase the cars if they thought the time was the present (*M* = 233.32, *SD* = 123.25) vs. 15 years from now (*M* = 223.40, *SD* = 123.76). Though the difference between “base” and “base + 12 months” was bigger when participants thought the time was now (difference = 21) rather than 15 years from now (difference = 5), the time × condition interaction was not significant [*F*(1, 2,617) = 2.45, partial *r* = -0.03, *p* = 0.12].

##### Car price ($22,000 vs. $25,000)

There was a significant main effect of car price [*F*(1, 2,617) = 7.64, *p* = 0.006], with participants being more willing to purchase the $22,000 cars (*M* = 234.92, *SD* = 121.96) compared with the $25,000 cars (*M* = 221.55, *SD* = 124.93). There was also a significant car price × condition interaction [*F*(1, 2,617) = 4.70, partial *r* = -0.04, *p* = 0.03]. Specifically, there was a significant difference for the $22,000 cars between the “base” (*M* = 222.94, SD = 117.79) and “base + 12 month” condition (*M* = 246.72, *SD* = 124.90) [simple effect *t* = 3.54, *p* < 0.001, *d* = 0.19]. However, participants who saw the $25,000 cars reported no significant difference between the “base” (*M* = 220.07, *SD* = 122.94) and “base + 12 month” cars (*M* = 222.99, *SD* = 126.92) [simple effect *t* = 0.42, *p* = 0.67, *d* = 0.02].

The non-significant result found for the $25,000 cars may be due in part to the higher price point. As will be recalled, participants were asked, on a scale from 0 to 100, whether the cars shown to them were much less expensive than the ones they would actually purchase to much more expensive than the ones they would actually purchase. Participants who rated the $25,000 cars were more likely (*M* = 71.62, *SD* = 22.19) to say that the cars were more expensive than what they would actually purchase, as compared with the $22,000 condition (*M* = 65.91, SD = 21.76) [*t*(1,881) = 5.64, *p* = 0.001].

It is possible that the effects observed in this paper are peculiar to cars having a relatively low price point, and effects would not hold at higher price points for these participants. It is also possible that we stumbled into a “sweet spot” where effects would be observed because the price point was neither too high nor too low for our participants. This cannot be ruled out—and likely there is a “sweet spot” for all people; that is, (a) prices cannot be so high that everything becomes “fantasy land” and payment plan has no effect and (b) prices cannot be so low that participants do not even feel their choices are significant or care what the payment plan looks like. Our guess is that the key to the effect holding is that the imagined circumstance has to feel real and as if one would care about it.

Our interpretation is essentially that anything that leads the problem to feel less “real” to participants—making it about a purchase 15 years in the future, having participants imagine a “too high” salary, making the cars more expensive than cars they would actually buy—diminishes the effect. To illustrate, we can compare the effect of condition when the purchase was near, salary was lower, and cars were less expensive (maximal “realness”) with the effect of condition when the purchase was far, salary was higher, and cars were more expensive (minimal “realness”). Among the 334 participants run under “maximal realness,” the difference between “base” vs. “base + 12 months” was 210 vs. 248 [*t*(332) = 2.84, *d* = 0.31]; among the 322 participants run under minimal realness, the difference was 220 vs. 225 [*t*(314) = 0.37, *d* = 0.04]. If choices have to feel real to participants, it will be important to have participants looking at cars they might actually buy. The appropriate price range and style of car will, of course, depend on the population studied.

#### Individual Differences/Experience

There was a main effect, such that people who had previously purchased a car [*b* = -42.83, *t*(1,882) = −3.71, *p* < 0.001] or had taken out a car loan [*b* = -20.73, *t*(1,882) = −2.52, *p* = 0.01] indicated a lower willingness to purchase the cars. However, there was no significant interaction between condition (base vs. base + 12 months) and previously purchasing a car (*p* = 0.57) or between condition and previously having a car loan (*p* = 0.35).

#### Summary

Experiment 4 replicated results from the first set of questions in Experiment 3, suggesting that there is a small effect such that participants preferred more expensive car loans that ended with “less painful” payments added on. There was a weak tendency for the effect to be more pronounced when the imagined scenario was more “immediate” or “realistic” (occurring now and assuming a salary and price range more appropriate for the population).

## Conclusion

In the four experiments, we investigated the hedonics of debt and found that when people prospectively evaluate a loan package, they overweight the end of the experience and show strong duration neglect. Student loan packages could be doubled or tripled in length and prices of cars could be increased 40% by stretching out the duration of the loan without significantly changing people’s preferences. Further, people could be induced to *prefer* more expensive loans, liking loans better (in a between-subjects design) if additional smaller, less painful payments were tacked on to the end of a payment sequence.

Unlike with pain, however, participants also seem to place great weight on average, non-peak moments—in this case, focusing on the monthly payment, often to the exclusion of other relevant factors (such as down payment or length of the contract). This is the reason car salespeople often begin negotiations by asking customers, “How much do you want your monthly payment to be?” (Reed, 2019). Once the monthly payment seems affordable to customers, profits for the dealer can increase as salespeople add in up-front “fees,” increase down payments, or stretch out the length of the loan—features that customers are relatively impervious to. As the memory data suggest, people do not much attend to duration information; they attend to down payment information but do not weight it very much.

The hedonics of debt seem to operate heuristically in that even experience and expertise (having bought a car or taken out loans, managing family finances, or coming from a high-income family) do little to help people avoid their blind spots. Additionally, the individual difference variables we examined in Experiment 3 did nothing to prevent people from getting “suckered” into actually preferring more expensive loans. A summary of the main findings across studies may be seen in Table 3.

**TABLE 3:** Summary of experiments.

Experiment	Experimental questions	Conclusion
Experiment 1	Sensitivity to loan duration; preferences among down payments, final payments, or no final/down payments	Participants insensitive to loan duration (effect does not hold for within-subject questions); prefer payment at the beginning
Experiment 2	Willingness to purchase car as affected by down payments, final payments, monthly payments, or duration	Willingness to purchase was much less when cost increased from larger final payment or monthly payments; increasing down payment and duration were not significant; memory about loan duration was poor
Experiment 3	Willingness to purchase as affected by smaller, less “painful” monthly payments tacked onto loan (making the price more expensive)	Willingness to purchase greater with 12 months of payments tacked onto the end of loan
Experiment 4	Moderation of main effect of Experiment 3 (preferring longer but “less painful” loan endings) by hypothetical income, the price of the car, or timing of purchase (now vs. later)	Experiment 3 results replicated; weak tendency for larger effect when the imagined scenario occurred now and at a salary and price range more appropriate for the respondent population

### Power, Effects, and Boundaries

One should, of course, be cautious about any effect involving duration neglect, because claims of “neglect” ride on finding a null effect of duration. Thus, the issue of statistical power must be considered. If one imagines (in Experiments 1 and 2) that in a between-subjects design, participants would differ in their willingness to pay if their student loans were tripled or their car prices were increased by 40% and guesses that such effects would be in the “medium” range, Experiments 1 and 2 would be decently powered. But, of course, intuitions about effect size can differ and so it will be useful to run extremely high-powered studies to see how far claims of duration neglect might be pushed.

To be clear, we do believe there are boundary conditions. Students might not differ in the amounts they are willing to pay monthly over the course of 5 vs. 15 years. But we think it highly likely they would differ if duration lengths were 5 vs. 50 years. Similarly, we think it highly unlikely anyone would end up paying $75,000 for a Ford Fiesta, because they paid insufficient attention to a contract that ran four times longer than normal or got suckered by small payments that were added to the end. Our suspicion is that the amount one can pile on is stimuli- and context-specific (Murphy, 1995; Hershfield and Roese, 2015; Hershfield et al., 2015). In other research, we have found that between-subjects participants would (hypothetically) be willing to make the same monthly payments if their mortgage was made 50% longer (from 20 to 30 years). However, it would be helpful to develop a general theory of factors and contexts that make duration more or less salient and more or less important. It would be helpful also to take studies out of the lab and into the world. This is, of course, the most important test of external validity, and whereas this paper does not have such tests, the results are consonant with a number of real world phenomena suggesting that—as long as monthly payments are affordable—prices can be increased because consumers are willing to make payments for a long, long time.

### Techniques Outside the Lab

People’s insensitivity to the length of the loan is likely one reason automobile loans have seen such large increases in duration. As of 1972, only 1% of dealer-financed auto loans lasted for over 3 years (Seaton and Vogel, 1980). By 2020, the *average* car loan went for almost 6 years (Arnold, 2019; Experian, 2020; Montoya, 2020). Interestingly, in the 1920s and 1930s, finance rates were *lower* on longer loans [because loans often involved a flat charge (Shay, 1963; Juster and Shay, 1964)]. Now, rates are typically higher on longer loans because they involve more risk for the lender, but it is also likely that increasing the finance costs on longer-term loans could be accommodated because consumers did not mind or even preferred longer loans (even mattresses now come with 6-year payment plans)^11^.

Down payments also seem not to matter as much as end payments or monthly payments. Perhaps this is *one* reason people in the United States are so accommodating to the many fees that accompany mortgages (and car loans) as up-front costs (Akerlof and Shiller, 2015). Further, in one dataset running from 2001 to 2011 with borrowers who had good credit (“prime” borrowers), about 12% of respondents paid “points” up front (1 point = 1% of the loan) to lower their interest rate—a move that could be advantageous, but usually was not and cost the average borrower about $700 (Agarwal et al., 2017; Chang and Yavas, 2020). A question naturally arises, however: if people do not mind large up-front payments, then why are “no money down” advertisements so prevalent? Although heuristics matter in financial decision-making, they are not the only thing that matters. In many cases, people do not have the cash to make big down payments due to liquidity constraints (Walker and Sauter, 1974; Seaton and Vogel, 1980; Attanasio et al., 2008; Adams et al., 2009). “No money down” offers are often the only way many people can be enticed into financing. Given that people also show duration neglect and focus on whether the monthly payment is affordable, sellers have other ways of making financing profitable for themselves and prospectively palatable for customers, once these customers have decided to buy on credit.

Issues can be raised about this research concerning the sample and the type of decisions we have had people make in these experiments. First, samples were not probability samples (a statement true of most studies). Second, we presented our loan offers in a stripped-down format, giving down payments, monthly payments, and durations. We did not, for example, present APRs, which are standardized rates that make it easier to compare loans. Having APRs would presumably allow people to make better decisions—assuming they (a) paid attention to APR (Ranyard et al., 2006), and (b) perhaps more importantly, actually comparison-shopped for loans. Related to (b), some effects—such as duration neglect and the preference for loans with extra, “less painful” monthly payments tacked onto the end—held only in between-subject designs. Does this imply that such an effect would not occur in the real world where people can shop around for, say, car loans? Perhaps. But only about half of car shoppers in the United States and The Netherlands visit more than one dealership, and only 50% of Americans actually shop around for their auto loans (Morton et al., 2011; Moraga-Gonzalez et al., 2015; Consumer Financial Protection Bureau [CFPB], 2016). For mortgages, the data are similar. Only about half of mortgage borrowers shop around, according to a 2014 study by the Consumer Financial Protection Bureau [CFPB] (2015). A mortgage is, by far, most people’s largest debt (if they own a home). We suspect that even fewer than 50% of the population shops around for mattress loans, jewelry financing, deals on “rent-to-own” appliances, “only on TV” special offers, or other consumer purchases. Thus, the between-subjects nature of the experimental design was probably true to many people’s lived experiences, even if it was not true for all people’s lived experiences^12^.

In all four experiments, decisions were not “real” in the sense that actual customers were parting with actual cash in a real purchase situation with an actual salesperson using a “four-square” worksheet (Slone, 2007). Most of these factors probably make decisions more likely to be scrutinized—though the presence of a salesperson and all the theater that goes with the negotiations make it much more likely people will accept a bad deal. Nevertheless, it remains to be seen whether people making actual decisions show the same effects as those found here. Only empirical research can provide the answer. However, given that some of what we have found (a focus on monthly payments, a neglect of duration) seems so consonant with many sales techniques employed in the world, we suspect that such effects are frequently used outside the lab to make debt more palatable. Understanding the hedonics and the principles that make debt seem so easy to take on (though not necessarily so easy to live with) is likely one part of explaining how Americans have written $14 trillion worth of IOUs on their future.

## Data Availability Statement

The datasets generated for this study are available on request to the corresponding author.

## Ethics Statement

The studies involving human participants were reviewed and approved by the University of Illinois Institutional Review Board. The patients/participants provided their written informed consent to participate in this study.

## Author Contributions

FS, DC, RL, and JP contributed to producing the research and the write-up. All authors contributed to the article and approved the submitted version.

## Conflict of Interest

The authors declare that the research was conducted in the absence of any commercial or financial relationships that could be construed as a potential conflict of interest.

## Footnotes

1 Most peak-end studies focus on retrospective judgments. However, there have been some studies illustrating the applicability of peak-end to prospective judgments (e.g., Varey and Kahneman, 1992; Diener et al., 2001). The study by Diener et al. (2001), for example, found what they called the “James Dean effect”: participants prospectively rated a wonderful life that ended suddenly as better than one with the addition of mildly pleasant years. Participants will also make prospective judgments based on their retrospective recall: in the study of Kahneman et al. (1993), participants chose to repeat a sequence involving 3 min of pain (2 min of intense pain plus 1 min of moderate pain) over a sequence with only 2 min of intense pain. Memory effects are frequently (though not exclusively) offered to explain peak-end findings, as the greater recall of peak and final moments influences participants’ judgments. In the present paper, we are examining prospective judgments and argue that attention is a key explanatory variable: participants are just likely to focus on other aspect of the loan, paying little attention to duration. “Memory” explanations and “attention” explanations are not as far apart as they seem, however, because memory necessarily involves selective attention to some moments of past experiences or to some aspects of it rather than others.

2 Langer et al. (2005) examined whether peak-end applied to a sequence of payments. But in their two studies, payments were payments *to* participants along with penalties. In experiment 1, they found no support for peak-end. In experiment 2, they did. They concluded that “such effects only show up if we link the payments to performance in strenuous tasks that distract the participants. If payments are simply presented and no distraction is provided, most subjects make normatively appropriate choices” (p. 157).

3 The original and most memorable studies of the peak-end rule involved physical pain. And anticipations of physical pain, expectations of loss, and high prices may all involve some common neurological circuitry (activation of the insula) (Knutson et al., 2007; see also Zellermayer, 1996; Durkin, 2007; Xu et al., 2015 for other parallels between the pain of paying and physical pain). However, the point is not that physical pain and the pain of paying are interchangeable. The point is that what holds so clearly with the irredeemable and unalloyed negative experience of physical pain also seems to hold when it comes to summary evaluations of hedonic experience more generally. In this case, the hedonic experience is the “pain of paying.” Clearly, hedonic evaluations of physical pain and the pain of paying *should* be different in some ways. For one thing, physical pain can range from mildly annoying to excruciating, but once it is over, it is over. This is not the case with money, where the total amount spent has consequences for an individual’s stock of wealth going forward. Thus, $1,000 spent on one thing means I have $1,000 less to spend on something else in the future. *Normatively* then people should be highly sensitive to duration of debt payments (because of its influence on total cost). The experiments of this paper suggest that *descriptively* this is not the case. It is possible that drawing participants’ attention to total costs could lessen the duration neglect and peak-end effects shown here. However, at least for major purchases like cars and houses and perhaps other purchases as well, total cost is not given to the customer until after a lengthy purchasing, negotiating, or application process has been completed and the deal is “in the bag.”

4 Effect sizes are given in this paper, though we think it unwise to gauge them solely by “conventional” criteria of what is small, medium, or large. Instead, we think it better to evaluate effects against expectations and theoretical relevance. Regarding expectations, one assumes a person would notice if a loan was doubled or tripled in amount (Experiment 1) or if one is asked to pay $25,000 for an $18,000 car (Experiment 2). In fact, this expectation is confirmed if prices are increased through raising either the final or monthly payments. However, this expectation is not confirmed—or the trend is at least not statistically significant—if price is increased by lengthening a loan’s duration. Regarding theoretical relevance, one of the striking aspects of peak-end reasoning and duration neglect is that it leads to the counterintuitive conclusion that less painful endings can cause one to prefer longer, more objectively painful (costly) sequences over less painful (less costly) sequences. The effects of experiments 3 and 4 are small but consistent with this reasoning about a preference for longer, more costly payment plans.

5 All else equal, larger payment amounts should lead to greater “pain of paying” (Zellermayer, 1996), indicated here by a lower willingness to buy. The psychophysics of mapping payment amounts to pain of paying have yet to be fully worked out, though research has shown that a number of factors are likely to be important (Prelec and Loewenstein, 1998; Gourville and Soman, 2002; Soman, 2003; Patrick and Park, 2006; Soster et al., 2014; Raghubir et al., 2017; Lee et al., 2019; Quispe-Torreblanca et al., 2019).

6 Note: Outliers were trimmed in a few cases where the guesses were obviously absurd (e.g., a $98,400 down payment, $4,400 monthly payment, 440 month car loan).

7 Aside from loans of different durations being cobbled together, there are various reasons why payments might fall over the course of a loan. A plan that involves (a) constant repayment of principal plus (b) repayment of interest on the outstanding debt would lead to falling repayments as (b) would get smaller over time (Hoelzl et al., 2011).

8 Following Varey and Kahneman (1992), participants were told that “People were paid to participate in a series of experiments. Each experiment involved some time in an uncomfortable state, such as sitting in a vibrating room, exposure to loud drilling or hissing noises, standing in an uncomfortable position, etc…The participants in each experiment made a rating every 5 minutes of the discomfort they were feeling at that moment. The last rating was made just before the end of the experiment…your task will be to provide an overall evaluation of the experience of a participant in the experiment.”

9 For this experiment as well as the others in this paper, we did not provide participants with an APR. This is probably just as well, however, because participants in a between-subjects design would have seen APRs that looked reasonably good even when they were being ripped off by the addition of extra payments. In experiments 2, 3, and 4, total payments in the “base” car condition were the Kelly Blue Book values (rather than the lower Black Book values that dealers actually use). Such cars would typically be offered with a cash rebate or “0% financing” (meaning the financing has already been built into the price). Thus, in experiments 3 or 4, for a payment plan of $500 for 44 months followed by 12 additional monthly payments of $150 per month, the APR would be under 2%. Even in Experiment 2 where $7,200 of extra payments were added over the course of 16 months, the APR would be approximately 6%—a seemingly reasonable APR, even though the extra payments jacked up the price of the car by 40%. In terms of the absolute amount paid, a post-test with 30 Mechanical Turk respondents confirmed that when presented with both the base and base + 12 month plans, participants recognized the latter was more expensive.

10 As noted, we varied stimuli to examine if effects would or would not be moderated by idiosyncratic factors such as how much money subjects imagined they were earning, when the transaction was taking place, or the types of cars. For example, we varied time of purchase because people may think about distant transactions differently than near transactions, they may think about them at different levels of abstract vs. concrete construal, and perhaps whether they call forth holistic vs. analytic processing (Liberman and Trope, 2014). By themselves, distance in the future, type of car, and imagined salary do not have huge moderating effects. However, as will be seen, there was a general pattern that transactions that seemed more imaginable from one’s current circumstances—transactions that seemed more “real”—seemed to produce bigger effects. In terms of specific parameters, the reason we used a hypothetical $60,000 salary was because this was approximately the median household income in the United States (Guzman, 2017). We added a hypothetical $10,000 in savings because, especially in the “would you buy the car now” condition, we did not want anyone to think, “No, I wouldn’t buy the car now. I don’t have any money.” Thus, we endowed them with $10,000 of savings. At $90,000 of hypothetical salary, presumably participants would assume they had some savings or disposable income they could spend.

11 mattressfirm.com/financing-options.html

12 Estimates of how many people comparison-shop their mortgages will vary (for example, according to an older Federal Reserve study, about 1/4 of borrowers contacted only one lender for their home mortgage; Lee and Hogarth, 2000). In general, comparison shopping on mortgages is probably less common than one imagines—particularly among the most vulnerable. In one study of 322 homeowners who called into a Philadelphia “predatory lending” hotline, 89% had not done any comparison shopping on their mortgage (Lewis, 2003). Terms in the subprime loan sector are likely to be particularly complicated and opaque, making attempts to shop around all the more difficult (McCoy, 2010). Readers wanting general, non-proprietary data about debt in the United States may be interested in datasets of the Federal Reserve (https://www.federalreserve.gov/data.htm) or Consumer Financial Protection Bureau (https://www.consumerfinance.gov/data-research/).
