# Supplementary material for: The Hedonics of Debt
Source: Front Psychol. 2020 Nov 17;11:537606. doi: 10.3389/fpsyg.2020.537606 (PMC7705353; doi:10.3389/fpsyg.2020.537606)
Supplement: Supplementary file 1 [file Presentation_1.pdf]

Study 1

Start of Block: Block 11

Experimenter, please input the participant number:

____

End of Block: Block 11

Start of Block: Default Question Block

UNIVERSITY OF ILLINOIS
AT URBANA-CHAMPAIGN
Department of Psychology
College of Liberal Arts and Sciences
603 East Daniel Street
Champaign, IL 61820

Informed Consent *Please read this agreement carefully. You must be 18 years or older to participate*

Purpose of the research: To better understand people's attitudes about financial matters and other important social issues.
What you will do in this survey: You will be asked to complete a questionnaire. During this survey, you will complete tasks that may include: (1) reading and responding to passages and/or (2) answering questions about yourself, your opinions, and your beliefs.

Time required: This study will take about **5 minutes** to complete.

Risks & Benefits: There are no risks (beyond what you would expect in daily life) associated with participating in this study. One benefit of this research is that you will learn more about how psychologists study attitudes.

Privacy: None of your responses are connected to your name. We will keep your responses private. However, the service hosting this survey may have access to your responses and IP number. We cannot guarantee that this service will keep your responses private. Results may be used in future classes and research. Cumulative results from this survey may be presented at conferences and/or published in books, journals, and/or in the popular media.

Participation and withdrawal: Your participation is completely voluntary. You may quit at any time without penalty. You can also skip any questions if you prefer not to answer.

Further information: If you have questions about this study, please contact Dr. Dov Cohen (email:dovcohen247@gmail.com), Department of Psychology, University of Illinois, Champaign, IL 61820. Phone: (217) 244-5830.

Who to contact about your rights in this study: If you have any questions about your rights as a participant in this study or any concerns or complaints, please contact the University of Illinois Institutional Review Board at 217-333-2670 (collect calls will be accepted if you identify yourself as a research participant) or via email at irb@illinois.edu

○ **The purpose and nature of this research have been sufficiently explained and I agree to participate in this study. I understand that I am free to withdraw at any time without incurring any penalty.** (1)

End of Block: Default Question Block

Start of Block: Block 8

In this study, we're interested in your opinions on student loans and some of the financial decisions you may make. You'll be presented with a series of scenarios, and we would like you to indicate your preference.

For your reference, students begin to make payments towards their student loans 6 months after they graduate, and the average starting salary of an undergraduate is $60,000.

End of Block: Block 8

Start of Block: 10 year - college A vs B

We're interested in your opinions on student loans. Imagine that you are graduating from high school and considering different colleges to go to. You are planning to take out a student loan to be repaid over 5 years.

What is the maximum monthly student loan payment you would be willing to pay after you graduate from college?

	200 250 300 350 400 450 500 550 600 650 700
Monthly loan payment ()	

We're interested in your opinions on student loans. Imagine that you are graduating from high school and considering different colleges to go to. You are planning to take out a student loan to be repaid over 10 years.

What is the maximum monthly student loan payment you would be willing to pay after you graduate from college?

	200 250 300 350 400 450 500 550 600 650 700
Monthly loan payment ()	

We're interested in your opinions on student loans. Imagine that you are graduating from high school and considering different colleges to go to. You are planning to take out a student loan to be repaid over 15 years.

What is the maximum monthly student loan payment you would be willing to pay after you graduate from college?

	200 250 300 350 400 450 500 550 600 650 700
Monthly loan payment ()	

End of Block: 10 year - college A vs B

Start of Block: within duration

For a 10 year student loan, what is the maximum monthly payment you would want to pay?

	200 250 300 350 400 450 500 550 600 650 700
Monthly loan payment ()	

For a 15 year student loan, what is the maximum monthly payment you would want to pay?

	200 250 300 350 400 450 500 550 600 650 700
Monthly loan payment ()	

End of Block: within duration

Start of Block: Block 8

Now assume that you've have graduated college. You have taken a new job that pays $60,000 and offers a $12,000 signing bonus. You will begin to make payments on your student loans in 6 months.

For the next few questions, you'll be asked to indicate your preference between different loans your school is offering.

Please treat each question independently.

End of Block: Block 8

Start of Block: preference for final or no final payment

Please read the scenario below and indicate your preference.

Imagine that your school offered you one of two payment plans. Please indicate which plan you prefer:

	$215 monthly payment for 3 years and a final payment of $3000	$300 monthly payment for 3 years and no final payment

	0	50	100
Preference ()			

Imagine that your school offered you one of two payment plans. Please indicate which plan you prefer:

	$300 monthly payment for 3 years and no final payment		$215 monthly payment for 3 years and a final payment of $3000
	0	50	100
Preference ()			

End of Block: preference for final or no final payment

Start of Block: preference for down or no down payment

Please read the scenario below and indicate your preference.

Imagine that your school has offered you one of two payment plans. Please indicate which plan you prefer:

	$3,000 down payment with a $215 monthly payment for 3 years	No down payment with a $300 monthly payment for 3 years
	0 50 100	
Preference ()		

Imagine that your school has offered you one of two payment plans. Please indicate which plan you prefer:

	No down payment with a $300 monthly payment for 3 years	$3,000 down payment with a $215 monthly payment for 3 years
	0 50 100	
Preference ()		

End of Block: preference for down or no down payment

Start of Block: preference for final or down payment

Please read the scenario below and indicate your preference.

Imagine that your school has offered you one of two payment plans. Please indicate which plan you prefer:

	No down payment with a<br>$215 monthly payment for<br>3 years and a final payment of $3,000	$3,000 down payment with a $215 monthly payment for 3 years and no final payment
	0 50 100	
Preference ()		

Imagine that your school has offered you one of two payment plans. Please indicate which plan you prefer:

	$3,000 down payment with a $215 monthly payment for 3 years and no final payment	No down payment with a<br>$215 monthly payment for<br>3 years and a final payment of $3,000
	0 50 100	
Preference ()		

End of Block: preference for final or down payment

Start of Block: Block 13

Think back to this question......

We're interested in your opinions on student loans. Imagine that you are graduating from high school and considering different colleges to go to. You are planning to take out a student loan.

What is the maximum monthly student loan payment you would be willing to make after you graduate from college?

What did you consider when you reported a monthly loan payment?

____

____

____

____

____

End of Block: Block 13

Start of Block: Block 9

Please answer the following demographic questions. As with all answers in this study, your answers to these questions are completely anonymous and cannot be used to identify you.

What is your sex?

- ◯ Male (1)
- ◯ Female (2)

What is your age?

____

What is your year in school?

- ◯ Freshman (1)
- ◯ Sophomore (2)
- ◯ Junior (3)
- ◯ Senior and above (4)
- ◯ Graduate Student (5)
- ◯ Not a student (6)

What is your major?

____

To attend college or other higher education, have you taken out student loans?

- ◯ Yes (1)
- ◯ No (2)
- ◯ I have no idea (3)
- ◯ I know but am unwilling to say (4)

Display This Question:

If To attend college or other higher education, have you taken out student loans? = Yes

If you have taken out loans, about how much have you taken out **per year?**

- ◯ $0 - $2,500 (1)
- ◯ $2,501 - $5,001 (2)
- ◯ $5,001 - $7,500 (3)
- ◯ $7,501 - $10,000 (4)
- ◯ $10,001 - $12,500 (5)
- ◯ $12,500-$15,000 (6)
- ◯ $15,001-$20,000 (7)
- ◯ $20,001-$25,000 (8)
- ◯ More than $25,000 (9)

End of Block: Block 9

Start of Block: Block 9

UNIVERSITY OF ILLINOIS
AT URBANA-CHAMPAIGN
Dr. Dov Cohen
Department of Psychology
College of Liberal Arts and Sciences
603 East Daniel Street
Champaign, IL 61820

DEBRIEFING
The study in which you participated today is part of a project that aims to learn about people's perceptions of debt. Specifically, we hope to understand (1) perceptions of debt among students at the University of Illinois, (2) if any differences exist in perceptions of debt among various subgroups in society, and (3) attitudes towards debtors or those who are unable to repay their debts, and (4) students' own borrowing habits.

Considering that the citizens in the United States rely heavily on credit and borrowing, investigating perceptions of both debt and of debtors is an important topic to research. To date, there has been little research on the psychology behind why people borrow or how people in the

United States view borrowing. With over 1 million filings of bankruptcy per year and 1.5 billion credit cards in the United States, debt is prevalent and common for many citizens. Additionally, there is an estimated $902 billion to $1 trillion in outstanding student loan debt in the United States today.

In addition to the prevalence of debt and credit in the United States, other developing countries are becoming more reliant on debt and credit as well. This global problem is affecting millions around the world who are unable to repay their debts. Investigating people's attitudes and perceptions towards debt is one of the first steps to mitigating this problem.

Thank you for participating!

Please do not discuss the details or hypotheses of this study with any other students as they may be potential participants, and knowing the purpose of the study beforehand could affect the results.

If you are feeling distressed as a result of this study and would like to talk to someone, please call 1-800-273-TALK to be connected to a mental health crisis line in your area.

Finally, if you would like any further information or if you have any further questions, please contact Dr. Dov Cohen at dovcohen247@gmail.com.

References for Further Reading

Porter, K. (2012). *Broke: How debt bankrupts the middle class*. Stanford: Stanford University Press.

Additionally, if you would like to increase your own financial literacy and take greater charge of your
finances, we recommend the following:

UIUC Center for Economic and Financial Education: http://www.cefe.illinois.edu/
United States Government Financial education http://www.mymoney.gov/Pages/default.aspx
Consumer Financial Protection Bureau http://www.consumerfinance.gov/
http://www.consumerfinance.gov/students/ (special section for students)

End of Block: Block 9

Study 2

Start of Block: Default Question Block

UNIVERSITY OF ILLINOIS
AT URBANA-CHAMPAIGN
Department of Psychology
College of Liberal Arts and Sciences
603 East Daniel Street
Champaign, IL 61820

Informed Consent *Please read this agreement carefully. You must be 18 years or older to participate*

Purpose of the research: To better understand people's attitudes about financial matters and other important social issues.
What you will do in this survey: You will be asked to complete a questionnaire. During this survey, you will complete tasks that may include: (1) answering or responding to scenarios and/or (2) answering questions about yourself, your opinions, and your beliefs.

Time required: This study will take about **3-5 minutes** to complete.

Risks & Benefits: There are no risks (beyond what you would expect in daily life) associated with participating in this study. One benefit of this research is that you will learn more about how psychologists study attitudes. You will also receive $0.15 for completing this study.

Privacy: None of your responses are connected to your name. We will keep your responses private. However, the service hosting this survey may have access to your responses and IP number. We cannot guarantee that this service will keep your responses private. Results may be used in future classes and research. Cumulative results from this survey may be presented at conferences and/or published in books, journals, and/or in the popular media.

Participation and withdrawal: Your participation is completely voluntary. You may quit at any time without penalty, besides not earning credit for this particular study. You can also skip any questions if you prefer not to answer.

Further information: If you have questions about this study, please contact Dr. Dov Cohen (email:dovcohen247@gmail.com), Department of Psychology, University of Illinois, Champaign, IL 61820. Phone: (217) 244-5830.

Who to contact about your rights in this study: If you have any questions about your rights as a participant in this study or any concerns or complaints, please contact the University of Illinois Institutional Review Board at 217-333-2670 (collect calls will be accepted if you identify yourself as a research participant) or via email at irb@illinois.edu

○ **The purpose and nature of this research have been sufficiently explained and I agree to participate in this study. I understand that I am free to withdraw at any time without incurring any penalty.** (1)

Timing
First Click (1)
Last Click (2)
Page Submit (3)
Click Count (4)

End of Block: Default Question Block

Start of Block: Block 2

Imagine that you just graduated college a few years ago and have taken a job across the U.S. You make $50,000 per year and are interested in purchasing a new car.

You're at a dealership and are browsing their current inventory. Please click continue to view the cars that they have available.

Please note that these cars vary in manufacturer, model, and features.

Timing
First Click (1)
Last Click (2)
Page Submit (3)
Click Count (4)

End of Block: Block 2

Start of Block: Possible Cars

Below are the cars that the dealership has available. Please look through the cars and indicate which car you like the most.

○ Year: 2014 Car Type: Four Door Sedan Engine: 1.6L
Basic warranty: 5 yr/60,000 mi
Interior: Leather Automatic Locks
Safety Ratings (5 star scale): Transmission: 6-Speed automatic Front Crash: 4/5 stars
Horsepower: 106 HP Side Crash: 5/5 stars Fuel economy: 31/40 mpg
Rollover: 4/5 stars (9)

○ Year: 2014 Car Type: Four Door Compact Engine: 1.2L
Basic warranty: 5 yr/60,000 mi
Interior: Leather seats Automatic Locks
Safety Ratings (5 star scale): Transmission: 6-Speed automatic Front Crash: 4/5 stars
Horsepower: 74 HP Side Crash: 5/5 stars Fuel economy: 37/44 mpg
Rollover: 4/5 stars (10)

○ Year: 2013 Car Type: Four Door mini MPV Mileage: 15,458 Miles
Basic warranty: 5 yr/60,000 mi
Interior: Cloth Automatic Locks
Safety Ratings (5 star scale): Transmission: 6-Speed automatic
Front Crash: 4/5 stars
Horsepower: 128 HP Side Crash: 5/5 stars Fuel economy: 27/33 mpg
Rollover: 4/5 stars Engine: 1.8L
(11)

○ Year: 2012 Car Type: Four Door mini MPV Mileage: 20,138 Miles
Basic warranty: 5 yr/60,000 mi
Interior: Cloth Automatic Locks
Safety Ratings (5 star scale): Transmission: 6-Speed automatic
Front Crash: 4/5 stars
Horsepower: 144 HP Side Crash:

5/5 stars Fuel economy: 27/31 mpg
Rollover: 4/5 stars Engine: 1.8L
(12)

○ Year: 2014 Car Type: Four Door Compact Engine: 1.2L
Basic warranty: 5 yr/60,000 mi
Interior: Leather seats Automatic Locks
Safety Ratings (5 star scale): Transmission: 6-Speed automatic Front Crash: 4/5 stars
Horsepower: 90 HP Side Crash: 5/5 stars Fuel economy: 31/39 mpg
Rollover: 4/5 stars (13)

○ Year: 2016 Car Type: Four Door Compact Engine: 1.2L
Basic warranty: 5 yr/60,000 mi
Interior: Cloth seats Automatic Locks
Safety Ratings (5 star scale): Transmission: 6-Speed automatic Front Crash: 4/5 stars
Horsepower: 74 HP Side Crash: 5/5 stars
Fuel economy: 37/44 mpg
Rollover: 4/5 stars (14)

○ Year: 2016 Car Type: Four Door Sedan Mileage: 14,583 Miles
Basic warranty: 5 yr/60,000 mi
Interior: Cloth seats Automatic Locks
Safety Ratings (5 star scale): Transmission: 6-Speed automatic Front Crash: 4/5 stars
Horsepower: 160 HP Side Crash: 5/5 stars Fuel economy: 24/35 mpg
Rollover: 4/5 stars Engine: 2.0L (15)

○ Year: 2013 Car Type: Four Door mini MPV Miles: 9,237 Miles Basic warranty: 5 yr/60,000 mi Interior: Cloth Automatic Locks Safety Ratings (5 star scale): Transmission: 6-Speed automatic Front Crash: 4/5 stars Horsepower: 125 HP Side Crash: 5/5 stars Fuel economy: 31/40 mpg Rollover: 4/5 stars Engine: 1.4L (16)

○ Year: 2016 Car Type: Four Door Compact Engine: 1.5L
Basic warranty: 5 yr/60,000 mi
Interior: Cloth Automatic Locks
Safety Ratings (5 star scale): Transmission: 6-Speed automatic
Front Crash: 4/5 stars
Horsepower: 100 HP Side Crash:

5/5 stars Fuel economy: 29/35 mpg
Rollover: 4/5 stars (17)

Timing
First Click (1)
Last Click (2)
Page Submit (3)
Click Count (4)

End of Block: Possible Cars

Start of Block: random offer

You indicated that you wanted to purchase ${car/ChoiceGroup/SelectedChoices}

Display This Question:

If final = 1

And level = 1

The dealership has offered to finance the car if you agree to pay $440 per month for the next 36 months. At the end of the 36 months, they require a final payment of $2600. Please indicate how willing you are to buy the car under the terms of this deal:

Display This Question:

If duration = 1

And level = 4

The dealership has offered to finance the car if you agree to pay $440 per month for the next 58 months. Please indicate how willing you are to buy the car under the terms of this deal:

Display This Question:

If down = 1

And level = 4

The dealership has offered to finance the car if you agree to pay an initial amount of $9800. You will then pay $440 per month for the next 36 months. Please indicate how willing you are to buy the car under the terms of this deal:

Display This Question:

If final = 1

And level = 4

The dealership has offered to finance the car if you agree to pay $440 per month for the next 36 months. At the end of the 36 months, they require a final payment of $9800. Please indicate how willing you are to buy the car under the terms of this deal:

Display This Question:

If monthly = 1

And level = 4

The dealership has offered to finance the car if you agree to pay $710 per month for the next 36 months. Please indicate how willing you are to buy the car under the terms of this deal:

Display This Question:

If duration = 1

And level = 3

The dealership has offered to finance the car if you agree to pay $440 per month for the next 53 months. Please indicate how willing you are to buy the car under the terms of this deal:

Display This Question:

If monthly = 1

And level = 3

The dealership has offered to finance the car if you agree to pay $650 per month for the next 36 months. Please indicate how willing you are to buy the car under the terms of this deal:

Display This Question:

If final = 1

And level = 3

The dealership has offered to finance the car if you agree to pay $440 per month for the next 36 months. At the end of the 36 months, they require a final payment of $7400. Please indicate how willing you are to buy the car under the terms of this deal:

Display This Question:

If down = 1

And level = 3

The dealership has offered to finance the car if you agree to pay an initial amount of $7400. You will then pay $440 per month for the next 36 months. Please indicate how willing you are to buy the car under the terms of this deal:

Display This Question:

If down = 1

And level = 2

The dealership has offered to finance the car if you agree to pay an initial amount of $5000. You will then pay $440 per month for the next 36 months. Please indicate how willing you are to buy the car under the terms of this deal:

Display This Question:

If duration = 1

And level = 2

The dealership has offered to finance the car if you agree to pay $440 per month for the next 47 months. Please indicate how willing you are to buy the car under the terms of this deal:

Display This Question:

If monthly = 1

And level = 2

The dealership has offered to finance the car if you agree to pay $580 per month for the next 36 months. Please indicate how willing you are to buy the car under the terms of this deal:

Display This Question:

If final = 1

And level = 2

The dealership has offered to finance the car if you agree to pay $440 per month for the next 36 months. At the end of the 36 months, they require a final payment of $5000. Please indicate how willing you are to buy the car under the terms of this deal:

Display This Question:

If monthly = 1

And level = 1

The dealership has offered to finance the car if you agree to pay $510 per month for the next 36 months. Please indicate how willing you are to buy the car under the terms of this deal:

Display This Question:

If duration = 1

And level = 1

The dealership has offered to finance the car if you agree to pay $440 per month for the next 42 months. Please indicate how willing you are to buy the car under the terms of this deal:

Display This Question:

If down = 1

And level = 1

The dealership has offered to finance the car if you agree to pay an initial amount of $2600. You will then pay $440 per month for the next 36 months. Please indicate how willing you are to buy the car under the terms of this deal:

	Definitely don't buy the car		Definitely buy the car
	0	50	100
Preference ()			

Timing
First Click (1)
Last Click (2)
Page Submit (3)
Click Count (4)

End of Block: random offer

Start of Block: Demographics

Please answer the following demographic questions. As with all answers in this study, your answers to these questions are completely anonymous and cannot be used to identify you.

What is your sex?

- ○ Male (1)
- ○ Female (2)

What is your age?

____

Have you ever purchased a car?

- ○ Yes (1)
- ○ No (2)

How did you pay for the car?

- ○ Payment Plan (1)
- ○ Paid cash in full (2)
- ○ I have never purchased a car. (3)

If you had a payment plan, what were the terms for your loan? (ex. Monthly payment, Duration, Interest Rate)

____

____

____

____

____

With which racial or ethnic group do you identify? (Check all that apply)

- ▢ American Indian or Alaska Native (1)
- ▢ Black or African American (2)
- ▢ Native Hawaiian or other Pacific Islander (3)
- ▢ Asian (4)
- ▢ Hispanic, Latino, or Spanish (5)
- ▢ White (6)
- ▢ Don't Know (7)
- ▢ Other (8) ____

What is your current religion?

- ○ Christian - Catholic (1)
- ○ Christian - Protestant (2)
- ○ Christian - Orthodox (ex. Russian Orthodox, Greek Orthodox, etc.) (3)
- ○ Christian, but I do not belong to a specific denomination (4)
- ○ Christian, non-practicing (5)
- ○ Mormon (10)
- ○ Jewish (6)
- ○ Muslim (7)
- ○ Hindu (11)
- ○ Just spiritual, believe in God (8)
- ○ Atheist/Agnostic (12)
- ○ I don't practice any religion (13)
- ○ Other (9) ____

When you were growing up, what was your family's religion?

- ○ Christian - Catholic (1)
- ○ Christian - Protestant (2)
- ○ Christian - Orthodox (ex. Russian Orthodox, Greek Orthodox, etc.) (3)
- ○ Christian, but I do not belong to a specific denomination (4)
- ○ Christian, non-practicing (5)
- ○ Mormon (10)
- ○ Jewish (6)
- ○ Muslim (7)
- ○ Hindu (11)
- ○ Just spiritual, believe in God (8)
- ○ Atheist/Agnostic (12)
- ○ We didn't practice any religion (13)
- ○ Other (9) ____

What was your total household income before taxes during the past 12 months?

____

Do you manage the finances in your household?

- ○ Yes (1)
- ○ No (2)

What is the highest degree of education you have received?

- ○ Less than high school (1)
- ○ High school or GED (2)
- ○ Associate/Junior College (3)
- ○ Bachelors degree (4)
- ○ Masters, PhD, J.D., M.D. or similar degree (5)

Timing
First Click (1)
Last Click (2)
Page Submit (3)
Click Count (4)

End of Block: Demographics

Start of Block: Block 21

Display This Question:

If accuracy = 1

We are interested in your recall of the loan offers that were made to you during this study. As a reward for accuracy, if you get within 10 pct of the correct answer for all loan terms, we will triple the payment to you.

Please try to recall the terms of the FIRST loan offer that they gave you. Please try to remember

it as accurately as possible, and only input a numeric answer. If the offer did not contain one of these features, please leave it blank or put 0.

- Down payment (1) ____
- Length of loan (in months) (2) ____
- Monthly payment (3) ____
- Final payment (4) ____

Display This Question:

If [Preference] != 100

Please try to recall the terms of the LAST loan offer that they gave you. Please try to remember it as accurately as possible, and only input a numeric answer. If the offer did not contain one of these features, please leave it blank or put 0.

- Down payment (1) ____
- Length of loan (in months) (2) ____
- Monthly payment (3) ____
- Final payment (4) ____

Timing
First Click (1)
Last Click (2)
Page Submit (3)
Click Count (4)

End of Block: Block 21

Start of Block: Block 24

Display This Question:

If accuracy = 1

If you would like to qualify for this bonus, please insert your mturk ID.

____

End of Block: Block 24

Start of Block: Purpose

In as much detail as possible, please describe what **you think the purpose** of the study was. If you are not sure, please give **your best guess**.

____

____

____

____

____

Do you have any additional comments or thoughts about the study?

____

____

____

____

____

End of Block: Purpose

Start of Block: Debriefing

UNIVERSITY OF ILLINOIS
AT URBANA-CHAMPAIGN
Dr. Dov Cohen

Department of Psychology
College of Liberal Arts and Sciences
603 East Daniel Street
Champaign, IL 61820

DEBRIEFING

The study in which you participated today is part of a project that aims to learn about people's preferences for payment scenarios. We predict that when individuals evaluate payment plans, they will be sensitive to the end of the sequence and ignore the duration of the payment plan. We also predict that they will be sensitive to the monthly payment.

Considering that the citizens in the United States rely heavily on credit and borrowing, investigating why individuals make the financial choices that they do is an important topic to research. To date, there has been little research on the psychology behind why people borrow or how people in the United States view borrowing. With over 1 million filings of bankruptcy per year and 1.5 billion credit cards in the United States, debt is prevalent and common for many citizens.

There is also an estimated $1 trillion in outstanding auto loan. More than 60% of auto loans are at least 60 months long, and often, choosing such a long car loan term is disadvantageous for the borrower. Typically, the longer the finance, the more interest you will have to pay on it, both in terms of the rate itself and the finance charges over time.

By conducting this research, we hope to learn which factors contribute to people's financial decisions.

Thank you for participating!

Please do not discuss the details or hypotheses of this study with any other students as they may be potential participants, and knowing the purpose of the study beforehand could affect the results.

If you are feeling distressed as a result of this study and would like to talk to someone, please call 1-800-273-TALK to be connected to a mental health crisis line in your area.

Finally, if you would like any further information or if you have any further questions, please contact Dr. Dov Cohen at dovcohen247@gmail.com.

References for Further Reading

Porter, K. (2012). *Broke: How debt bankrupts the middle class*. Stanford: Stanford University Press.

Additionally, if you would like to increase your own financial literacy and take greater charge of your
finances, we recommend the following:

United States Government Financial education http://www.mymoney.gov/Pages/default.aspx
Consumer Financial Protection Bureau http://www.consumerfinance.gov/
http://www.consumerfinance.gov/students/ (special section for students)

End of Block: Debriefing

Start of Block: Exclude Down - level 1

Your answer of ${answer1/ChoiceNumericEntryValue/1} is lower than the dealership likes to see to close a deal. The dealership would like to make a second and FINAL offer. (There will be no third offer).

The second and FINAL offer is below:

${car/ChoiceGroup/SelectedChoices}

The dealership has offered to finance the car if you agree to pay $510 per month for the next 36 months. Please indicate how willing you are to buy the car under the terms of this deal:

The dealership has offered to finance the car if you agree to pay $440 per month for the next 42 months. Please indicate how willing you are to buy the car under the terms of this deal:

	Definitely don't buy the car		Definitely buy the car
	0	50	100
Preference ()			

Timing
First Click (1)
Last Click (2)
Page Submit (3)
Click Count (4)

End of Block: Exclude Down - level 1

Start of Block: Exclude Final - Level 1

Your answer of ${answer1/ChoiceNumericEntryValue/1} is lower than the dealership likes to see to close a deal. The dealership would like to make a second and FINAL offer. (There will be no third offer).

The second and FINAL offer is below:

${car/ChoiceGroup/SelectedChoices}

The dealership has offered to finance the car if you agree to pay $510 per month for the next 36 months. Please indicate how willing you are to buy the car under the terms of this deal:

The dealership has offered to finance the car if you agree to pay $440 per month for the next 42 months. Please indicate how willing you are to buy the car under the terms of

this deal:

	Definitely don't buy the car 0	50	Definitely buy the car 100
Preference ()			

Timing
First Click (1)
Last Click (2)
Page Submit (3)
Click Count (4)

End of Block: Exclude Final - Level 1

Start of Block: Exclude Duration - Level 1

Your answer of ${answer1/ChoiceNumericEntryValue/1} is lower than the dealership likes to see to close a deal. The dealership would like to make a second and FINAL offer. (There will be no third offer).

The second and FINAL offer is below:

${car/ChoiceGroup/SelectedChoices}

The dealership has offered to finance the car if you agree to pay $510 per month for the next 36 months. Please indicate how willing you are to buy the car under the terms of this deal:

The dealership has offered to finance the car if you agree to pay an initial amount of $2600. You will then pay $440 per month for the next 36 months. Please indicate how willing you are to buy the car under the terms of this deal:

The dealership has offered to finance the car if you agree to pay $440 per month for the next 36 months. At the end of the 36 months, they require a final payment of $2600. Please indicate how willing you are to buy the car under the terms of this deal:

	Definitely don't buy the car		Definitely buy the car
	0	50	100
Preference ()			

Timing
First Click (1)
Last Click (2)
Page Submit (3)
Click Count (4)

End of Block: Exclude Duration - Level 1

Start of Block: Exclude Monthly - Level 1

Your answer of ${answer1/ChoiceNumericEntryValue/1} is lower than the dealership likes to see to close a deal. The dealership would like to make a second and FINAL offer. (There will be no third offer).

The second and FINAL offer is below:

${car/ChoiceGroup/SelectedChoices}

The dealership has offered to finance the car if you agree to pay $440 per month for the next 42 months. Please indicate how willing you are to buy the car under the terms of this deal:

The dealership has offered to finance the car if you agree to pay an initial amount of $2600. You will then pay $440 per month for the next 36 months. Please indicate how willing you are to buy the car under the terms of this deal:

The dealership has offered to finance the car if you agree to pay $440 per month for the next 36 months. At the end of the 36 months, they require a final payment of $2600. Please indicate how willing you are to buy the car under the terms of this deal:

	Definitely don't buy the car 0	50	Definitely buy the car 100
Preference ()			

Timing
First Click (1)
Last Click (2)
Page Submit (3)
Click Count (4)

End of Block: Exclude Monthly - Level 1

Start of Block: Exclude Down - Level 2

Your answer of ${answer1/ChoiceNumericEntryValue/1} is lower than the dealership likes to see to close a deal. The dealership would like to make a second and FINAL offer. (There will be no third offer).

The second and FINAL offer is below:

${car/ChoiceGroup/SelectedChoices}

The dealership has offered to finance the car if you agree to pay $440 per month for the next 47 months. Please indicate how willing you are to buy the car under the terms of this deal:

The dealership has offered to finance the car if you agree to pay $580 per month for the next 36 months. Please indicate how willing you are to buy the car under the terms of this deal:

	Definitely don't buy the car		Definitely buy the car
	0	50	100
Preference ()			

Timing
First Click (1)
Last Click (2)
Page Submit (3)
Click Count (4)

End of Block: Exclude Down - Level 2

Start of Block: Exclude Final - Level 2

Your answer of ${answer1/ChoiceNumericEntryValue/1} is lower than the dealership likes to see to close a deal. The dealership would like to make a second and FINAL offer. (There will be no third offer).

The second and FINAL offer is below:

${car/ChoiceGroup/SelectedChoices}

The dealership has offered to finance the car if you agree to pay $440 per month for the next 47 months. Please indicate how willing you are to buy the car under the terms of this deal:

The dealership has offered to finance the car if you agree to pay $580 per month for the next 36 months. Please indicate how willing you are to buy the car under the terms of this deal:

	Definitely don't buy the car		Definitely buy the car
	0	50	100
Preference ()	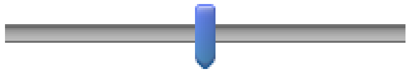		

Timing
First Click (1)
Last Click (2)
Page Submit (3)
Click Count (4)

End of Block: Exclude Final - Level 2

Start of Block: Exclude Duration - Level 2

Your answer of ${answer1/ChoiceNumericEntryValue/1} is lower than the dealership likes to see to close a deal. The dealership would like to make a second and FINAL offer. (There will be no third offer).

The second and FINAL offer is below:

${car/ChoiceGroup/SelectedChoices}

The dealership has offered to finance the car if you agree to pay $440 per month for the next 36 months. At the end of the 36 months, they require a final payment of $5000. Please indicate how willing you are to buy the car under the terms of this deal:

The dealership has offered to finance the car if you agree to pay an initial amount of $5000. You will then pay $440 per month for the next 36 months. Please indicate how willing you are to buy the car under the terms of this deal:

The dealership has offered to finance the car if you agree to pay $580 per month for the next 36 months. Please indicate how willing you are to buy the car under the terms of this deal:

	Definitely don't buy the car 0	50	Definitely buy the car 100
Preference ()			

Timing
First Click (1)
Last Click (2)
Page Submit (3)
Click Count (4)

End of Block: Exclude Duration - Level 2

Start of Block: Exclude monthly - level 2

Your answer of ${answer1/ChoiceNumericEntryValue/1} is lower than the dealership likes to see to close a deal. The dealership would like to make a second and FINAL offer. (There will be no third offer).

The second and FINAL offer is below:
${car/ChoiceGroup/SelectedChoices}

The dealership has offered to finance the car if you agree to pay $440 per month for the next 47 months. Please indicate how willing you are to buy the car under the terms of this deal:

The dealership has offered to finance the car if you agree to pay $440 per month for the next 36 months. At the end of the 36 months, they require a final payment of $5000. Please indicate how willing you are to buy the car under the terms of this deal:

The dealership has offered to finance the car if you agree to pay an initial amount of $5000. You will then pay $440 per month for the next 36 months. Please indicate how willing you are to buy the car under the terms of this deal:

	Definitely don't buy the car		Definitely buy the car
	0	50	100
Preference ()			

Timing
First Click (1)
Last Click (2)
Page Submit (3)
Click Count (4)

End of Block: Exclude monthly - level 2

Start of Block: Exclude final - level 3

Your answer of ${answer1/ChoiceNumericEntryValue/1} is lower than the dealership likes to see to close a deal. The dealership would like to make a second and FINAL offer. (There will be no third offer).

The second and FINAL offer is below:

${car/ChoiceGroup/SelectedChoices}

The dealership has offered to finance the car if you agree to pay $440 per month for the next 53 months. Please indicate how willing you are to buy the car under the terms of this deal:

The dealership has offered to finance the car if you agree to pay $650 per month for the next 36 months. Please indicate how willing you are to buy the car under the terms of this deal:

	Definitely don't buy the car		Definitely buy the car
	0	50	100
Preference ()			

Timing
First Click (1)
Last Click (2)
Page Submit (3)
Click Count (4)

End of Block: Exclude final - level 3

Start of Block: Exclude Down - Level 3

Your answer of ${answer1/ChoiceNumericEntryValue/1} is lower than the dealership likes to see to close a deal. The dealership would like to make a second and FINAL offer. (There will be no third offer).

The second and FINAL offer is below:

${car/ChoiceGroup/SelectedChoices}

The dealership has offered to finance the car if you agree to pay $440 per month for the next 53 months. Please indicate how willing you are to buy the car under the terms of this deal:

The dealership has offered to finance the car if you agree to pay $650 per month for the next 36 months. Please indicate how willing you are to buy the car under the terms of this deal:

	Definitely don't buy the car		Definitely buy the car
	0	50	100
Preference ()			

Timing
First Click (1)
Last Click (2)
Page Submit (3)
Click Count (4)

End of Block: Exclude Down - Level 3

Start of Block: Exclude Duration - Level 3

Your answer of ${answer1/ChoiceNumericEntryValue/1} is lower than the dealership likes to see to close a deal. The dealership would like to make a second and FINAL offer. (There will be no third offer).

The second and FINAL offer is below:

${car/ChoiceGroup/SelectedChoices}

The dealership has offered to finance the car if you agree to pay $650 per month for the next 36 months. Please indicate how willing you are to buy the car under the terms of this deal:

The dealership has offered to finance the car if you agree to pay an initial amount of $7400. You will then pay $440 per month for the next 36 months. Please indicate how willing you are to buy the car under the terms of this deal:

The dealership has offered to finance the car if you agree to pay $440 per month for the next 36 months. At the end of the 36 months, they require a final payment of $7400. Please indicate how willing you are to buy the car under the terms of this deal:

	Definitely don't buy the car		Definitely buy the car
	0	50	100
Preference ()			

Timing
First Click (1)
Last Click (2)
Page Submit (3)
Click Count (4)

End of Block: Exclude Duration - Level 3

Start of Block: Exclude Monthly - Level 3

Your answer of ${answer1/ChoiceNumericEntryValue/1} is lower than the dealership likes to see to close a deal. The dealership would like to make a second and FINAL offer. (There will be no third offer).

The second and FINAL offer is below:

${car/ChoiceGroup/SelectedChoices}

The dealership has offered to finance the car if you agree to pay $440 per month for the next 53 months. Please indicate how willing you are to buy the car under the terms of this deal:

The dealership has offered to finance the car if you agree to pay an initial amount of $7400. You will then pay $440 per month for the next 36 months. Please indicate how willing you are to buy the car under the terms of this deal:

The dealership has offered to finance the car if you agree to pay $440 per month for the next 36 months. At the end of the 36 months, they require a final payment of $7400. Please indicate how willing you are to buy the car under the terms of this deal:

	Definitely don't buy the car		Definitely buy the car
	0	50	100
Preference ()			

Timing
First Click (1)
Last Click (2)
Page Submit (3)
Click Count (4)

End of Block: Exclude Monthly - Level 3

Start of Block: Exclude down - level 4

Your answer of ${answer1/ChoiceNumericEntryValue/1} is lower than the dealership likes to see to close a deal. The dealership would like to make a second and FINAL offer. (There will be no third offer).

The second and FINAL offer is below:
${car/ChoiceGroup/SelectedChoices}

The dealership has offered to finance the car if you agree to pay $440 per month for the next 58 months. Please indicate how willing you are to buy the car under the terms of this deal:

The dealership has offered to finance the car if you agree to pay $710 per month for the next 36 months. Please indicate how willing you are to buy the car under the terms of this deal:

	Definitely don't buy the car		Definitely buy the car
	0	50	100
Preference ()			

Timing
First Click (1)
Last Click (2)
Page Submit (3)
Click Count (4)

End of Block: Exclude down - level 4

Start of Block: Exclude final - level 4

Your answer of ${answer1/ChoiceNumericEntryValue/1} is lower than the dealership likes to see to close a deal. The dealership would like to make a second and FINAL offer. (There will be no third offer).

 The second and FINAL offer is below:

${car/ChoiceGroup/SelectedChoices}

The dealership has offered to finance the car if you agree to pay $440 per month for the next 58 months. Please indicate how willing you are to buy the car under the terms of this deal:

The dealership has offered to finance the car if you agree to pay $710 per month for the next 36 months. Please indicate how willing you are to buy the car under the terms of this deal:

	Definitely don't buy the car		Definitely buy the car
	0	50	100
Preference ()			

Timing
First Click (1)
Last Click (2)
Page Submit (3)
Click Count (4)

End of Block: Exclude final - level 4

Start of Block: Exclude duration - level 4

Your answer of ${answer1/ChoiceNumericEntryValue/1} is lower than the dealership likes to see to close a deal. The dealership would like to make a second and FINAL offer. (There will be no third offer).

The second and FINAL offer is below:

${car/ChoiceGroup/SelectedChoices}

The dealership has offered to finance the car if you agree to pay $440 per month for the next 36 months. At the end of the 36 months, they require a final payment of $9800. Please indicate how willing you are to buy the car under the terms of this deal:

The dealership has offered to finance the car if you agree to pay an initial amount of $9800. You will then pay $440 per month for the next 36 months. Please indicate how willing you are to buy the car under the terms of this deal:

The dealership has offered to finance the car if you agree to pay $710 per month for the next 36 months. Please indicate how willing you are to buy the car under the terms of this deal:

	Definitely don't buy the car		Definitely buy the car
	0	50	100
Preference ()			

Timing
First Click (1)
Last Click (2)
Page Submit (3)
Click Count (4)

End of Block: Exclude duration - level 4

Start of Block: Exclude monthly - level 4

Your answer of ${answer1/ChoiceNumericEntryValue/1} is lower than the dealership likes to see to close a deal. The dealership would like to make a second and FINAL offer. (There will be no third offer).

 The second and FINAL offer is below:

${car/ChoiceGroup/SelectedChoices}

The dealership has offered to finance the car if you agree to pay $440 per month for the next 58 months. Please indicate how willing you are to buy the car under the terms of this deal:

The dealership has offered to finance the car if you agree to pay $440 per month for the next 36 months. At the end of the 36 months, they require a final payment of $9800. Please indicate how willing you are to buy the car under the terms of this deal:

The dealership has offered to finance the car if you agree to pay an initial amount of $9800. You will then pay $440 per month for the next 36 months. Please indicate how willing you are to buy the car under the terms of this deal:

Definitely don't buy the car		Definitely buy the car
0	50	100

Preference ()	

Timing
First Click (1)
Last Click (2)
Page Submit (3)
Click Count (4)

End of Block: Exclude monthly - level 4

Study 3

Start of Block: Block 70

UNIVERSITY OF ILLINOIS
AT URBANA-CHAMPAIGN
Department of Psychology
College of Liberal Arts and Sciences
603 East Daniel Street
Champaign, IL 61820
Informed Consent
Please read this agreement carefully. You must be 18 years or older to participate

Purpose of the research: To better understand people's attitudes about financial matters and other important social issues.
What you will do in this survey: You will be asked to complete a questionnaire. During this survey, you will complete tasks that may include: (1) reading and responding to scenarios and/or (2) answering questions about yourself, your opinions, and your beliefs.

Time required: This study will take about **10 minutes** to complete.

Risks & Benefits: There are no risks (beyond what you would expect in daily life) associated with participating in this study. One benefit of this research is that you will learn more about how psychologists study attitudes. You will also receive $.50 for completing this study.

Privacy: None of your responses are connected to your name. We will keep your responses private. We will not make any attempt to link your Prolific ID to any identifiable information that may exist on the Internet. However, the service hosting this survey may have access to your responses and IP number. We cannot guarantee that this service will keep your responses private. Results may be used in future classes and research. Cumulative results from this survey may be presented at conferences and/or published in books, journals, and/or in the popular media.

Participation and withdrawal: Your participation is completely voluntary. You may quit at any time without penalty, besides the loss of the monetary compensation. You can also skip any questions if you prefer not to answer.

Further information: If you have questions about this study, please contact Dr. Dov Cohen (email:dovcohen247@gmail.com), Department of Psychology, University of Illinois, Champaign, IL 61820. Phone: (217) 244-5830.

Who to contact about your rights in this study: If you have any questions about your rights as a participant in this study or any concerns or complaints, please contact the University of Illinois Institutional Review Board at 217-333-2670 or via email at irb@illinois.edu

○ **The purpose and nature of this research have been sufficiently explained and I agree to participate in this study. I understand that I am free to withdraw at any time without incurring any penalty.** (1)

End of Block: Block 70

Start of Block: Default Question Block

Thank you for participating in our study! Today you will be reading over some scenarios and answering questions. Please take your time and read over everything carefully.

End of Block: Default Question Block

Start of Block: 20k condition 1 Instructions

For the next questions, imagine that you are currently making $60,000 per year. You currently have $10,000 in the bank and have been searching for a car to buy.

For the following questions, you will see a car, along with some of its specifications. You will have to review all of the information and indicate your preference.

Page Break

For all these cars, the dealership is willing to give you a loan. The financing will be from, say, a national bank, a local bank, or the manufacturer.

Please treat each question independently. Your answer as to whether you would buy or not buy one car should not affect your answers for other cars.

Please note that these cars vary in manufacturer, model, mileage, age, and features.

End of Block: 20k condition 1 Instructions

Start of Block: Hyundai Sonata 22k- 36 months base

Year: 2016 Car Type: Four Door Sedan Engine: 2.4 L 4-Cylinder
Basic warranty: 3 yr/36,000 mi
Interior: Cloth Fuel Economy: 25 city/38 highway
Horsepower: 185 hp Transmission: 6-speed automatic

The dealership has agreed to finance the car with a 36 month plan. During the 36 months, you will pay $610 per month. Please indicate how willing you are to purchase the car:

	Definitely don't buy the car		Definitely buy the car
	0	50	100
Preference ()			

End of Block: Hyundai Sonata 22k- 36 months base

Start of Block: 2016 scion tc - 22k 40 months

Year: 2016 Car Type: Four Door SportWagen Engine: 1.8 L 4-Cylinder
Basic warranty: 3 yr/36,000 mi

Interior: Cloth Fuel Economy: 25 city/35 highway Horsepower: 170 hp Transmission: 6-speed automatic

The dealership has agreed to finance the car with a 40 month plan. During the 40 months, you will pay $550 per month. Please indicate how willing you are to purchase the car:

	Definitely don't buy the car		Definitely buy the car
	0	50	100
Preference ()			

End of Block: 2016 scion tc - 22k 40 months

Start of Block: NIssan Juke 44 months base - 20k

Year: 2016 Car Type: Four Door Compact SUV Engine: 1.6 L 4-Cylinder Basic warranty: 3 yr/36,000 mi Interior: Cloth Fuel Economy: 28 city/32 highway Horsepower: 188 hp Transmission: 6-speed automatic

The dealership has agreed to finance the car with a 44 month plan. During the 44 months, you will pay $500 per month. Please indicate how willing you are to purchase the car:

	Definitely don't buy the car		Definitely buy the car
	0	50	100
Preference ()			

End of Block: NIssan Juke 44 months base - 20k

Start of Block: 48 months 20k - dodge journey

Year: 2016 Car Type: Four Door Compact SUV Engine: 2.4 L 4-Cylinder
Basic warranty: 3 yr/36,000 mi
Interior: Cloth Fuel Economy: 19 city/26 highway
Horsepower: 173 hp Transmission: 6-speed automatic

The dealership has agreed to finance the car with a 48 month plan. During the 48 months, you will pay $458 per month. Please indicate how willing you are to purchase the car:

	**Definitely don't buy the car**		**Definitely buy the car**
	0	50	100
Preference ()			

End of Block: 48 months 20k - dodge journey

Start of Block: 20k madza cx5 52 months

Year: 2016 Car Type: Four Door SUV Engine: 2.5 L 4-Cylinder
Basic warranty: 3 yr/36,000 mi
Interior: Cloth Fuel Economy: 26 city/33 highway
Horsepower: 185 hp Transmission: 6-speed Automatic

The dealership has agreed to finance the car with a 52 month plan. During the 52 months, you will pay $423 per month. Please indicate how willing you are to purchase the car:

	**Definitely don't buy the car**		**Definitely buy the car**
	0	50	100
Preference ()			

End of Block: 20k madza cx5 52 months

Start of Block: Ford Fusion 22k - 56base

Year: 2016 Car Type: Four Door Sedan Engine: 2.5 L 4-Cylinder
Basic warranty: 3 yr/36,000 mi
Interior: Cloth Fuel Economy: 22 city/34 highway
Horsepower: 175 hp Transmission: 6-speed automatic

The dealership has agreed to finance the car with a 56 month plan. During the 56 months, you will pay $393 per month. Please indicate how willing you are to purchase the car:

	**Definitely don't buy the car**		**Definitely buy the car**
	0	50	100
Preference ()			

End of Block: Ford Fusion 22k - 56base

Start of Block: 20kcondition2

For the next questions, imagine that you are currently making $60,000 per year. You currently have $10,000 in the bank and have been searching for a car to buy.

For the following questions, you will see a car, along with some of its specifications. You will have to review all of the information and indicate your preference.

Page Break

For all these cars, the dealership is willing to give you a loan package. This means the financing will come from a variety of sources (say, partly, from a national bank, partly from a local bank, or partly from the manufacturer).

Conveniently, these loans will all be bundled into 1 package, so that you can make a single payment towards your car. However, because some loans are longer and some are shorter, this means the monthly payment may change over the life of the loan.

Please treat each question independently. Your answer as to whether you would buy or not buy one car should not affect your answers for other cars.

Please note that these cars vary in manufacturer, model, mileage, age, and features.

End of Block: 20kcondition2

Start of Block: Hyundai Sonata - 36 base plus $150 per month

Display This Question:

If Phrase = 1

Year: 2016 Car Type: Four Door Sedan Engine: 2.4 L 4-Cylinder
Basic warranty: 3 yr/36,000 mi
Interior: Cloth Fuel Economy: 25 city/38 highway
Horsepower: 185 hp Transmission: 6-speed automatic

The dealership has agreed to finance the car with a 48 month plan. During the first 36 months, you will pay $610 per month. For the last 12 months, you will pay $150 per month. Please indicate how willing you are to purchase the car:

	Definitely don't buy the car		Definitely buy the car
	0	50	100
Preference ()			

Display This Question:

If Phrase = 2

Year: 2016 Car Type: Four Door Sedan Engine: 2.4 L 4-Cylinder
Basic warranty: 3 yr/36,000 mi
Interior: Cloth Fuel Economy: 25 city/38 highway
Horsepower: 185 hp Transmission: 6-speed automatic

The dealership has agreed to finance the car with a 48 month plan. During the first 36 months, you will pay $610 per month. For the last 12 months, your payments are reduced to $150 per month. Please indicate how willing you are to purchase the car:

	Definitely don't buy the car		Definitely buy the car
	0	50	100
Preference ()			

Display This Question:

If Phrase = 3

Year: 2016 Car Type: Four Door Sedan Engine: 2.4 L 4-Cylinder
Basic warranty: 3 yr/36,000 mi
Interior: Cloth Fuel Economy: 25 city/38 highway
Horsepower: 185 hp Transmission: 6-speed automatic

The dealership has agreed to finance the car with a 48 month plan. During the first 36 months, you will pay $610 per month. For the last 12 months, your payments decrease to $150 per month. Please indicate how willing you are to purchase the car:

	Definitely don't buy the car		Definitely buy the car
	0	50	100
Preference ()			

End of Block: Hyundai Sonata - 36 base plus $150 per month

Start of Block: Volkswagen 40 base plus 150 for 12 months

Display This Question:

If Phrase = 1

Year: 2016 Car Type: Four Door SportWagen Engine: 1.8 L 4-Cylinder Basic warranty: 3 yr/36,000 mi Interior: Cloth Fuel Economy: 25 city/35 highway Horsepower: 170 hp Transmission: 6-speed automatic

The dealership has agreed to finance the car with a 52 month plan. During the first 40 months, you will pay $550 per month. For the last 12 months, you will pay $150 per month. Please indicate how willing you are to purchase the car:

	Definitely don't buy the car		Definitely buy the car
	0	50	100
Preference ()			

Display This Question:

If Phrase = 2

Year: 2016 Car Type: Four Door SportWagen Engine: 1.8 L 4-Cylinder Basic warranty: 3 yr/36,000 mi Interior: Cloth Fuel Economy: 25 city/35 highway Horsepower: 170 hp Transmission: 6-speed automatic

The dealership has agreed to finance the car with a 52 month plan. During the first 40 months, you will pay $550 per month. For the last 12 months, your payments are reduced to $150 per month. Please indicate how willing you are to purchase the car:

Definitely don't buy the car		Definitely buy the car
0	50	100

Preference ()	

Display This Question:

If Phrase = 3

Year: 2016 Car Type: Four Door SportWagen Engine: 1.8 L 4-Cylinder
Basic warranty: 3 yr/36,000 mi
Interior: Cloth Fuel Economy: 25 city/35 highway
Horsepower: 170 hp Transmission: 6-speed automatic

The dealership has agreed to finance the car with a 52 month plan. During the first 40 months, you will pay $550 per month. For the last 12 months, your payments decrease to $150 per month. Please indicate how willing you are to purchase the car:

	Definitely don't buy the car		Definitely buy the car
	0	50	100
Preference ()			

End of Block: Volkswagen 40 base plus 150 for 12 months

Start of Block: Nissan Juke - 44 months plus $150 for 12 months

Display This Question:

If Phrase = 1

Year: 2016 Car Type: Four Door Compact SUV Engine: 1.6 L 4-Cylinder
Basic warranty: 3 yr/36,000 mi
Interior: Cloth Fuel Economy: 28 city/32 highway
Horsepower: 188 hp Transmission: 6-speed automatic

The dealership has agreed to finance the car with a 56 month plan. During the first 44

months, you will pay $500 per month. For the last 12 months, you will pay $150 per month. Please indicate how willing you are to purchase the car:

	**Definitely don't buy the car**		**Definitely buy the car**
	0	50	100
Preference ()			

Display This Question:

If Phrase = 2

Year: 2016 Car Type: Four Door Compact SUV Engine: 1.6 L 4-Cylinder
Basic warranty: 3 yr/36,000 mi
Interior: Cloth Fuel Economy: 28 city/32 highway
Horsepower: 188 hp Transmission: 6-speed automatic

The dealership has agreed to finance the car with a 56 month plan. During the first 44 months, you will pay $500 per month. For the last 12 months, your payments are reduced to $150 per month. Please indicate how willing you are to purchase the car:

	**Definitely don't buy the car**		**Definitely buy the car**
	0	50	100
Preference ()			

Display This Question:

If Phrase = 3

Year: 2016 Car Type: Four Door Compact SUV Engine: 1.6 L 4-Cylinder
Basic warranty: 3 yr/36,000 mi
Interior: Cloth Fuel Economy: 28 city/32 highway
Horsepower: 188 hp Transmission: 6-speed automatic

The dealership has agreed to finance the car with a 56 month plan. During the first 44 months, you will pay $500 per month. For the last 12 months, your payments decrease to $150 per month. Please indicate how willing you are to purchase the car:

	Definitely don't buy the car		Definitely buy the car
	0	50	100
Preference ()			

End of Block: Nissan Juke - 44 months plus $150 for 12 months

Start of Block: dodge journey - 20k 48 months plus $150 per month

Display This Question:

If Phrase = 1

Year: 2016 Car Type: Four Door Compact SUV Engine: 2.4 L 4-Cylinder Basic warranty: 3 yr/36,000 mi Interior: Cloth Fuel Economy: 19 city/26 highway Horsepower: 173 hp Transmission: 6-speed automatic

The dealership has agreed to finance the car with a 60 month plan. During the first 48 months, you will pay $458 per month. For the last 12 months, you will pay $150 per month. Please indicate how willing you are to purchase the car:

	Definitely don't buy the car		Definitely buy the car
	0	50	100
Preference ()			

Display This Question:

If Phrase = 2

Year: 2016 Car Type: Four Door Compact SUV Engine: 2.4 L 4-Cylinder
Basic warranty: 3 yr/36,000 mi
Interior: Cloth Fuel Economy: 19 city/26 highway
Horsepower: 173 hp Transmission: 6-speed automatic

The dealership has agreed to finance the car with a 60 month plan. During the first 48 months, you will pay $458 per month. For the last 12 months, your payments will be reduced to $150 per month. Please indicate how willing you are to purchase the car:

	Definitely don't buy the car		Definitely buy the car
	0	50	100
Preference ()			

Display This Question:

If Phrase = 3

Year: 2016 Car Type: Four Door Compact SUV Engine: 2.4 L 4-Cylinder
Basic warranty: 3 yr/36,000 mi
Interior: Cloth Fuel Economy: 19 city/26 highway
Horsepower: 173 hp Transmission: 6-speed automatic

The dealership has agreed to finance the car with a 60 month plan. During the first 48 months, you will pay $458 per month. For the last 12 months, your payments will decrease to $150 per month. Please indicate how willing you are to purchase the car:

	Definitely don't buy the car		Definitely buy the car
	0	50	100
Preference ()			

End of Block: dodge journey - 20k 48 months plus $150 per month

Start of Block: subaru impreza - 52 months plus 150/12 months

Display This Question:

If Phrase = 1

Year: 2016 Car Type: Four Door SUV Engine: 2.5 L 4-Cylinder
Basic warranty: 3 yr/36,000 mi
Interior: Cloth Fuel Economy: 26 city/33 highway
Horsepower: 185 hp Transmission: 6-speed Automatic

The dealership has agreed to finance the car with a 64 month plan. During the first 52 months, you will pay $423 per month. For the last 12 months, you will pay $150 per month. Please indicate how willing you are to purchase the car:

	Definitely don't buy the car		Definitely buy the car
	0	50	100
Preference ()			

Display This Question:

If Phrase = 2

Year: 2016 Car Type: Four Door SUV Engine: 2.5 L 4-Cylinder
Basic warranty: 3 yr/36,000 mi
Interior: Cloth Fuel Economy: 26 city/33 highway
Horsepower: 185 hp Transmission: 6-speed Automatic

The dealership has agreed to finance the car with a 64 month plan. During the first 52 months, you will pay $423 per month. For the last 12 months, your payments are reduced to $150 per month. Please indicate how willing you are to purchase the car:

	Definitely don't buy the car		Definitely buy the car
	0	50	100

Preference ()	

Display This Question:

If Phrase = 3

Year: 2016 Car Type: Four Door SUV Engine: 2.5 L 4-Cylinder Basic warranty: 3 yr/36,000 mi Interior: Cloth Fuel Economy: 26 city/33 highway Horsepower: 185 hp Transmission: 6-speed Automatic

The dealership has agreed to finance the car with a 64 month plan. During the first 52 months, you will pay $423 per month. For the last 12 months, your payments decrease to $150 per month. Please indicate how willing you are to purchase the car:

	**Definitely don't buy the car**		**Definitely buy the car**
	0	50	100
Preference ()			

End of Block: subaru impreza - 52 months plus 150/12 months

Start of Block: Ford Fusion 22k - 56 months base + $150/month

Display This Question:

If Phrase = 1

Year: 2016 Car Type: Four Door Sedan Engine: 2.5 L 4-Cylinder Basic warranty: 3 yr/36,000 mi Interior: Cloth Fuel Economy: 22 city/34 highway Horsepower: 175 hp Transmission: 6-speed automatic

The dealership has agreed to finance the car with a 68 month plan. During the first 56

months, you will pay $393 per month. For the last 12 months, you will pay $150 per month. Please indicate how willing you are to purchase the car:

	**Definitely don't buy the car**		**Definitely buy the car**
	0	50	100
Preference ()			

Display This Question:

If Phrase = 2

Year: 2016 Car Type: Four Door Sedan Engine: 2.5 L 4-Cylinder Basic warranty: 3 yr/36,000 mi Interior: Cloth Fuel Economy: 22 city/34 highway Horsepower: 175 hp Transmission: 6-speed automatic

The dealership has agreed to finance the car with a 68 month plan. During the first 56 months, you will pay $393 per month. For the last 12 months, your payments will be reduced to $150 per month. Please indicate how willing you are to purchase the car:

	**Definitely don't buy the car**		**Definitely buy the car**
	0	50	100
Preference ()			

Display This Question:

If Phrase = 3

Year: 2016 Car Type: Four Door Sedan Engine: 2.5 L 4-Cylinder Basic warranty: 3 yr/36,000 mi Interior: Cloth Fuel Economy: 22 city/34 highway Horsepower: 175 hp Transmission: 6-speed automatic

The dealership has agreed to finance the car with a 68 month plan. During the first 56 months, you will pay $393 per month. For the last 12 months, your payments decrease to $150 per month. Please indicate how willing you are to purchase the car:

	Definitely don't buy the car		Definitely buy the car
	0	50	100
Preference ()			

End of Block: Ford Fusion 22k - 56 months base + $150/month

Start of Block: pain instructions 1

We are now interested in people's intuitions about uncomfortable experiences. People were paid to participate in a series of experiments.

Each experiment involved some time in an uncomfortable state, such as sitting in a vibrating room, exposure to loud drilling or hissing noises, standing in an uncomfortable position, etc.

The participants were told at the beginning of each experience how long it would last. Although the experimental conditions did not change in the course of a session, the subjective experience of discomfort changed over time.

The participants in each experiment made a rating every 5 minutes of the discomfort they were feeling at that moment. The last rating was made just before the end of the experiment.

These rating are on a scale from 0 to 10, where 0 = no discomfort at all and 10 = almost unbearable.

End of Block: pain instructions 1

Start of Block: pain instructions 2

In each of the following questions, you will be given the average discomfort rating made in an experiment, and your task will be to provide an overall evaluation of the experience of a participant in that experiment.

In interpreting the discomfort ratings, you should keep in mind that the participants

served in a series of such experiments, and were highly trained in the use of the discomfort scale.

In particular, they were instructed to use the scale consistently, so that a rating of 5, for example, indicates the same level of subjective discomfort at the beginning, in the middle, or at the end of an experimental session.

You should also assume that the experiences have no after-effects of pain or discomfort - the participants return very quickly to a normal level of comfort.

For each set of these pain ratings, we would like you to provide a global evaluation of how bad the overall experience is using a scale from 0 to 100 (0 = not bad at all, 100 = extremely bad).

End of Block: pain instructions 2

Start of Block: 7 7 7 7 between

The ratings are on a scale from 0 to 10, where 0 = no discomfort at all and 10 = almost unbearable. Please provide a global evaluation of how bad the overall experience is using a scale from 0 to 100 (0 = not bad at all, 100 = extremely bad).

7 7 7 7 7 7 7

	**Not bad at all**		**Extremely bad**
	0	50	100
Global Evaluation ()			

End of Block: 7 7 7 7 between

Start of Block: 3 8 7 8 7 8 5 7 between

The ratings are on a scale from 0 to 10, where 0 = no discomfort at all and 10 = almost unbearable. Please provide a global evaluation of how bad the overall experience is using a scale from 0 to 100 (0 = not bad at all, 100 = extremely bad).

3 8 7 8 7 8 5 7

	Not bad at all		Extremely bad
	0	50	100
Global Evaluation ()			

End of Block: 3 8 7 8 7 8 5 7 between

Start of Block: 2 5 4 4 7 9 6 6 between

The ratings are on a scale from 0 to 10, where 0 = no discomfort at all and 10 = almost unbearable. Please provide a global evaluation of how bad the overall experience is using a scale from 0 to 100 (0 = not bad at all, 100 = extremely bad).

2 5 4 4 7 9 6 6

	Not bad at all		Extremely bad
	0	50	100
Global Evaluation ()			

End of Block: 2 5 4 4 7 9 6 6 between

Start of Block: 6 9 3 7 9 8 6 between

The ratings are on a scale from 0 to 10, where 0 = no discomfort at all and 10 = almost unbearable. Please provide a global evaluation of how bad the overall experience is using a scale from 0 to 100 (0 = not bad at all, 100 = extremely bad).

6 9 3 7 9 8 6

	Not bad at all		Extremely bad
	0	50	100
Global Evaluation ()			

End of Block: 6 9 3 7 9 8 6 between

Start of Block: 9 3 8 8 7 4 6 4 between

The ratings are on a scale from 0 to 10, where 0 = no discomfort at all and 10 = almost unbearable. Please provide a global evaluation of how bad the overall experience is using a scale from 0 to 100 (0 = not bad at all, 100 = extremely bad).

9 3 8 8 7 4 6 4

	Not bad at all		Extremely bad
	0	50	100
Global Evaluation ()			

End of Block: 9 3 8 8 7 4 6 4 between

Start of Block: 6 9 3 7 9 8 6 1 between

The ratings are on a scale from 0 to 10, where 0 = no discomfort at all and 10 = almost unbearable. Please provide a global evaluation of how bad the overall experience is using a scale from 0 to 100 (0 = not bad at all, 100 = extremely bad).

6 9 3 7 9 8 6 1

	Not bad at all	Extremely bad
	0 50 100	
Global Evaluation ()		

End of Block: 6 9 3 7 9 8 6 1 between

Start of Block: 9 3 8 8 7 4 6 4 1 between

The ratings are on a scale from 0 to 10, where 0 = no discomfort at all and 10 = almost unbearable. Please provide a global evaluation of how bad the overall experience is using a scale from 0 to 100 (0 = not bad at all, 100 = extremely bad).

9 3 8 8 7 4 6 4 1

	Not bad at all	Extremely bad
	0 50 100	
Global Evaluation ()		

End of Block: 9 3 8 8 7 4 6 4 1 between

Start of Block: 2 5 4 4 7 9 6 6 2 between

The ratings are on a scale from 0 to 10, where 0 = no discomfort at all and 10 = almost unbearable. Please provide a global evaluation of how bad the overall experience is using a scale from 0 to 100 (0 = not bad at all, 100 = extremely bad).

2 5 4 4 7 9 6 6 2

	Not bad at all		Extremely bad
	0	50	100
Global Evaluation ()			

End of Block: 2 5 4 4 7 9 6 6 2 between

Start of Block: 7 7 7 7 2 between

The ratings are on a scale from 0 to 10, where 0 = no discomfort at all and 10 = almost unbearable. Please provide a global evaluation of how bad the overall experience is using a scale from 0 to 100 (0 = not bad at all, 100 = extremely bad).

7 7 7 7 7 7 7 2

	Not bad at all		Extremely bad
	0	50	100
Global Evaluation ()			

End of Block: 7 7 7 7 2 between

Start of Block: 3 8 7 8 7 8 5 7 1

The ratings are on a scale from 0 to 10, where 0 = no discomfort at all and 10 = almost unbearable. Please provide a global evaluation of how bad the overall experience is using a scale from 0 to 100 (0 = not bad at all, 100 = extremely bad).

3 8 7 8 7 8 5 7 1

	Not bad at all		Extremely bad
	0	50	100
Global Evaluation ()			

End of Block: 3 8 7 8 7 8 5 7 1

Start of Block: 6 6 6 6 2

The ratings are on a scale from 0 to 10, where 0 = no discomfort at all and 10 = almost unbearable. Please provide a global evaluation of how bad the overall experience is using a scale from 0 to 100 (0 = not bad at all, 100 = extremely bad).

6 2 5 7 6 3 4 5 2

	Not bad at all		Extremely bad
	0	50	100
Global Evaluation ()			

End of Block: 6 6 6 6 2

Start of Block: 6 6 6 6 9

The ratings are on a scale from 0 to 10, where 0 = no discomfort at all and 10 = almost unbearable. Please provide a global evaluation of how bad the overall experience is using a scale from 0 to 100 (0 = not bad at all, 100 = extremely bad).

6 2 5 7 6 3 4 5 9

	Not bad at all		Extremely bad
	0	50	100
Global Evaluation ()			

End of Block: 6 6 6 6 9

Start of Block: 6 6 6 6

The ratings are on a scale from 0 to 10, where 0 = no discomfort at all and 10 = almost unbearable. Please provide a global evaluation of how bad the overall experience is using a scale from 0 to 100 (0 = not bad at all, 100 = extremely bad).

6 2 5 7 6 3 4 5

Not bad at all	Extremely bad

	0	50	100
Global Evaluation ()			

End of Block: 6 6 6 6

Start of Block: 2 6 1 3 5 5 8 6

The ratings are on a scale from 0 to 10, where 0 = no discomfort at all and 10 = almost unbearable. Please provide a global evaluation of how bad the overall experience is using a scale from 0 to 100 (0 = not bad at all, 100 = extremely bad).

2 6 1 3 5 4 8 6

	**Not bad at all**		**Extremely bad**
	0	50	100
Global Evaluation ()			

End of Block: 2 6 1 3 5 5 8 6

Start of Block: 2 6 1 3 5 4 8 6 9

The ratings are on a scale from 0 to 10, where 0 = no discomfort at all and 10 = almost unbearable. Please provide a global evaluation of how bad the overall experience is using a scale from 0 to 100 (0 = not bad at all, 100 = extremely bad).

2 6 1 3 5 4 8 6 9

	Not bad at all		Extremely bad
	0	50	100
Global Evaluation ()			

End of Block: 2 6 1 3 5 4 8 6 9

Start of Block: 2 6 1 3 5 4 6 8 2

The ratings are on a scale from 0 to 10, where 0 = no discomfort at all and 10 = almost unbearable. Please provide a global evaluation of how bad the overall experience is using a scale from 0 to 100 (0 = not bad at all, 100 = extremely bad).

2 6 1 3 5 4 6 8 2

	Not bad at all		Extremely bad
	0	50	100
Global Evaluation ()			

End of Block: 2 6 1 3 5 4 6 8 2

Start of Block: 7 4 2 9 7 5 7 4

The ratings are on a scale from 0 to 10, where 0 = no discomfort at all and 10 = almost unbearable. Please provide a global evaluation of how bad the overall experience is using a scale from 0 to 100 (0 = not bad at all, 100 = extremely bad).

7 4 2 9 7 5 7 4

	Not bad at all		Extremely bad
	0	50	100
Global Evaluation ()			

End of Block: 7 4 2 9 7 5 7 4

Start of Block: 7 4 2 9 7 5 7 4 9

The ratings are on a scale from 0 to 10, where 0 = no discomfort at all and 10 = almost unbearable. Please provide a global evaluation of how bad the overall experience is using a scale from 0 to 100 (0 = not bad at all, 100 = extremely bad).

7 4 2 9 7 5 7 4 9

	Not bad at all		Extremely bad
	0	50	100
Global Evaluation ()			

End of Block: 7 4 2 9 7 5 7 4 9

Start of Block: 7 4 2 9 7 5 7 4 1

The ratings are on a scale from 0 to 10, where 0 = no discomfort at all and 10 = almost unbearable. Please provide a global evaluation of how bad the overall experience is using a scale from 0 to 100 (0 = not bad at all, 100 = extremely bad).

7 4 2 9 7 5 7 4 1

	Not bad at all		Extremely bad
	0	50	100
Global Evaluation ()			

End of Block: 7 4 2 9 7 5 7 4 1

Start of Block: 1 4 8 3 8 8 7 5 9

The ratings are on a scale from 0 to 10, where 0 = no discomfort at all and 10 = almost unbearable. Please provide a global evaluation of how bad the overall experience is using a scale from 0 to 100 (0 = not bad at all, 100 = extremely bad).

1 4 8 3 8 8 7 5 9

	Not bad at all		Extremely bad
	0	50	100
Global Evaluation ()			

End of Block: 1 4 8 3 8 8 7 5 9

Start of Block: 1 4 8 3 8 8 7 5

The ratings are on a scale from 0 to 10, where 0 = no discomfort at all and 10 = almost unbearable. Please provide a global evaluation of how bad the overall experience is using a scale from 0 to 100 (0 = not bad at all, 100 = extremely bad).

1 4 8 3 8 8 7 5

	Not bad at all		Extremely bad
	0	50	100
Global Evaluation ()			

End of Block: 1 4 8 3 8 8 7 5

Start of Block: 1 4 8 3 8 8 7 5 2

The ratings are on a scale from 0 to 10, where 0 = no discomfort at all and 10 = almost unbearable. Please provide a global evaluation of how bad the overall experience is using a scale from 0 to 100 (0 = not bad at all, 100 = extremely bad).

1 4 8 3 8 8 7 5 2

	Not bad at all		Extremely bad
	0	50	100
Global Evaluation ()			

End of Block: 1 4 8 3 8 8 7 5 2

Start of Block: 4 7 8 9 8 7 6 9

The ratings are on a scale from 0 to 10, where 0 = no discomfort at all and 10 = almost unbearable. Please provide a global evaluation of how bad the overall experience is using a scale from 0 to 100 (0 = not bad at all, 100 = extremely bad).

4 7 8 9 8 7 6 9

	Not bad at all		Extremely bad
	0	50	100
Global Evaluation ()			

End of Block: 4 7 8 9 8 7 6 9

Start of Block: 4 7 8 9 8 7 6

The ratings are on a scale from 0 to 10, where 0 = no discomfort at all and 10 = almost unbearable. Please provide a global evaluation of how bad the overall experience is using a scale from 0 to 100 (0 = not bad at all, 100 = extremely bad).

4 7 8 9 8 7 6

	Not bad at all		Extremely bad
	0	50	100
Global Evaluation ()			

End of Block: 4 7 8 9 8 7 6

Start of Block: 4 7 8 9 8 7 6 1

The ratings are on a scale from 0 to 10, where 0 = no discomfort at all and 10 = almost unbearable. Please provide a global evaluation of how bad the overall experience is using a scale from 0 to 100 (0 = not bad at all, 100 = extremely bad).

4 7 8 9 8 7 6 1

	Not bad at all		Extremely bad
	0	50	100
Global Evaluation ()			

End of Block: 4 7 8 9 8 7 6 1

Start of Block: Demographics

Before you proceed to the next task, we'd like you to answer a few demographic questions. As with all answers in this study, your answers to these questions are completely anonymous and cannot be used to identify you.

Page Break

What is your sex?

- ○ Male (1)
- ○ Female (2)

What is your age?

____

With which racial or ethnic group do you identify? (Check all that apply)

- ☐ American Indian or Alaska Native (1)
- ☐ Black or African American (2)
- ☐ Native Hawaiian or other Pacific Islander (3)
- ☐ Asian (4)
- ☐ Hispanic, Latino, or Spanish (5)
- ☐ White (6)
- ☐ Don't Know (7)
- ☐ Other (8) ____

What is your current religion?

- ○ Christian - Catholic (1)
- ○ Christian - Protestant (2)
- ○ Christian - Orthodox (ex. Russian Orthodox, Greek Orthodox, etc.) (3)
- ○ Christian, but I do not belong to a specific denomination (4)
- ○ Christian, non-practicing (5)
- ○ Mormon (10)
- ○ Jewish (6)
- ○ Muslim (7)
- ○ Hindu (11)
- ○ Just spiritual, believe in God (8)
- ○ Atheist/Agnostic (12)
- ○ I don't practice any religion (13)
- ○ Other (9) ____

When you were growing up, what was your family's religion?

- Christian - Catholic (1)
- Christian - Protestant (2)
- Christian - Orthodox (ex. Russian Orthodox, Greek Orthodox, etc.) (3)
- Christian, but I do not belong to a specific denomination (4)
- Christian, non-practicing (5)
- Mormon (10)
- Jewish (6)
- Muslim (7)
- Hindu (11)
- Just spiritual, believe in God (8)
- Atheist/Agnostic (12)
- We didn't practice any religion (13)
- Other (9) ____

End of Block: Demographics

Start of Block: Car 25k condition1Instructions

Now imagine that it's 15 years later, and you're making $90,000 per year. You have been searching for a new car.

For the following questions, you will see a car, along with some of its specifications.

Page Break

For all these cars, the dealership is willing to give you a loan package. This means the financing will come from a variety of sources (say, partly, from a national bank, partly from a local bank, or partly from the manufacturer).
Conveniently, these loans will all be bundled into 1 package, so that you can make a single payment towards your car. However, because some loans are longer and some are shorter, this means the monthly payment may change over the life of the loan.

Please treat each question independently. Your answer as to whether you would buy or not buy one car should not affect your answers for other cars. Also, your responses about whether you would buy or not buy a car 15 years ago should not affect your answers to the cars you are considering now.

Please note that these cars vary in manufacturer, model, age, and features.

End of Block: Car 25k condition1Instructions

Start of Block: Toyota Camry - 36 months + $190/12months

Display This Question:

If Phrase = 1

Year: 2016 *Car Type: Four Door Sedan* *Engine: 2.5 L 4-Cylinder*
Basic warranty: 3 yr/36,000 mi
Interior: Cloth *Fuel Economy: 26 city/37 highway*
Horsepower: 182 hp *Transmission: 6-speed automatic*

The dealership has agreed to finance the car with a 48 month plan. During the first 36 months, you will pay $695 per month. For the last 12 months, you will pay $190 per month. Please indicate how willing you are to buy the car:

	Definitely don't buy the car		Definitely buy the car
	0	50	100

Preference ()	

Display This Question:

If Phrase = 2

Year: 2016 Car Type: Four Door Sedan Engine: 2.5 L 4-Cylinder
Basic warranty: 3 yr/36,000 mi
Interior: Cloth Fuel Economy: 26 city/37 highway
Horsepower: 182 hp Transmission: 6-speed automatic

The dealership has agreed to finance the car with a 48 month plan. During the first 36 months, you will pay $695 per month. For the last 12 months, your payments are reduced to $190 per month. Please indicate how willing you are to buy the car:

	Definitely don't buy the car		Definitely buy the car
	0	50	100
Preference ()			

Display This Question:

If Phrase = 3

Year: 2016 Car Type: Four Door Sedan Engine: 2.5 L 4-Cylinder
Basic warranty: 3 yr/36,000 mi
Interior: Cloth Fuel Economy: 26 city/37 highway
Horsepower: 182 hp Transmission: 6-speed automatic

The dealership has agreed to finance the car with a 48 month plan. During the first 36

months, you will pay $695 per month. For the last 12 months, your payments decrease to $190 per month. Please indicate how willing you are to buy the car:

	Definitely don't buy the car		Definitely buy the car
	0	50	100
Preference ()			

End of Block: Toyota Camry - 36 months + $190/12months

Start of Block: 25k - 2015 Ford Escape - 25k 40 months plus 12months/$190

Display This Question:

If Phrase = 1

The dealership has agreed to finance the car with a 52 month plan. During the first 40 months, you will pay $625 per month. For the last 12 months, you will pay $190 per month. How willing are you to purchase the car?

	Definitely don't buy the car		Definitely buy the car
	0	50	100
Preference ()			

Display This Question:

If Phrase = 2

The dealership has agreed to finance the car with a 52 month plan. During the first 40

months, you will pay $625 per month. For the last 12 months, your payments are reduced to $190 per month. How willing are you to purchase the car?

	Definitely don't buy the car		Definitely buy the car
	0	50	100
Preference ()			

Display This Question:

If Phrase = 3

The dealership has agreed to finance the car with a 52 month plan. During the first 40 months, you will pay $625 per month. For the last 12 months, your payments decrease to $190 per month. How willing are you to purchase the car?

	Definitely don't buy the car		Definitely buy the car
	0	50	100
Preference ()			

End of Block: 25k - 2015 Ford Escape - 25k 40 months plus 12months/$190

Start of Block: 2016 Toyota Rav4 25k - 44month + $190/12 months

Display This Question:

If Phrase = 1

The dealership has agreed to finance the car with a 56 month plan. During the first 44

months, you will pay $570 per month. For the last 12 months, you will pay $190 per month. How willing are you to purchase the car?

	Definitely don't buy the car		Definitely buy the car
	0	50	100
Preference ()			

Display This Question:

If Phrase = 2

The dealership has agreed to finance the car with a 56 month plan. During the first 44 months, you will pay $570 per month. For the last 12 months, your payments are reduced to $190 per month. How willing are you to purchase the car?

	Definitely don't buy the car		Definitely buy the car
	0	50	100
Preference ()			

Display This Question:

If Phrase = 3

The dealership has agreed to finance the car with a 56 month plan. During the first 44 months, you will pay $570 per month. For the last 12 months, your payments decrease to $190 per month. How willing are you to purchase the car?

	Definitely don't buy the car		Definitely buy the car
	0	50	100
Preference ()			

End of Block: 2016 Toyota Rav4 25k - 44month + $190/12 months

Start of Block: 2016 Kia Sportage - 48 months + $190/12 months

Display This Question:

If Phrase = 1

Year: 2016 Car Type: Four Door Compact SUV Engine: 2.4 L 4-Cylinder Basic warranty: 5 yr/60,000 mi Interior: Cloth Fuel Economy: 21 city/28 highway Horsepower: 182 hp Transmission: 6-speed automatic **The dealership has agreed to finance the car with a 60 month plan. During the first 48 months, you will pay $520 per month. For the last 12 months, you will pay $190 per month. Please indicate how willing you are to buy the car:**

	**Definitely don't buy the car**		**Definitely buy the car**
	0	50	100
Preference ()			

Display This Question:

If Phrase = 2

Year: 2016 Car Type: Four Door Compact SUV Engine: 2.4 L 4-Cylinder Basic warranty: 5 yr/60,000 mi Interior: Cloth Fuel Economy: 21 city/28 highway Horsepower: 182 hp Transmission: 6-speed automatic

The dealership has agreed to finance the car with a 60 month plan. During the first 48 months, you will pay $520 per month. For the last 12 months, your payments are reduced to $190 per month. Please indicate how willing you are to buy the car:

	**Definitely don't buy the car**		**Definitely buy the car**
	0	50	100

Preference ()	

Display This Question:

If Phrase = 3

Year: 2016 Car Type: Four Door Compact SUV Engine: 2.4 L 4-Cylinder Basic warranty: 5 yr/60,000 mi Interior: Cloth Fuel Economy: 21 city/28 highway Horsepower: 182 hp Transmission: 6-speed automatic **The dealership has agreed to finance the car with a 60 month plan. During the first 48 months, you will pay $520 per month. For the last 12 months, your payments decrease to $190 per month. Please indicate how willing you are to buy the car:**

	**Definitely don't buy the car**		**Definitely buy the car**
	0	50	100
Preference ()			

End of Block: 2016 Kia Sportage - 48 months + $190/12 months

Start of Block: 25k- 2016 Mazda cx touring - 52 months plus 190/12 months

Display This Question:

If Phrase = 1

Year: 2016 Car Type: Four Door Compact SUV Engine: 2.5 L 4-Cylinder Basic warranty: 3 yr/36,000 mi Interior: Cloth Fuel Economy: 26 city/33 highway Horsepower: 184 hp Transmission: 6-speed automatic

The dealership has agreed to finance the car with a 64 month plan. During the first 52

months, you will pay $480 per month. For the last 12 months, you will pay $190 per month. Please indicate how willing you are to purchase the car:

	Definitely don't buy the car		Definitely buy the car
	0	50	100
Preference ()			

Display This Question:

If Phrase = 2

Year: 2016 Car Type: Four Door Compact SUV Engine: 2.5 L 4-Cylinder Basic warranty: 3 yr/36,000 mi Interior: Cloth Fuel Economy: 26 city/33 highway Horsepower: 184 hp Transmission: 6-speed automatic

The dealership has agreed to finance the car with a 64 month plan. During the first 52 months, you will pay $480 per month. For the last 12 months, your payments will be reduced to $190 per month. Please indicate how willing you are to purchase the car:

	Definitely don't buy the car		Definitely buy the car
	0	50	100
Preference ()			

Display This Question:

If Phrase = 3

Year: 2016 Car Type: Four Door Compact SUV Engine: 2.5 L 4-Cylinder Basic warranty: 3 yr/36,000 mi Interior: Cloth Fuel Economy: 26 city/33 highway Horsepower: 184 hp Transmission: 6-speed automatic

The dealership has agreed to finance the car with a 64 month plan. During the first 52 months, you will pay $480 per month. For the last 12 months, your payments decrease to $190 per month. Please indicate how willing you are to purchase the car:

	Definitely don't buy the car		Definitely buy the car
	0	50	100
Preference ()			

End of Block: 25k- 2016 Mazda cx touring - 52 months plus 190/12 months

Start of Block: 56 months 25k

Display This Question:

If Phrase = 1

The dealership has agreed to finance the car with a 68 month plan. During the first 56 months, you will pay $446 per month. For the last 12 months, you will pay $190 per month. Please indicate how willing you are to purchase the car:

	Definitely don't buy the car		Definitely buy the car
	0	50	100
Preference ()			

Display This Question:

If Phrase = 2

The dealership has agreed to finance the car with a 68 month plan. During the first 56 months, you will pay $446 per month. For the last 12 months, your payments will be reduced to $190 per month. Please indicate how willing you are to purchase the car:

	Definitely don't buy the car		Definitely buy the car
	0	50	100

Preference ()	

Display This Question:

If Phrase = 3

The dealership has agreed to finance the car with a 68 month plan. During the first 56 months, you will pay $446 per month. For the last 12 months, your payments decrease to $190 per month. Please indicate how willing you are to purchase the car:

	Definitely don't buy the car		Definitely buy the car
	0	50	100
Preference ()			

End of Block: 56 months 25k

Start of Block: 25kcondition2

Now imagine that it's 15 years later, and you're making $90,000 per year.

You have been searching for a new car. For the following questions, you will see a car, along with some of its specifications.

Page Break

For all these cars, the dealership is willing to give you a loan. The financing will be from, say, a national bank, a local bank, or the manufacturer. Because there is a single source of financing, the monthly payment will not decrease or increase over the life of the loan.

Please treat each question independently. Your answer as to whether you would buy or not buy one car should not affect your answers for other cars. Also, your responses about whether you would buy or not buy a car 15 years ago should not affect your answers to the cars you are considering now.

Please note that these cars vary in manufacturer, model, age, and features.

End of Block: 25kcondition2

Start of Block: 25k - Nissan Altima - 36 months base

Year: 2016 Car Type: Four Door Sedan Engine: 2.5 L 4-Cylinder
Basic warranty: 3 yr/36,000 mi
Interior: Cloth Fuel Economy: 26 city/37 highway
Horsepower: 182 hp Transmission: 6-speed automatic

The dealership has agreed to finance the car with a 36 month plan. During the 36 months, you will pay $695 per month. Please indicate how willing you are to purchase the car:

	**Definitely don't buy the car**	**Definitely buy the car**
	0 50	100
Preference ()		

End of Block: 25k - Nissan Altima - 36 months base

Start of Block: 25k - Honda Accord 2 Door Coupe - 40 months

The dealership has agreed to finance the car with a 40 month plan. During the 40 months, you will pay $625 per month. Please indicate how willing you are to purchase the car:

	Definitely don't buy the car 0	50	Definitely buy the car 100
Preference ()			

End of Block: 25k - Honda Accord 2 Door Coupe - 40 months

Start of Block: 2016 Ford Mustang 25k - 44 month base

The dealership has agreed to finance the car with a 44 month plan. During the 44 months, you will pay $570 per month. Please indicate how willing you are to purchase the car:

	Definitely don't buy the car 0	50	Definitely buy the car 100
Preference ()			

End of Block: 2016 Ford Mustang 25k - 44 month base

Start of Block: 2015 Volkswagen Golf GTI - 48 month

Year: 2016 Car Type: Four Door Compact SUV Engine: 2.4 L 4-Cylinder Basic warranty: 5 yr/60,000 mi Interior: Cloth Fuel Economy: 21 city/28 highway Horsepower: 182 hp Transmission: 6-speed automatic

The dealership has agreed to finance the car with a 48 month plan. During the 48 months, you will pay $520 per month. Please indicate how willing you are to purchase the car:

	Definitely don't buy the car		Definitely buy the car
	0	50	100
Preference ()			

End of Block: 2015 Volkswagen Golf GTI - 48 month

Start of Block: 25k - 2016 CHevrolet Equinox 52 months base

Year: 2016 Car Type: Four Door Compact SUV Engine: 2.5 L 4-Cylinder Basic warranty: 3 yr/36,000 mi Interior: Cloth Fuel Economy: 26 city/33 highway Horsepower: 184 hp Transmission: 6-speed automatic

The dealership has agreed to finance the car with a 52 month plan. During the 52 months, you will pay $480 per month. Please indicate how willing you are to purchase the car:

	Definitely don't buy the car		Definitely buy the car
	0	50	100

Preference ()	

End of Block: 25k - 2016 CHevrolet Equinox 52 months base

Start of Block: 56 month plan base - 25k

The dealership has agreed to finance the car with a 56 month plan. During the 56 months, you will pay $446 per month. Please indicate how willing you are to purchase the car:

	Definitely don't buy the car		Definitely buy the car
	0	50	100
Preference ()			

End of Block: 56 month plan base - 25k

Start of Block: Financial Literacy

Please try to respond as honestly and accurately as possible. Please read each statement carefully.

I find it painful to spend money.

- 1 Strongly disagree (1)
- 2 (2)
- 3 (3)
- 4 (4)
- 5 (5)
- 6 (6)
- 7 Strongly agree (7)

People who know me say that I am very thrifty.

- 1 Strongly disagree (1)
- 2 (2)
- 3 (3)
- 4 (4)
- 5 (5)
- 6 (6)
- 7 Strongly agree (7)

Money is for spending.

- 1 Strongly disagree (1)
- 2 (2)
- 3 (3)
- 4 (4)
- 5 (5)
- 6 (6)
- 7 Strongly agree (7)

The thought of spending a lot of money makes me anxious.

- 1 Strongly disagree (1)
- 2 (2)
- 3 (3)
- 4 (4)
- 5 (5)
- 6 (6)
- 7 Strongly agree (7)

I'd rather save money than spend it.

- 1 Strongly disagree (1)
- 2 (2)
- 3 (3)
- 4 (4)
- 5 (5)
- 6 (6)
- 7 Strongly agree (7)

Spending money is easy for me.

- 1 Strongly disagree (1)
- 2 (2)
- 3 (3)
- 4 (4)
- 5 (5)
- 6 (6)
- 7 Strongly agree (7)

Page Break

Which of the following description fits you better?

- Tightwad (difficulty spending money) 1 (1)
- 2 (8)
- 3 (2)
- 4 (3)
- 5 (4)
- About the same or neither 6 (5)
- 7 (6)
- 8 (7)
- 9 (9)
- 10 (10)
- Spendthrift (difficulty controlling spending)11 (11)

Some people have trouble limiting their spending: they often spend money - for example on clothes, meals, vacations, phone calls- when they would do better not to.

Other people have trouble spending money. Perhaps because spending money makes them anxious, they often don't spend money on things they should spend it on.

How well does the first description fit you? That is, do you have trouble limiting your spending?

- Never 1 (1)
- Rarely 2 (8)
- Sometimes 3 (2)
- Often 4 (3)
- Always 5 (4)

How well does the second description fit you? That is, do you have trouble spending money?

- Never 1 (1)
- Rarely 2 (8)
- Sometimes 3 (2)
- Often 4 (3)
- Always 5 (4)

Page Break

Following is a scenario describing the behavior of two shoppers. After reading about each shopper, pleaser answer the question that follows.

Mr. A is accompanying a good friend who is on a shopping spree at a local mall. When they enter a large department store, Mr. A sees that the store has a "one-day-only-sale" where everything is priced 10-60% off. He realizes he doesn't need anything, yet can't resist and ends up spending almost $100 on stuff.
Mr. B is accompanying a good friend who is on a shopping spree at a local mall. When they enter a large department store, Mr. B sees that the store has a "one-day-only-sale" where everything is priced 10-60% off. He figures he can get great deals on many items that he needs, yet the thought of spending the money keeps him from buying the stuff.

In terms of your own behavior, who are you more similar to, Mr. A or Mr. B?

- ○ Mr. A 1 (12)
- ○ 2 (13)
- ○ About the same or neither 3 (14)
- ○ 4 (15)
- ○ Mr. B 5 (16)

End of Block: Financial Literacy

Start of Block: Other Demographics

Please answer the following demographic questions. As with all answers in this study, your answers to these questions are completely anonymous and cannot be used to identify you.

Have you ever purchased a car?

- ○ Yes (1)
- ○ No (2)

How did you pay for the car?

- ◯ Payment Plan (1)
- ◯ Paid cash in full (2)
- ◯ I have never purchased a car. (3)

If you had a payment plan, what were the terms for your loan? (ex. Monthly payment, Duration, Interest Rate)

____

____

____

____

____

What was your total household income before taxes during the past 12 months?

____

Do you manage the finances in your household?

- ◯ Yes (1)
- ◯ No (2)

What is the highest degree of education you have received?

- ○ Less than high school (1)
- ○ High school or GED (2)
- ○ Associate/Junior College (3)
- ○ Bachelors degree (4)
- ○ Masters, PhD, J.D., M.D. or similar degree (5)

What is your mturk worker ID?

____

End of Block: Other Demographics

Start of Block: Block 69

In as much detail as possible, please describe what **you think the purpose** of the study was. If you are not sure, please give **your best guess**.

____

____

____

____

____

Do you have any additional comments or thoughts about the study?

____

____

____

____

End of Block: Block 69

Start of Block: Block 41

UNIVERSITY OF ILLINOIS
AT URBANA-CHAMPAIGN
Dr. Dov Cohen
Department of Psychology
College of Liberal Arts and Sciences
603 East Daniel Street
Champaign, IL 61820

DEBRIEFING

The study in which you participated today is part of a project that aims to learn about people's preferences for payment scenarios and how they relate to people's preferences about pain and unpleasantness.

Considering that the citizens in the United States rely heavily on credit and borrowing, investigating why individuals make the financial choices that they do is an important topic to research. To date, there has been little research on the psychology behind why people borrow or how people in the United States view borrowing. With over 1 million filings of bankruptcy per year and 1.5 billion credit cards in the United States, debt is prevalent and common for many citizens.

By conducting this research, we hope to learn which factors contribute to people's financial decisions.

Thank you for participating!

Please do not discuss the details or hypotheses of this study with any other people as they may be potential participants, and knowing the purpose of the study beforehand could affect the results.

If you are feeling distressed as a result of this study and would like to talk to someone, please call 1-800-273-TALK to be connected to a mental health crisis line in your area.

Finally, if you would like any further information or if you have any further questions, please contact Dr. Dov Cohen at dovcohen247@gmail.com.

References for Further Reading

Porter, K. (2012). *Broke: How debt bankrupts the middle class*. Stanford: Stanford University Press.

Additionally, if you would like to increase your own financial literacy and take greater charge of your
finances, we recommend the following:

United States Government Financial educationhttp://www.mymoney.gov/Pages/default.aspx
Consumer Financial Protection Bureau http://www.consumerfinance.gov/
http://www.consumerfinance.gov/students/ (special section for students)

End of Block: Block 41

Study 4

Start of Block: Default Question Block

UNIVERSITY OF ILLINOIS
AT URBANA-CHAMPAIGN
Department of Psychology
College of Liberal Arts and Sciences
603 East Daniel Street
Champaign, IL 61820
Informed Consent
Please read this agreement carefully. You must be 18 years or older to participate

Purpose of the research: To better understand people's attitudes about financial matters and other important social issues.
What you will do in this survey: You will be asked to complete a questionnaire. During this survey, you will complete tasks that may include: (1) reading and responding to scenarios and/or (2) answering questions about yourself, your opinions, and your beliefs.

Time required: This study will take about **1-3 minutes** to complete.

Risks & Benefits: There are no risks (beyond what you would expect in daily life) associated with participating in this study. One benefit of this research is that you will learn more about how

psychologists study attitudes. You will also receive $.30 for completing this study.

Privacy: None of your responses are connected to your name. We will keep your responses private. We will not make any attempt to link your Mturk ID to any identifiable information that may exist on the Internet. However, the service hosting this survey may have access to your responses and IP number. We cannot guarantee that this service will keep your responses private. Results may be used in future classes and research. Cumulative results from this survey may be presented at conferences and/or published in books, journals, and/or in the popular media.

Participation and withdrawal: Your participation is completely voluntary. You may quit at any time without penalty, besides the loss of the monetary compensation. You can also skip any questions if you prefer not to answer.

Further information: If you have questions about this study, please contact Dr. Dov Cohen (email:dovcohen247@gmail.com), Department of Psychology, University of Illinois, Champaign, IL 61820. Phone: (217) 244-5830.

Who to contact about your rights in this study: If you have any questions about your rights as a participant in this study or any concerns or complaints, please contact the University of Illinois Institutional Review Board at 217-333-2670 or via email at irb@illinois.edu

○ **The purpose and nature of this research have been sufficiently explained and I agree to participate in this study. I understand that I am free to withdraw at any time without incurring any penalty.** (1)

End of Block: Default Question Block

Start of Block: Block 5

Thank you for participating in our study! Today you will be reading over some scenarios and answering questions. Please take your time and read over everything carefully.

End of Block: Block 5

Start of Block: Loan

Display This Question:

If financial = 1

And time = 1

For the next questions, imagine that you are currently making $60,000 per year. You currently have $10,000 in the bank and have been searching for a car to buy.

For the following questions, you will see a car, along with some of its specifications. You will have to review all of the information and indicate your preference.

Display This Question:

If financial = 2

And time = 1

For the next questions, imagine that you are currently making $90,000 per year. You have been searching for a car to buy.

For the following questions, you will see a car, along with some of its specifications. You will have to review all of the information and indicate your preference.

Display This Question:

If financial = 1

And time = 2

For the next questions, imagine that it's 15 years from now and you are making $60,000 per year. You have $10,000 in the bank and have been searching for a car to buy.

For the following questions, you will see a car, along with some of its specifications. You will have to review all of the information and indicate your preference.

Display This Question:

If financial = 2

And time = 2

For the next questions, imagine that it's 15 years from now and you are making $90,000 per year. You have been searching for a car to buy.

For the following questions, you will see a car, along with some of its specifications. You will have to review all of the information and indicate your preference.

Timing
First Click (1)
Last Click (2)
Page Submit (3)
Click Count (4)

Page Break

For all these cars, the dealership is willing to give you a loan. The financing will be from, say, a national bank, a local bank, or the manufacturer.

Please treat each question independently. Your answer as to whether you would buy or not buy one car should not affect your answers for other cars.

Please note that these cars vary in manufacturer, model, mileage, age, and features.

Timing
First Click (1)
Last Click (2)
Page Submit (3)
Click Count (4)

End of Block: Loan

Start of Block: less36base

Year: 2016 Car Type: Four Door Sedan Engine: 2.4 L 4-Cylinder
Basic warranty: 3 yr/36,000 mi
Interior: Cloth Fuel Economy: 25 city/38 highway
Horsepower: 185 hp Transmission: 6-speed automatic

The dealership has agreed to finance the car with a 36 month plan. During the 36 months, you will pay $610 per month. Please indicate how willing you are to purchase the car:

	Definitely don't buy the car 0	50	Definitely buy the car 100
Preference ()			

Timing
First Click (1)
Last Click (2)
Page Submit (3)
Click Count (4)

End of Block: less36base

Start of Block: less40base

Year: 2016 Car Type: Four Door SportWagen Engine: 1.8 L 4-Cylinder
Basic warranty: 3 yr/36,000 mi
Interior: Cloth Fuel Economy: 25 city/35 highway
Horsepower: 170 hp Transmission: 6-speed automatic

The dealership has agreed to finance the car with a 40 month plan. During the 40 months, you will pay $550 per month. Please indicate how willing you are to purchase the car:

	Definitely don't buy the car		Definitely buy the car
	0	50	100
Preference ()			

Timing
First Click (1)
Last Click (2)
Page Submit (3)
Click Count (4)

End of Block: less40base

Start of Block: less44base

Year: 2016 Car Type: Four Door SUV Engine: 1.6 L 4-Cylinder Basic warranty: 3 yr/36,000 mi Interior: Cloth Fuel Economy: 28 city/32 highway Horsepower: 188 hp Transmission: 6-speed automatic

The dealership has agreed to finance the car with a 44 month plan. During the 44 months, you will pay $500 per month. Please indicate how willing you are to purchase the car:

	**Definitely don't buy the car**		**Definitely buy the car**
	0	50	100
Preference ()			

Timing
First Click (1)
Last Click (2)
Page Submit (3)
Click Count (4)

End of Block: less44base

Start of Block: less48base

Year: 2016 Car Type: Four Door SUV Engine: 2.4 L 4-Cylinder Basic warranty: 3 yr/36,000 mi Interior: Cloth Fuel Economy: 19 city/26 highway Horsepower: 173 hp Transmission: 6-speed automatic

The dealership has agreed to finance the car with a 48 month plan. During the 48 months, you

will pay $458 per month. Please indicate how willing you are to purchase the car:

	Definitely don't buy the car		Definitely buy the car
	0	50	100
Preference ()			

Timing
First Click (1)
Last Click (2)
Page Submit (3)
Click Count (4)

End of Block: less48base

Start of Block: less52base

Year: 2016 Car Type: Four Door SUV Engine: 2.5 L 4-Cylinder
Basic warranty: 3 yr/36,000 mi
Interior: Cloth Fuel Economy: 26 city/33 highway
Horsepower: 185 hp Transmission: 6-speed Automatic

The dealership has agreed to finance the car with a 52 month plan. During the 52 months, you will pay $423 per month. Please indicate how willing you are to purchase the car:

	Definitely don't buy the car		Definitely buy the car
	0	50	100
Preference ()			

Timing
First Click (1)
Last Click (2)
Page Submit (3)
Click Count (4)

End of Block: less52base

Start of Block: less56base

Year: 2016 Car Type: Four Door Sedan Engine: 2.5 L 4-Cylinder
Basic warranty: 3 yr/36,000 mi
Interior: Cloth Fuel Economy: 22 city/34 highway
Horsepower: 175 hp Transmission: 6-speed automatic

The dealership has agreed to finance the car with a 56 month plan. During the 56 months, you will pay $393 per month. Please indicate how willing you are to purchase the car:

	**Definitely don't buy the car**		**Definitely buy the car**
	0	50	100
Preference ()			

Timing
First Click (1)
Last Click (2)
Page Submit (3)
Click Count (4)

End of Block: less56base

Start of Block: 36 base

Year: 2016 *Car Type: Four Door Sedan* *Engine: 2.5 L 4-Cylinder*
Basic warranty: 3 yr/36,000 mi
Interior: Cloth *Fuel Economy: 26 city/37 highway*
Horspower: 182 hp *Transmission: 6-speed automatic*

The dealership has agreed to finance the car with a 36 month plan. During the 36 months, you will pay $695 per month. Please indicate how willing you are to purchase the car:

	Definitely don't buy the car		Definitely buy the car
	0	50	100
Preference ()			

Timing
First Click (1)
Last Click (2)
Page Submit (3)
Click Count (4)

End of Block: 36 base

Start of Block: more40base

Year: 2016 Car Type: Four Door SUV Engine: 2.5 L 4-Cylinder
Basic warranty: 3 yr/36,000 mi
Interior: Cloth Fuel Economy: 22 city/31 highway
Horsepower: 168 hp Transmission: 6-speed automatic

The dealership has agreed to finance the car with a 40 month plan. During the

40 months, you will pay $625 per month. Please indicate how willing you are to purchase the car:

	Definitely don't buy the car		Definitely buy the car
	0	50	100
Preference ()			

Timing
First Click (1)
Last Click (2)
Page Submit (3)
Click Count (4)

End of Block: more40base

Start of Block: 44 base

The dealership has agreed to finance the car with a 44 month plan. During the 44 months, you will pay $570 per month. Please indicate how willing you are to purchase the car:

	Definitely don't buy the car		Definitely buy the car
	0	50	100
Preference ()			

Timing
First Click (1)
Last Click (2)
Page Submit (3)
Click Count (4)

End of Block: 44 base

Start of Block: 48 base

Year: 2016 Car Type: Four Door SUV Engine: 2.4 L 4-Cylinder
Basic warranty: 5 yr/60,000 mi
Interior: Cloth Fuel Economy: 21 city/28 highway
Horsepower: 182 hp Transmission: 6-speed automatic

The dealership has agreed to finance the car with a 48 month plan. During the 48 months, you will pay $520 per month. Please indicate how willing you are to purchase the car:

	**Definitely don't buy the car**		**Definitely buy the car**
	0	50	100
Preference ()			

Timing
First Click (1)
Last Click (2)
Page Submit (3)
Click Count (4)

End of Block: 48 base

Start of Block: 52 base

Year: 2016 Car Type: Four Door SUV Engine: 2.5 L 4-Cylinder
Basic warranty: 3 yr/36,000 mi
Interior: Cloth Fuel Economy: 26 city/33 highway
Horsepower: 184 hp Transmission: 6-speed automatic

The dealership has agreed to finance the car with a 52 month plan. During the 52 months, you will pay $480 per month. Please indicate how willing you are to purchase the car:

	Definitely don't buy the car		Definitely buy the car
	0	50	100
Preference ()			

Timing
First Click (1)
Last Click (2)
Page Submit (3)
Click Count (4)

End of Block: 52 base

Start of Block: 56 base

The dealership has agreed to finance the car with a 56 month plan. During the 56 months, you will pay $446 per month. Please indicate how willing you are to purchase the car:

	Definitely don't buy the car		Definitely buy the car
	0	50	100

Preference ()	

Timing
First Click (1)
Last Click (2)
Page Submit (3)
Click Count (4)

End of Block: 56 base

Start of Block: Loan + end instructions

Display This Question:

If financial = 1

And time = 1

For the next questions, imagine that you are currently making $60,000 per year. You currently have $10,000 in the bank and have been searching for a car to buy.

For the following questions, you will see a car, along with some of its specifications. You will have to review all of the information and indicate your preference.

Display This Question:

If financial = 2

And time = 1

For the next questions, imagine that you are currently making $90,000 per year. You have been searching for a car to buy.

For the following questions, you will see a car, along with some of its specifications. You will have to review all of the information and indicate your preference.

Display This Question:

If financial = 1

And time = 2

For the next questions, imagine that it's 15 years from now and you are making $60,000 per year. You have $10,000 in the bank and have been searching for a car to buy.

For the following questions, you will see a car, along with some of its specifications. You will have to review all of the information and indicate your preference.

Display This Question:

If financial = 2

And time = 2

For the next questions, imagine that it's 15 years from now and you are making $90,000 per year. You have been searching for a car to buy.

For the following questions, you will see a car, along with some of its specifications. You will have to review all of the information and indicate your preference.

Timing
First Click (1)
Last Click (2)
Page Submit (3)
Click Count (4)

Page Break

For all these cars, the dealership is willing to give you a loan package. This means the financing will come from a variety of sources (say, partly, from a national bank, partly from a local bank, or partly from the manufacturer).
Conveniently, these loans will all be bundled into 1 package, so that you can make a single payment towards your car. However, because some loans are longer and some are shorter, this means the monthly payment may change over the life of the loan.

Please treat each question independently. Your answer as to whether you would buy or not buy one car should not affect your answers for other cars.

Please note that these cars vary in manufacturer, model, age, and features.

Timing
First Click (1)
Last Click (2)
Page Submit (3)
Click Count (4)

End of Block: Loan + end instructions

Start of Block: less36baseplus190

Display This Question:
If phrase = 1

Year: 2016 Car Type: Four Door Sedan Engine: 2.4 L 4-Cylinder
Basic warranty: 3 yr/36,000 mi
Interior: Cloth Fuel Economy: 25 city/38 highway
Horsepower: 185 hp Transmission: 6-speed automatic

The dealership has agreed to finance the car with a 48 month plan. During the first 36 months, you will pay $610 per month. For the last 12 months, you will pay $150 per month. Please indicate how willing you are to purchase the car:

Definitely don't buy the car		Definitely buy the car
0	50	100

Preference ()	

Display This Question:

If phrase = 2

Year: 2016 Car Type: Four Door Sedan Engine: 2.4 L 4-Cylinder
Basic warranty: 3 yr/36,000 mi
Interior: Cloth Fuel Economy: 25 city/38 highway
Horsepower: 185 hp Transmission: 6-speed automatic

The dealership has agreed to finance the car with a 48 month plan. During the first 36 months, you will pay $610 per month. For the last 12 months, your payments are reduced to $150 per month. Please indicate how willing you are to purchase the car:

Definitely don't buy the car **Definitely buy the car**

0 50 100

Preference ()	

Display This Question:

If phrase = 3

Year: 2016 Car Type: Four Door Sedan Engine: 2.4 L 4-Cylinder
Basic warranty: 3 yr/36,000 mi
Interior: Cloth Fuel Economy: 25 city/38 highway
Horsepower: 185 hp Transmission: 6-speed automatic

The dealership has agreed to finance the car with a 48 month plan. During the first 36 months, you will pay $610 per month. For the last 12 months, your payments decrease to $150 per month. Please indicate how willing you are to purchase the car:

Definitely don't buy the car **Definitely buy the car**

	0	50	100
Preference ()			

Timing
First Click (1)
Last Click (2)
Page Submit (3)
Click Count (4)

End of Block: less36baseplus190

Start of Block: less40baseplus190

Display This Question:
If phrase = 1

Year: 2016 Car Type: Four Door SportWagen Engine: 1.8 L 4-Cylinder
Basic warranty: 3 yr/36,000 mi
Interior: Cloth Fuel Economy: 25 city/35 highway
Horsepower: 170 hp Transmission: 6-speed automatic

The dealership has agreed to finance the car with a 52 month plan. During the first 40 months, you will pay $550 per month. For the last 12 months, you will pay $150 per month. Please indicate how willing you are to purchase the car:

	Definitely don't buy the car		Definitely buy the car
	0	50	100
Preference ()			

Display This Question:
If phrase = 2

Year: 2016 Car Type: Four Door SportWagen Engine: 1.8 L 4-Cylinder
Basic warranty: 3 yr/36,000 mi
Interior: Cloth Fuel Economy: 25 city/35 highway
Horsepower: 170 hp Transmission: 6-speed automatic

The dealership has agreed to finance the car with a 52 month plan. During the first 40 months, you will pay $550 per month. For the last 12 months, your payments are reduced to $150 per month. Please indicate how willing you are to purchase the car:

	Definitely don't buy the car		Definitely buy the car
	0	50	100
Preference ()			

Display This Question:

If phrase = 3

Year: 2016 Car Type: Four Door SportWagen Engine: 1.8 L 4-Cylinder
Basic warranty: 3 yr/36,000 mi
Interior: Cloth Fuel Economy: 25 city/35 highway
Horsepower: 170 hp Transmission: 6-speed automatic

The dealership has agreed to finance the car with a 52 month plan. During the first 40 months, you will pay $550 per month. For the last 12 months, your payments decrease to $150 per month. Please indicate how willing you are to purchase the car:

	Definitely don't buy the car		Definitely buy the car
	0	50	100
Preference ()			

Timing
First Click (1)
Last Click (2)
Page Submit (3)
Click Count (4)

End of Block: less40baseplus190

Start of Block: less44baseplus190

Display This Question:

If phrase = 1

Year: 2016 Car Type: Four Door SUV Engine: 1.6 L 4-Cylinder
Basic warranty: 3 yr/36,000 mi
Interior: Cloth Fuel Economy: 28 city/32 highway
Horsepower: 188 hp Transmission: 6-speed automatic

The dealership has agreed to finance the car with a 56 month plan. During the first 44 months, you will pay $500 per month. For the last 12 months, you will pay $150 per month. Please indicate how willing you are to purchase the car:

	Definitely don't buy the car		Definitely buy the car
	0	50	100
Preference ()			

Display This Question:

If phrase = 2

Year: 2016 Car Type: Four Door SUV Engine: 1.6 L 4-Cylinder
Basic warranty: 3 yr/36,000 mi
Interior: Cloth Fuel Economy: 28 city/32 highway
Horsepower: 188 hp Transmission: 6-speed automatic

The dealership has agreed to finance the car with a 56 month plan. During the first 44

months, you will pay $500 per month. For the last 12 months, your payments are reduced to $150 per month. Please indicate how willing you are to purchase the car:

	Definitely don't buy the car		Definitely buy the car
	0	50	100
Preference ()			

Display This Question:

If phrase = 3

Year: 2016 Car Type: Four Door SUV Engine: 1.6 L 4-Cylinder
Basic warranty: 3 yr/36,000 mi
Interior: Cloth Fuel Economy: 28 city/32 highway
Horsepower: 188 hp Transmission: 6-speed automatic

The dealership has agreed to finance the car with a 56 month plan. During the first 44 months, you will pay $500 per month. For the last 12 months, your payments decrease to $150 per month. Please indicate how willing you are to purchase the car:

	Definitely don't buy the car		Definitely buy the car
	0	50	100
Preference ()			

Timing
First Click (1)
Last Click (2)
Page Submit (3)
Click Count (4)

End of Block: less44baseplus190

Start of Block: less48baseplus190

Display This Question:

If phrase = 1

Year: 2016 Car Type: Four Door SUV Engine: 2.4 L 4-Cylinder
Basic warranty: 3 yr/36,000 mi
Interior: Cloth Fuel Economy: 19 city/26 highway
Horsepower: 173 hp Transmission: 6-speed automatic

The dealership has agreed to finance the car with a 60 month plan. During the first 48 months, you will pay $458 per month. For the last 12 months, you will pay $150 per month. Please indicate how willing you are to purchase the car:

	Definitely don't buy the car		Definitely buy the car
	0	50	100
Preference ()			

Display This Question:

If phrase = 2

Year: 2016 Car Type: Four Door SUV Engine: 2.4 L 4-Cylinder
Basic warranty: 3 yr/36,000 mi
Interior: Cloth Fuel Economy: 19 city/26 highway
Horsepower: 173 hp Transmission: 6-speed automatic

The dealership has agreed to finance the car with a 60 month plan. During the first 48 months, you will pay $458 per month. For the last 12 months, your payments will be reduced to $150 per month. Please indicate how willing you are to purchase the car:

	Definitely don't buy the car		Definitely buy the car
	0	50	100

Preference ()	

Display This Question:

If phrase = 3

Year: 2016 Car Type: Four Door SUV Engine: 2.4 L 4-Cylinder
Basic warranty: 3 yr/36,000 mi
Interior: Cloth Fuel Economy: 19 city/26 highway
Horsepower: 173 hp Transmission: 6-speed automatic

The dealership has agreed to finance the car with a 60 month plan. During the first 48 months, you will pay $458 per month. For the last 12 months, your payments will decrease to $150 per month. Please indicate how willing you are to purchase the car:

	Definitely don't buy the car		Definitely buy the car
	0	50	100
Preference ()			

Timing
First Click (1)
Last Click (2)
Page Submit (3)
Click Count (4)

End of Block: less48baseplus190

Start of Block: less52baseplus190

Display This Question:

If phrase = 1

Year: 2016 Car Type: Four Door SUV Engine: 2.5 L 4-Cylinder

Basic warranty: 3 yr/36,000 mi
Interior: Cloth Fuel Economy: 26 city/33 highway
Horsepower: 185 hp Transmission: 6-speed Automatic

The dealership has agreed to finance the car with a 64 month plan. During the first 52 months, you will pay $423 per month. For the last 12 months, you will pay $150 per month. Please indicate how willing you are to purchase the car:

	**Definitely don't buy the car**		**Definitely buy the car**
	0	50	100
Preference ()			

Display This Question:

If phrase = 2

Year: 2016 Car Type: Four Door SUV Engine: 2.5 L 4-Cylinder
Basic warranty: 3 yr/36,000 mi
Interior: Cloth Fuel Economy: 26 city/33 highway
Horsepower: 185 hp Transmission: 6-speed Automatic

The dealership has agreed to finance the car with a 64 month plan. During the first 52 months, you will pay $423 per month. For the last 12 months, your payments are reduced to $150 per month. Please indicate how willing you are to purchase the car:

	**Definitely don't buy the car**		**Definitely buy the car**
	0	50	100
Preference ()			

Display This Question:

If phrase = 3

Year: 2016 Car Type: Four Door SUV Engine: 2.5 L 4-Cylinder
Basic warranty: 3 yr/36,000 mi
Interior: Cloth Fuel Economy: 26 city/33 highway
Horsepower: 185 hp Transmission: 6-speed Automatic

The dealership has agreed to finance the car with a 64 month plan. During the first 52 months, you will pay $423 per month. For the last 12 months, your payments decrease to $150 per month. Please indicate how willing you are to purchase the car:

	**Definitely don't buy the car**	**Definitely buy the car**
	0 50 100	
Preference ()		

Timing
First Click (1)
Last Click (2)
Page Submit (3)
Click Count (4)

End of Block: less52baseplus190

Start of Block: less56baseplus190

Display This Question:

If phrase = 1

Year: 2016 Car Type: Four Door Sedan Engine: 2.5 L 4-Cylinder
Basic warranty: 3 yr/36,000 mi
Interior: Cloth Fuel Economy: 22 city/34 highway
Horsepower: 175 hp Transmission: 6-speed automatic

The dealership has agreed to finance the car with a 68 month plan. During the first 56

months, you will pay $393 per month. For the last 12 months, you will pay $150 per month. Please indicate how willing you are to purchase the car:

	Definitely don't buy the car		Definitely buy the car
	0	50	100
Preference ()			

Display This Question:

If phrase = 2

Year: 2016 Car Type: Four Door Sedan Engine: 2.5 L 4-Cylinder
Basic warranty: 3 yr/36,000 mi
Interior: Cloth Fuel Economy: 22 city/34 highway
Horsepower: 175 hp Transmission: 6-speed automatic

The dealership has agreed to finance the car with a 68 month plan. During the first 56 months, you will pay $393 per month. For the last 12 months, your payments will be reduced to $150 per month. Please indicate how willing you are to purchase the car:

	Definitely don't buy the car		Definitely buy the car
	0	50	100
Preference ()			

Display This Question:

If phrase = 3

Year: 2016 Car Type: Four Door Sedan Engine: 2.5 L 4-Cylinder
Basic warranty: 3 yr/36,000 mi
Interior: Cloth Fuel Economy: 22 city/34 highway
Horsepower: 175 hp Transmission: 6-speed automatic

The dealership has agreed to finance the car with a 68 month plan. During the first 56 months, you will pay $393 per month. For the last 12 months, your payments decrease to $150 per month. Please indicate how willing you are to purchase the car:

	Definitely don't buy the car		Definitely buy the car
	0	50	100
Preference ()			

Timing
First Click (1)
Last Click (2)
Page Submit (3)
Click Count (4)

End of Block: less56baseplus190

Start of Block: 36 month + 190

Display This Question:

If phrase = 1

Year: 2016 *Car Type: Four Door Sedan* *Engine: 2.5 L 4-Cylinder*
Basic warranty: 3 yr/36,000 mi
Interior: Cloth *Fuel Economy: 26 city/37 highway*
Horsepower: 182 hp *Transmission: 6-speed automatic*

The dealership has agreed to finance the car with a 48 month plan. During the first 36 months, you will pay $695 per month. For the last 12 months, you will pay $190 per month. Please indicate how willing you are to buy the car:

	Definitely don't buy the car	Definitely buy the car

	0	50	100
Preference ()			

Display This Question:

If phrase = 2

Year: 2016 Car Type: Four Door Sedan
Engine: 2.5 L 4-Cylinder
Basic warranty: 3 yr/36,000 mi
Interior: Cloth Fuel Economy: 26 city/37 highway
Horsepower: 182 hp Transmission: 6-speed automatic

The dealership has agreed to finance the car with a 48 month plan. During the first 36 months, you will pay $695 per month. For the last 12 months, your payments are reduced to $190 per month. Please indicate how willing you are to buy the car:

	**Definitely don't buy the car**		**Definitely buy the car**
	0	50	100
Preference ()			

Display This Question:

If phrase = 3

Year: 2016 Car Type: Four Door Sedan
Engine: 2.5 L 4-Cylinder
Basic warranty: 3 yr/36,000 mi
Interior: Cloth Fuel Economy: 26 city/37 highway
Horsepower: 182 hp Transmission: 6-speed automatic

The dealership has agreed to finance the car with a 48 month plan. During the first 36 months, you will pay $695 per month. For the last 12 months, your payments decrease to $190 per month. Please indicate how willing you are to buy the car:

	Definitely don't buy the car		Definitely buy the car
	0	50	100
Preference ()			

Timing
First Click (1)
Last Click (2)
Page Submit (3)
Click Count (4)

End of Block: 36 month + 190

Start of Block: 40 months

Display This Question:

If phrase = 1

Year: 2016 Car Type: Four Door SUV Engine: 2.5 L 4-Cylinder
Basic warranty: 3 yr/36,000 mi
Interior: Cloth Fuel Economy: 22 city/31 highway
Horsepower: 168 hp Transmission: 6-speed automatic

The dealership has agreed to finance the car with a 52 month plan. During the first 40 months, you will pay $625 per month. For the last 12 months, you will pay $190 per month. How willing are you to purchase the car?

Definitely don't buy the car		Definitely buy the car
0	50	100

Preference ()	

Display This Question:

If phrase = 2

Year: 2016 Car Type: Four Door SUV Engine: 2.5 L 4-Cylinder
Basic warranty: 3 yr/36,000 mi
Interior: Cloth Fuel Economy: 22 city/31 highway
Horsepower: 168 hp Transmission: 6-speed automatic

The dealership has agreed to finance the car with a 52 month plan. During the first 40 months, you will pay $625 per month. For the last 12 months, your payments are reduced to $190 per month. How willing are you to purchase the car?

	**Definitely don't buy the car**		**Definitely buy the car**
	0	50	100
Preference ()			

Display This Question:

If phrase = 3

Year: 2016 Car Type: Four Door SUV Engine: 2.5 L 4-Cylinder
Basic warranty: 3 yr/36,000 mi
Interior: Cloth Fuel Economy: 22 city/31 highway
Horsepower: 168 hp Transmission: 6-speed automatic

The dealership has agreed to finance the car with a 52 month plan. During the first 40

months, you will pay $625 per month. For the last 12 months, your payments decrease to $190 per month. How willing are you to purchase the car?

	Definitely don't buy the car		Definitely buy the car
	0	50	100
Preference ()			

Timing
First Click (1)
Last Click (2)
Page Submit (3)
Click Count (4)

End of Block: 40 months

Start of Block: 44 base plus 190

Display This Question:

If phrase = 1

The dealership has agreed to finance the car with a 56 month plan. During the first 44 months, you will pay $570 per month. For the last 12 months, you will pay $190 per month. How willing are you to purchase the car?

	Definitely don't buy the car		Definitely buy the car
	0	50	100
Preference ()			

Display This Question:

If phrase = 2

The dealership has agreed to finance the car with a 56 month plan. During the first 44 months, you will pay $570 per month. For the last 12 months, your payments are reduced to $190 per month. How willing are you to purchase the car?

	Definitely don't buy the car		Definitely buy the car
	0	50	100
Preference ()			

Display This Question:

If phrase = 3

The dealership has agreed to finance the car with a 56 month plan. During the first 44 months, you will pay $570 per month. For the last 12 months, your payments decrease to $190 per month. How willing are you to purchase the car?

	Definitely don't buy the car		Definitely buy the car
	0	50	100
Preference ()			

Timing
First Click (1)
Last Click (2)
Page Submit (3)
Click Count (4)

End of Block: 44 base plus 190

Start of Block: 48 base plus190

Display This Question:

If phrase = 1

Year: 2016 Car Type: Four Door SUV Engine: 2.4 L 4-Cylinder Basic warranty: 5 yr/60,000 mi Interior: Cloth Fuel Economy: 21 city/28 highway Horsepower: 182 hp Transmission: 6-speed automatic **The dealership has agreed to finance the car with a 60 month plan. During the first 48 months, you will pay $520 per month. For the last 12 months, you will pay $190 per month. Please indicate how willing you are to buy the car:**

	Definitely don't buy the car		Definitely buy the car
	0	50	100
Preference ()			

Display This Question:

If phrase = 2

Year: 2016 Car Type: Four Door SUV Engine: 2.4 L 4-Cylinder Basic warranty: 5 yr/60,000 mi Interior: Cloth Fuel Economy: 21 city/28 highway Horsepower: 182 hp Transmission: 6-speed automatic

The dealership has agreed to finance the car with a 60 month plan. During the first 48 months, you will pay $520 per month. For the last 12 months, your payments are reduced to $190 per month. Please indicate how willing you are to buy the car:

	Definitely don't buy the car		Definitely buy the car
	0	50	100
Preference ()			

Display This Question:

If phrase = 3

Year: 2016 Car Type: Four Door SUV Engine: 2.4 L 4-Cylinder Basic warranty: 5 yr/60,000 mi Interior: Cloth Fuel Economy: 21 city/28 highway Horsepower: 182 hp Transmission: 6-speed automatic **The dealership has agreed to finance the car with a 60 month plan. During the first 48 months, you will pay $520 per month. For the last 12 months, your payments decrease to $190 per month. Please indicate how willing you are to buy the car:**

	Definitely don't buy the car	Definitely buy the car
	0 50 100	
Preference ()		

Timing
First Click (1)
Last Click (2)
Page Submit (3)
Click Count (4)

End of Block: 48 base plus190

Start of Block: 52 base plus 190

Display This Question:

If phrase = 1

Year: 2016 Car Type: Four Door SUV Engine: 2.5 L 4-Cylinder Basic warranty: 3 yr/36,000 mi Interior: Cloth Fuel Economy: 26 city/33 highway Horsepower: 184 hp Transmission: 6-speed automatic

The dealership has agreed to finance the car with a 64 month plan. During the first 52

months, you will pay $480 per month. For the last 12 months, you will pay $190 per month. Please indicate how willing you are to purchase the car:

	Definitely don't buy the car		Definitely buy the car
	0	50	100
Preference ()			

Display This Question:

If phrase = 2

Year: 2016 Car Type: Four Door SUV Engine: 2.5 L 4-Cylinder
Basic warranty: 3 yr/36,000 mi
Interior: Cloth Fuel Economy: 26 city/33 highway
Horsepower: 184 hp Transmission: 6-speed automatic

The dealership has agreed to finance the car with a 64 month plan. During the first 52 months, you will pay $480 per month. For the last 12 months, your payments will be reduced to $190 per month. Please indicate how willing you are to purchase the car:

	Definitely don't buy the car		Definitely buy the car
	0	50	100
Preference ()			

Display This Question:

If phrase = 3

Year: 2016 Car Type: Four Door SUV Engine: 2.5 L 4-Cylinder
Basic warranty: 3 yr/36,000 mi
Interior: Cloth Fuel Economy: 26 city/33 highway
Horsepower: 184 hp Transmission: 6-speed automatic

The dealership has agreed to finance the car with a 64 month plan. During the first 52 months, you will pay $480 per month. For the last 12 months, your payments decrease to $190 per month. Please indicate how willing you are to purchase the car:

	Definitely don't buy the car		Definitely buy the car
	0	50	100
Preference ()			

Timing
First Click (1)
Last Click (2)
Page Submit (3)
Click Count (4)

End of Block: 52 base plus 190

Start of Block: 56 base plus 190

Display This Question:

If phrase = 1

The dealership has agreed to finance the car with a 68 month plan. During the first 56 months, you will pay $446 per month. For the last 12 months, you will pay $190 per month. Please indicate how willing you are to purchase the car:

	Definitely don't buy the car		Definitely buy the car
	0	50	100
Preference ()			

Display This Question:

If phrase = 2

The dealership has agreed to finance the car with a 68 month plan. During the first 56 months, you will pay $446 per month. For the last 12 months, your payments will be reduced to $190 per month. Please indicate how willing you are to purchase the car:

	Definitely don't buy the car		Definitely buy the car
	0	50	100
Preference ()			

Display This Question:

If phrase = 3

The dealership has agreed to finance the car with a 68 month plan. During the first 56 months, you will pay $446 per month. For the last 12 months, your payments decrease to $190 per month. Please indicate how willing you are to purchase the car:

	Definitely don't buy the car		Definitely buy the car
	0	50	100
Preference ()			

Timing
First Click (1)
Last Click (2)
Page Submit (3)
Click Count (4)

End of Block: 56 base plus 190

Start of Block: Demographics

Please try to respond as honestly and accurately as possible. Please read each statement carefully.

Have you ever purchased a car?

○ Yes (1)

○ No (2)

How did you pay for the car?

○ Payment Plan (1)

○ Paid cash in full (2)

○ I have never purchased a car. (3)

If you had a payment plan, what were the terms for your loan? (ex. Monthly payment, Duration, Interest Rate)

____

____

____

____

____

In real life, were the cars we have shown you:

	Very different from the cars I would actually purchase in real life	Very similar to the cars I would actually purchase in real life

0 10 20 30 40 50 60 70 80 90 100

()

In real life, were the cars we have shown you:

	Much less expensive than the ones I would actually purchase	About as expensive as the ones I would actually purchase	Much more expensive than the ones I would actually purchase

0 10 20 30 40 50 60 70 80 90 100

()

Display This Question:

If financial = 1

In real life, if I had a $60,000 salary with $10,000 in the bank, I would feel:

	Much poorer than I actually do in real life	About the same as I actually do in real life	Much richer than I actually do in real life

0 10 20 30 40 50 60 70 80 90 100

()

Display This Question:

If financial = 2

In real life, if I had a $90,000 salary, I would feel:

	Much poorer than I actually do in real life	About the same as I actually do in real life	Much richer than I actually do in real life
	0 10 20 30	40 50 60	70 80 90 100
()			

What was your total household income before taxes during the past 12 months?

____

End of Block: Demographics

Start of Block: Block 16

In as much detail as possible, please describe what **you think the purpose** of the study was. If you are not sure, please give **your best guess**.

____

____

____

____

____

Page Break

UNIVERSITY OF ILLINOIS
AT URBANA-CHAMPAIGN
Dr. Dov Cohen
Department of Psychology
College of Liberal Arts and Sciences
603 East Daniel Street
Champaign, IL 61820

DEBRIEFING

The study in which you participated today is part of a project that aims to learn about people's preferences for payment scenarios and how they relate to people's preferences about pain and unpleasantness.

Considering that the citizens in the United States rely heavily on credit and borrowing, investigating why individuals make the financial choices that they do is an important topic to research. To date, there has been little research on the psychology behind why people borrow or how people in the United States view borrowing. With over 1 million filings of bankruptcy per year and 1.5 billion credit cards in the United States, debt is prevalent and common for many citizens.

By conducting this research, we hope to learn which factors contribute to people's financial decisions.

Thank you for participating!

Please do not discuss the details or hypotheses of this study with any other people as they may be potential participants, and knowing the purpose of the study beforehand could affect the results.

If you are feeling distressed as a result of this study and would like to talk to someone, please call 1-800-273-TALK to be connected to a mental health crisis line in your area.

Finally, if you would like any further information or if you have any further questions, please contact Dr. Dov Cohen at dovcohen247@gmail.com.

References for Further Reading

Porter, K. (2012). *Broke: How debt bankrupts the middle class*. Stanford: Stanford University Press.

Additionally, if you would like to increase your own financial literacy and take greater charge of your
finances, we recommend the following:

United States Government Financial educationhttp://www.mymoney.gov/Pages/default.aspx
Consumer Financial Protection Bureau http://www.consumerfinance.gov/
http://www.consumerfinance.gov/students/ (special section for students)

End of Block: Block 16

Supplemental Materials

Preliminary Experiments for Experiments 3 and 4 in the manuscript

Method

Overview. Experiment 3 extends the previous results by testing another counterintuitive prediction of the peak-end rule and relatedly, duration neglect. That is, because people weigh the ending so heavily, a pain sequence which is lengthened by *adding* a somewhat less painful experience at the end is preferred to a sequence without this additional pain. Even though it involves taking more "objective" pain, adding a somewhat less painful ending makes the sequence seem less averse (Kahneman et al., 1993; Redelmeier & Kahneman, 1996). In Experiment 3, we examine whether a parallel effect holds for payment sequences. The hypothesis is that participants' preference for a loan will actually increase, if less "painful," less expensive monthly payments are added on to the end of the payment plan. Though it is a more expensive loan, participants should prefer it because of its "less painful" ending. In addition, we measured participants' intuitions about the aversiveness of unpleasant sequences of physical pain (conceptually replicating Varey and Kahneman, 1992) and examined whether evaluations of pain sequences were correlated with evaluations of payment sequences.

We first describe the basic outline of the three preliminary studies (Experiments 3a-3c), giving a detailed description of Experiment 3a. We then present results for Experiments 3a-3c. Variations included in Experiments 3b or 3c are discussed last.

Experiment 3a

Participants. Three hundred and seventy one participants at the University of Illinois Urbana-Champaign participated in this experiment for course credit. We ran the experiment over the course of one semester and analyzed the data in the summer. Given the sample size of

previous studies and average effect sizes in social psychology, 389 participants would give a power between .5 to over .9, for studies with ds between .2 and .5.

Design and Materials. Participants were told to imagine that they are 2 years out of college, making $60,000 per year, and have $10,000 in the bank. Participants then received a series of car descriptions that included a picture of the car, features of the car, and a payment plan for the car. In total, participants were asked about 18 car pictures and payment plans, 6 of which were between-subject questions and 12 of which were within-subject questions.

For the first set of between-subject car questions, participants were randomly assigned to a condition where they saw 6 "base" (ex. $520 for 48 months) or 6 "base + 12 month" payment plans ($520 for 48 months plus an additional payment of $150 for 12 months). The only difference between the conditions was the payment plan offered, where the "base + 12 months" plans included an additional 12 months of $150 monthly payments. All cars had an MSRP of approximately $25,000 and the 6 "base" payment plans ranged from 42 to 60 months ("Base + 12 months" plans thus took a total of 54 to 72 months). In subsequent studies, the MSRP for the less expensive cars was changed to $22,000.

After responding to the 6 car scenarios, participants were told to imagine that it was 10 years later and that they were making $90,000 per year. Participants were randomly presented with 12 car descriptions - all of the cars had an MSRP of approximately $32,000 and payment plans ranged from 48 to 60 months (i.e., 48 months, 52 months, 56 months, 60 months). In subsequent studies, the MSRP for the more expensive cars was changed to $25,000. There were three different payment scenario "options" for each of the different payment durations: "base" (ex. $535 for 60 months), "base + final payment" (ex. $535 for 60 months plus an additional $2400 in the 61st month), or "base + 12 months" ($535 for 50 months plus an additional 12

months of $200). Each "base + final payment" scenario contained the same final payment, and the "base + 12 months" contained the same additional 12 monthly payments.

For "base + 12 months" surveys, the following sentence, for example, described the end of the loan: "For the 12 months after that, your payments will be reduced to $200 per month." For subsequent studies, we wanted to make sure effects were robust to various wordings. Thus, in each study, participants were randomly assigned to one of 3 versions of the sentence: "For the last 12 months, you will pay…", "For the last 12 months your payments are reduced to…," or "For the last 12 months your payments decrease to…" The effect of phrasing was not significant in any study ($p_{3b} = 0.32$, $p_{3c} = 0.37$, $p_{\text{Experiment 3 in the manuscript}} = 0.78$, $p_{\text{Experiment 4 in the manuscript}} = 0.48$) and so we don't discuss this further.

Participants were then told that the experimenters were interested in people's intuitions about uncomfortable experiences. The instructions were taken from Varey and Kahneman (1992), and participants were told that people were paid to participate in a series of uncomfortable experiences. Every 5 minutes, they made a rating (from 0 to 10) of the discomfort they were feeling at that moment. Participants were told that their task was to provide an overall evaluation of the discomfort for each pain sequence using a scale from 0 to 100 (0 = not bad at all, 100 = extremely bad) (see Varey & Kahneman, 1992 for complete instructions).

For between-subjects questions, participants were assigned to randomly see five "base" (ex. 2 5 4 4 7 9 6 6) or "base + less painful end" pain scenarios (ex. 2 5 4 4 7 9 6 6 2). Participants also rated 18 pain scenarios (within-subject) where sequences contained "base" (ex. 5 5 5 5 5 5 5), "base + less painful end" (ex. 5 5 5 5 5 5 5 2), or "base + more painful end" numbers (5 5 5 5 5 5 5 9).

Surveys ended with participants filling out demographic questions and the individual difference variables described for each of the procedural variations below.

Results

Between-Subject Debt Scenarios. We tested whether participants preferred the "base + additional 12 months" compared to the "base" condition using an independent samples t-test. As may be seen in Table 1, participants generally preferred the more expensive "base + additional 12 months" payment plans over the "base" payment plans. Effect sizes (d) for the studies were: 0.12, 0.17, 0.0. Significance levels were p=0.24, 0.04, 0.90.

Within-Subject Debt Scenarios. We created 2 variables for the within-subject debt scenarios questions. The first variable, "DifferencePlus12months" is the difference between the within-subject "base + 12 additional months" and the "base" cars (i.e., "base + 12 additional months" - "base" cars). The second variable, "DifferencePlusFinal" is the difference between the within-subject "base + final payment" and "base cars (e.g., "base + final payment" - "base" cars). Unlike the between-subjects questions, when participants saw both "base" and "base + 12 months" plans, they preferred "base" plans. As seen in Table 2, effect sizes (d) were: -0.35 (but see caveat below about counterbalancing), -0.03, -0.05; p-values were 0.001, 0.53, 0.10. As expected, participants also preferred the "base" plans to "base + final payment" plans. Effect sizes were -0.76, -0.49, -0.52, all ps=.001.

As noted later, within-subjects cars were randomly assigned to "base" or "base + 12 months" conditions in Experiment 3a, but assignment of cars to conditions was not counterbalanced. From Experiment 3b on, we did counterbalance. Because of the lack of counterbalancing, we think results of Experiment 3a for within-subjects items should be treated with caution.

Between-Subject Pain Scenarios. There were five between-subject pain questions, where participants were randomly assigned to see a “base” (ex. 2 5 4 4 7 9 6 6) or “base plus less painful end” (ex. 2 5 4 4 7 9 6 6 2) pain sequence. In Study 3a, the pain questions were independent of one another so participants saw “base” and “base plus less painful end” scenarios. In subsequent studies, the design was changed so that participants saw five “base” or five “base plus less painful end” scenarios, making it a completely between-subject design. All analyses were conducted with independent sample t-tests. As seen in Table 3, participants tended to find the “base plus less painful end” sequences less odious than the “base” sequences. Effect sizes were: 0.10, 0.06, 0.07; ps were .22, .35, .07.

Within-Subject Pain Scenarios. Similar to the within-subject debt scenarios, we created variables to determine the mean difference between the “base” and “base + less painful end,” as well as the “base” vs. “base plus + more painful end.” The first variable, “Pain Base Minus Low”, computes the difference between the pain “base” scenarios and the “pain plus less painful end” scenarios. The second variable, “Pain Base Minus High” computes the difference between the pain “base” scenarios and the “pain + more painful end” scenarios. Following Varey and Kahneman (1992), we predicted that individuals would rate pain scenarios as more painful if they finished on a “more painful end.” We also predicted that participants would rate the “base plus less painful end” as less painful compared to the “base” scenarios (even though the duration is increased). Given these hypotheses, “Pain Base Minus Low” should be positive and “Pain Base Minus High” should be negative.

As predicted, participants rated the “base + less painful end” as less painful compared to the “base” scenarios, even though the duration of the painful experience was 5 minutes longer. As seen in Supplemental Table 4, effect sizes were 0.13, 0.07, and 0.13. P-levels were 0.02, 0.07,

and 0.001. Also for the more obvious prediction, they found the "base + more painful end" to be worse than "base" sequences. Effect sizes were -0.41, -0.53, and -0.52, all p-levels =0.001.

Correlation between Pain Preference and Debt Preference. We tested whether participants' preferences for loan sequences were predicted by their ratings of painful experiences. The test involves a regression predicting participants' responses to the between-subjects debt questions from Condition ("base" vs. "base + 12 months"), "Pain base minus low" (indicating preference for a "soft landing" on the within-subject pain sequences), and the interaction of these two variables. If there were a relation between individual differences in participants' preference for soft landings in pain and their preference for soft landings in payment, the interaction would be significant. It was not in almost all of the studies; Study 3a (b_{3a} = 7.11, $t_{3a}(375)$ = .76, p_{3a} = 0.45), Study 3b (b_{3b} = 14.27, $t_{3b}(602)$ = 1.42, p_{3b} = 0.16), and Study 3c (b_{3c} = -13.24, $t_{3c}(1099)$ = -1.80, p_{3c} = 0.07). We also examined the correlation of the within-subject debt payments variable (DifferencePlus12Months) and the within-subjects pain variable ("Pain Base Minus Low"). It was not significant (Study 3a (r_{3a} = 0.06, p_{3a} = 0.21), Study 3b (r_{3b} = -0.02, p_{3b} = 0.56), Study 3c (r_{3b} = 0.02, p_{3b} = 0.55).

Variations for Experiment 3b

Method

Participants. 634 participants were recruited on Amazon Mechanical Turk for a small fee. After excluding for duplicate IP addresses and IP addresses not from the United States, 607 participants remained. We tried to approximately double the sample size of Experiment 3a to increase our power and ability to detect a significant effect.

Design and Materials. The design of Experiment 3b was very similar to Experiment 3a, except for the following 1) an additional set of stimuli was included so that all cars for the within-subjects payment plan questions would be in the "base" condition half the time and the "base + 12 months" condition half the time. This was also done in subsequent studies. (Previously, payment plan was randomly assigned to a given car before we began running the study); 2) the duration of the car payment scenarios was decreased -- 32 to 52 months for between-subject car payments and 36 to 52 months for within-subject car payments; 3) participants completed an intuition scale at the end of the study (Hamilton, Shih, & Mohammed, 2016). The scale is 5-items and responses are on a 1-7 scale (1 = strongly disagree, 7 = strongly agree). Example items include "When making decisions, I rely mainly on my gut feelings" and "I make decisions based on intuition." We conducted exploratory analyses to see if people who use an intuitive decision making style are more susceptible to choosing loan plans with "soft landings" even when it is not rational to do so. The design of the between-subject pain questions differed from Study 3a in that participants were assigned to one condition (i.e., "base" or "base + less painful end") rather than receiving a series of questions that were independent of one another. We also added more within-subject pain sequences, so that there were 18 questions total (6 bases).

Results for individual difference predictors

Intuition Scale. For between subject payment plan questions, we tested whether participants' willingness to buy the car was predicted by the intuition scale, "Base" vs "Base + 12 Months" condition, and the interaction of these two. The interaction was not significant (b = -5.19, $t(602) = 0.52$, $p = 0.60$). We also tested this for the within-subject cars. The correlation

between DifferencePlus12Months and the intuition scale was also not significant (r = -0.01, p = .89).

Experience. For between subject payment plan questions, we also examined whether our effects were moderated by experience, such as previously purchasing a car or paying for a car with a payment plan. For between-subject payment plans, we tested whether participants' willingness to buy the car was predicted by previously purchasing a car (0 = No, 1 = Yes), "Base" vs "Base + 12 Months" condition, and the interaction of these two. The interaction was not significant (b = 40.06, t(602) = 1.43, p = 0.15). Similarly, the interaction between paying for a car with a payment plan (1 = Yes, 2 = No – Cash) and "Base" vs "Base + 12 Months" condition was not significant (b = 5.44, t(512) = 0.25, p = 0.80). Level of education also did not moderate effects; if anything, higher education made loans with the extra payments more attractive, but this was not significant (t=1.48, p=.14).

Variation for Experiment 3c

Method

Participants. 1193 participants were recruited on Amazon Mechanical Turk for a small fee. After excluding duplicate IP addresses and IP addresses not from the United States, 1132 participants remained. To determine sample size, we calculated an average weighted Cohen's d of the last two studies (d = -.15) and conducted a power analysis using d = .15, power = .8, and probability level = .05. The minimum subjects needed for 80% power is 1102 for a one-tailed hypothesis.

Design and Materials. As in Experiment 3b, we used the same pain sequences and included the intuition scale as a possible individual difference moderator.

Results for individual difference predictor

Intuition Scale. For the between-subjects payment plan questions, the interaction between the intuition scale and "Base" vs "Base + 12 Months" condition did not significantly predict participants' willingness to buy the car (b = -0.96, t(1106) = -0.13, p = 0.90). The interaction between DifferencePlus12Months and the intuition scale was also not significant (r = -0.004, p = .89).

Experience. For between subject payment plan questions, the interaction between previously purchasing a car (1 = Yes, 0 = No) and "Base" vs "Base + 12 Months" condition did not predict participants' willingness to buy the car (b = -9.85, t(1102) = -0.46, p = 0.64). However, the interaction between paying for a car with a payment plan (1 = Yes, 0 = No – Cash) and "Base" vs "Base + 12 Months" condition was significant (b = -46.30, t(945) = -2.83, p = 0.005). Specifically, participants who had paid for a car with cash preferred the "base + 12 month" cars (M = 229.70, SD = 127.69) to the "base" cars (M = 200.86, SD = 128.19) (simple effect t(363) = 2.15, p = .03, d = 0.23 [.02, .43]; F(1, 946) = 5.06, p = 0.03). However, participants who had paid for a car with a payment plan were marginally more likely to prefer "base" cars (M = 221.75, SD = 117.32) to "base + 12 month" cars (M = 204.60, SD = 120.33) (simple effect t(946) = -1.78, p = 0.09, d = -0.15 [-.31, .02]; F(1, 946) = 2.97, p = 0.09). The "base vs. base + 12 month" X education interaction was not significant (t=-.34, p=.74).

Table 1. Effect sizes for Studies 3a-3c for Between-Subject Debt

Study	N	Base		N	Base + 12 months		Mean Difference, 95% CI	ES (Cohen's *d*), 95% CI	*p*
		Mean	SD		Mean	SD			
Study 3a	202	207.70	91.04	191	218.41	90.04	10.71 [7.34, 28.77,]	.12[.08, .32]	.24
Study 3b	300	201.79	120.91	307	222.52	124.05	20.73 [1.20, 40.27]	.17[.01, .33]	.04
Study 3c	569	215.86	122.89	563	216.82	122.11	.96 [-13.32, 15.25]	.001[-.11, .12]	.90

Table 2. Effect Sizes for Studies 3a-3c for Within-Subject Debt

Study	N	Mean Difference, 95% CI	ES (Cohen's *d*)	*p*
**Study 3a**				
DifferencePlus12Months	389	-24.15 [-31.05, -17.26]	-0.35	<.001
DifferencePlusFinal	389	-50.77 [-57.43, -44.11]	-0.76	<.001
**Study 3b**				
DifferencePlus12Months	605	-2.26 [-9.28, 4.76]	-0.03	.53
DifferencePlusFinal	605	-34.73 [-40.34, -29.13]	-0.49	<.001
**Study 3c**				
DifferencePlus12Months	1118	-4.31[-9.50, .89]	-0.05	.10
DifferencePlusFinal	1118	-35.65[-39.70, -31.60]	-0.52	<.001

Table 3. Effect Sizes for Studies 3a-3c for Between Subject Pain

Study	N	Base		N	Base + less painful end		Mean Difference,	ES (Cohen's *d*),	*p*
		Mean	SD		Mean	SD	95% CI	95% CI	
Study 3b	302	284.91	72.95	305	277.61	73.65	7.30 [-4.39, 18.99]	.10 [-.06, .26]	0.22
Study 3c	553	279.48	74.26	556	275.44	70.84	4.04 [-4.51, 12.59]	0.06 [-.06, .17]	0.35

Table 4. Effect Sizes for Studies 3a-3c for Within-Subject Pain

Study	Sample Size	Mean Difference, 95% CI	ES (Cohen's *d*)	*p*
**Study 3a**				
Base Minus Low	382	5.66 [1.09, 10.22]	0.13	0.02
Base Minus High	384	-18.37 [-22.86, -13.88]	-0.41	<.001
**Study 3b**				
Base Minus Low	604	3.67 [-.32, 7.67]	0.07	0.07
Base Minus High	604	-26.90 [-30.98, -22.83]	-0.53	<.001
**Study 3c**				
Base Minus Low	1105	6.02 [3.22, 8.81]	0.13	<.001
Base Minus High	1103	-25.36 [-28.24, -22.48]	-0.52	<.001
